# Gene expression profiling to characterize sediment toxicity – a pilot study using *Caenorhabditis elegans *whole genome microarrays

**DOI:** 10.1186/1471-2164-10-160

**Published:** 2009-04-14

**Authors:** Ralph Menzel, Suresh C Swain, Sebastian Hoess, Evelyn Claus, Stefanie Menzel, Christian EW Steinberg, Georg Reifferscheid, Stephen R Stürzenbaum

**Affiliations:** 1 Humboldt-Universität zu Berlin, Department of Biology – Freshwater and Stress Ecology, Spaethstr. 80/81, 12437 Berlin, Germany; 2 School of Biomedical & Health Sciences, Pharmaceutical Science Division, King's College London, 150 Stamford Street, London, SE1 9NH, UK; 3 Ecossa (ecological sediment & soil assessment), Giselastr. 6, 82319 Starnberg, Germany; 4 Federal Institute of Hydrology, Department Biochemistry and Ecotoxicology, Am Mainzer Tor 1, 56068 Koblenz, Germany

## Abstract

**Background:**

Traditionally, toxicity of river sediments is assessed using whole sediment tests with benthic organisms. The challenge, however, is the differentiation between multiple effects caused by complex contaminant mixtures and the unspecific toxicity endpoints such as survival, growth or reproduction. The use of gene expression profiling facilitates the identification of transcriptional changes at the molecular level that are specific to the bio-available fraction of pollutants.

**Results:**

In this pilot study, we exposed the nematode *Caenorhabditis elegans *to three sediments of German rivers with varying (low, medium and high) levels of heavy metal and organic contamination. Beside chemical analysis, three standard bioassays were performed: reproduction of *C. elegans*, genotoxicity (Comet assay) and endocrine disruption (YES test). Gene expression was profiled using a whole genome DNA-microarray approach to identify overrepresented functional gene categories and derived cellular processes. Disaccharide and glycogen metabolism were found to be affected, whereas further functional pathways, such as oxidative phosphorylation, ribosome biogenesis, metabolism of xenobiotics, aging and several developmental processes were found to be differentially regulated only in response to the most contaminated sediment.

**Conclusion:**

This study demonstrates how ecotoxicogenomics can identify transcriptional responses in complex mixture scenarios to distinguish different samples of river sediments.

## Background

It is a well established fact that the water quality of rivers is strongly influenced by their sediments. Sediments are frequently highly contaminated because hydrophobic chemicals, introduced to the water body, bind to particles and accumulate in the sediment. In contrast to surface waters, river sediments therefore reflect not only present, but also past contamination. Ignoring their capacity to act as a sink and as a potential source of contamination can lead to wrong conclusions concerning the characterization of current pollution levels. Therefore, sediment quality assessment has to be included as an essential integral part of any environmental risk assessment of freshwater bodies [1].

Detailed chemical analyses and sediment toxicity tests typically expose benthic organisms to bulk sediments to assess their quality [2]. The diversity of toxic substances in the environment, the complexity of possible adverse or even positive responses to exposure, and various biotic and abiotic factors that modulate a response call for a comprehensive approach that is able to analyze simultaneously several thousand measurable variables [3]. Molecular approaches, such as 'ecogenomics' [4] or 'ecotoxicogenomics' [5], may prove to be a suitable tool for facilitating the interpretation of bulk sediment toxicity data, as the molecular response of an organism is arguably more sensitive and more specific than the response at higher levels of organization. Pragmatically, the purpose of eco(toxico)genomics is to identify gene and/or protein classes which are switched on or off upon exposure, thus making it possible to detect molecular fingerprints specific to the bio-available fraction of the chemical contamination.

This study investigated the aptness of the bacterivorous nematode *Caenorhabditis elegans *as a model organism for toxicogenomic sediment testing. Various studies have previously demonstrated the general suitability of *C. elegans *in classical sediment toxicity testing [6-8] and, more recently, also in DNA microarray experiments with clear toxicological background [9-14]. However, to date, the use of microarrays has not been explored to assess sediment ecotoxicology in *C. elegans*. This paper aims to redress this shortfall by identifying changes in the gene expression of *C. elegans *exposed to three German river sediment samples of varying pollution status, namely Danube, Rhine and Elbe. Correlating the chemical composition of sediment with biological toxicity tests and global gene expression will clarify (i) whether expression patterns mirror the different levels of pollution by over-representing regulatory and metabolic pathways as well as gene classes; and (ii) if these findings support, or indeed provide a deep understanding of the biological effects observed that go beyond the classical toxic parameters of DNA toxicity and estrogenicity, as defined by the Comet and YES assays.

## Results

### Test design

The sites at the Danube (Bad Abbach), the Rhine (Bimmen), and the Elbe (Magdeburg), were selected due to differing pollution levels and patterns, previously identified in long-term survey programs and/or research programs operated by the Federal Institute of Hydrology (BfG). To study the reproductive capacity and the gene expression, *C. elegans *were exposed to the sediments of Danube (low contamination), Rhine (moderate contamination), or Elbe (high contamination). Moreover, pore water was obtained from the sediments to perform the Yeast Estrogen Screen (YES) and the comet assay to evaluate estrogenicity and genotoxicity, respectively. In addition to the bioassays, the sediment samples were analyzed for priority chemical contaminants.

### Chemical analyses characterize the Elbe sediment as most polluted

Sediments were analyzed for a variety of priority substances. According to their grain-size distribution, the three sediments are silty or sandy mud with organic contents of 2.1 to 5.3%. Fine grain-sized sediments are particularly suitable to accumulate organic compounds and heavy metals. In general, the sediments showed a clear pollution gradient (Elbe > Rhine > Danube), with the Elbe sediment being characterized by elevated organic contamination and high concentrations of heavy metals (Table 1). The Rhine sediment shows a low to moderate contamination with only copper, nickel and zinc being slightly elevated. The most prominent contaminants in the sediment of the River Elbe are organochlorine compounds. These persistent organic pollutants (POP) are DDT and its metabolites, HCH-isomers, hexachlorobenzene (HCB), octachlorostyrene (OCS) and polychlorinated biphenyls (PCB 28, 52, 101, 118, 138, 153, and 180). The total amounts of 16 PAHs were between 1.34 (Danube) and 6.10 mg/kg (Elbe), the concentrations of TBT ranged between below the detection limit < 2 (Danube) and 11.3 μg Sn/kg (Elbe). Moreover, the highest concentrations of heavy metals were detected in the Elbe sediment. It can be expected that PAHs, PCB, and other POP act as Ah-receptor agonists, whereas organotin compounds display an endocrine disrupting potential.

**Table 1:** Key chemical properties/parameters of the sediments.

	**Danube**	**Rhine**	**Elbe**
**TOC (g/kg)**	32	21	53

**N (g/kg)**	3	2	3

**S (g/kg)**	<2	<2	<2

**d.w. (%)**	42.2	38.3	40.3

**F1 (> 2000 μm)**	1.0	2.0	1.0

**F2 (630–2000 μm)**	2.0	2.0	2.0

**F3 (200–630 μm)**	3.0	50.0	11.0

**F4 (63–200 μm)**	11.0	13.0	8.0

**F5 (20–63 μm)**	13.0	6.0	27.0

**F6 (< 20 μm)**	71.0	27.0	52.0

**TPH (mg/kg)**	170	110	270

**Σ 16 EPA PAH (mg/kg)**	1.34	3.06	6.10

**Σ 7 PCB (μg/kg)**	19.2	28.0	44.7

**α-HCH (μg/kg)**	0.09	0.10	430

**β-HCH (μg/kg)**	0.12	0.94	230

**γ-HCH (μg/kg)**	0.61	0.32	56

***p, p'*-DDT (μg/kg)**	0.17	0.81	56

***p, p'*-DDD (μg/kg)**	0.76	1.10	110

***p, p'*-DDE (μg/kg)**	1.50	2.00	33

**HCB (μg/kg)**	1.60	10	56

**OCS (μg/kg)**	0.11	0.33	6.30

**TBT (μg Sn/kg)**	4.0	< 2.0	11.3

**As (mg/kg)**	10.4	18.6	41.3

**Pb (mg/kg)**	29.9	89.6	144.0

**Cd (mg/kg)**	0.64	1.26	6.9

**Cr (mg/kg)**	56.5	91.5	102.0

**Cu (mg/kg)**	51.2	70.2	136.0

**Ni (mg/kg)**	36.1	61.5	65.4

**Hg (mg/kg)**	0.48	0.5	1.5

**Zn (mg/kg)**	270	490	1060

All concentrations are units per kg dry sediment and derived from a single measurement with appropriate analytical controls. The sediment volumes differ depending on which analysis was performed and the degree of contamination (typically, between 100 mg and 2 g of dried sediment). Abbreviations of organic pollutants: TPH – Total Petroleum Hydrocarbon; Σ 16 EPA PAH (; Σ7 PCB -Ballschmitter No. 28, 52, 101, 118,138,153,180; HCH – Hexachlorcyclohexane; DDT – Dichlorodiphenyltrichloroethane; DDD – Dichlorodiphenyldichloroethane; DDE – Dichlorodiphenyldichloroethylene; HCB – Hexachlorobenzene; OCS – Octachlorostyrene; TBT – Tributyltin. Metal measurements were performed on the < 20 μm fraction.

### Ecotoxicological biotests confirm that the Elbe sediment is most toxic

#### Nematode toxicity test

The whole sediment toxicity test with *C. elegans *distinguishes the river Danube from the rivers Rhine and Elbe (Table 2). When exposed for 96 h to Rhine and Elbe sediments, inhibition of reproduction was found to approximate the toxicity threshold of 50%, clearly indicating the presence of sublethal toxicity. In contrast, only a low level inhibition of reproduction was observed in the Danube sediment suggesting that this sample is less toxic. The fact that even in the polluted sediments *C. elegans *was affected but not killed was a prerequisite for the microarray study.

**Table 2:** Whole sediment toxicity (reproduction tests with *Caenorhabditis elegans*), genotoxicity in pore water (Comet assay), and estrogenic activity in pore water (YES = yeast estrogen screen).

	**Control**	**Danube**	**Rhine**	**Elbe**
**Toxicity test **^**a**^				
reproduction	133.7 ± 21.7	90.8 ± 13.4	58.9 ± 16.4	69.3 ± 17.8
% inhibition	0	32.1	55.9**	48.1**

**Comet Assay **^**b**^				
25% pore water	7.64 ± 1.06	8.72 ± 0.63	9.28 ± 1.05	10.86 ± 0.54
50% pore water	7.06 ± 0.43	11.59 ± 1.84	13.50 ± 0.80*	13.50 ± 0.80*

**YES test **^**c**^				
EEQ [ng/L]	^-^	9.0 ± 2.26	18.8 ± 5.33	31.6 ± 11.6***

^a ^The toxicity test is based on the mean of four replicate experiments

^b ^% tail DNA (% vitality) was calculated of 100 randomly selected nuclei on two replicate slides;

^c ^EEQ values were calculated from three independent experiments (performed using 50% pore water), each consisting of three replicates;

* Significantly different to the negative control (p < 0.05);

** Significantly different to the negative control (p < 0.01);

*** Significantly different to the Danube sample (p < 0.05).

#### Comet assay

All samples had slight to significant DNA damaging potential in RTG-2 cells indicating that genotoxic substances are present in the pore water of the sediments. Consistent with the chemical contamination, the Elbe sample was most genotoxic followed by the ones from Rhine and Danube (Table 2). In each case, a clear concentration effect relationship was obvious with the highest genotoxicity in the most concentrated (50%) sample. Interference of cytotoxicity could be excluded due to the utilization of a double cytofluorescence assay, which involved simultaneous staining of cells with FDA and PI. Even in the most polluted sample (Elbe), the viability of the cells was equal or above 88%, which is in line with the general consensus that a sample is regarded as being non-toxic if cell viability is above 80% of controls [15].

#### YES test

Estrogen receptor binding potential was found in all pore water samples. Independent experiments on three consecutive days revealed mean values of 31.6 ng/L ethinyl estradiol equivalents (EEQ) for the Elbe sample, 18.8 and 9.9 ng/L for Rhine and Danube samples, respectively (Table 2). Mean background toxicity, measured as growth reduction of yeast, was negligible in all samples. The estrogenic potential differed considerably between the three sites, with a significant difference (p < 0.05; Tukey's HSD test) between Elbe and Danube samples.

Due to the low chemical burden and the corresponding biotest data, Danube sediment was confirmed to be a suitable reference sediment for further experiments. It should be noted that the use of artificial, unpolluted control sediments [16] or laboratory growth media are not without their own challenges, and will be addressed later in more detail.

### *C. elegans *exhibit reproducible transcriptional profiles when exposed to river sediments

**Figure 1:**
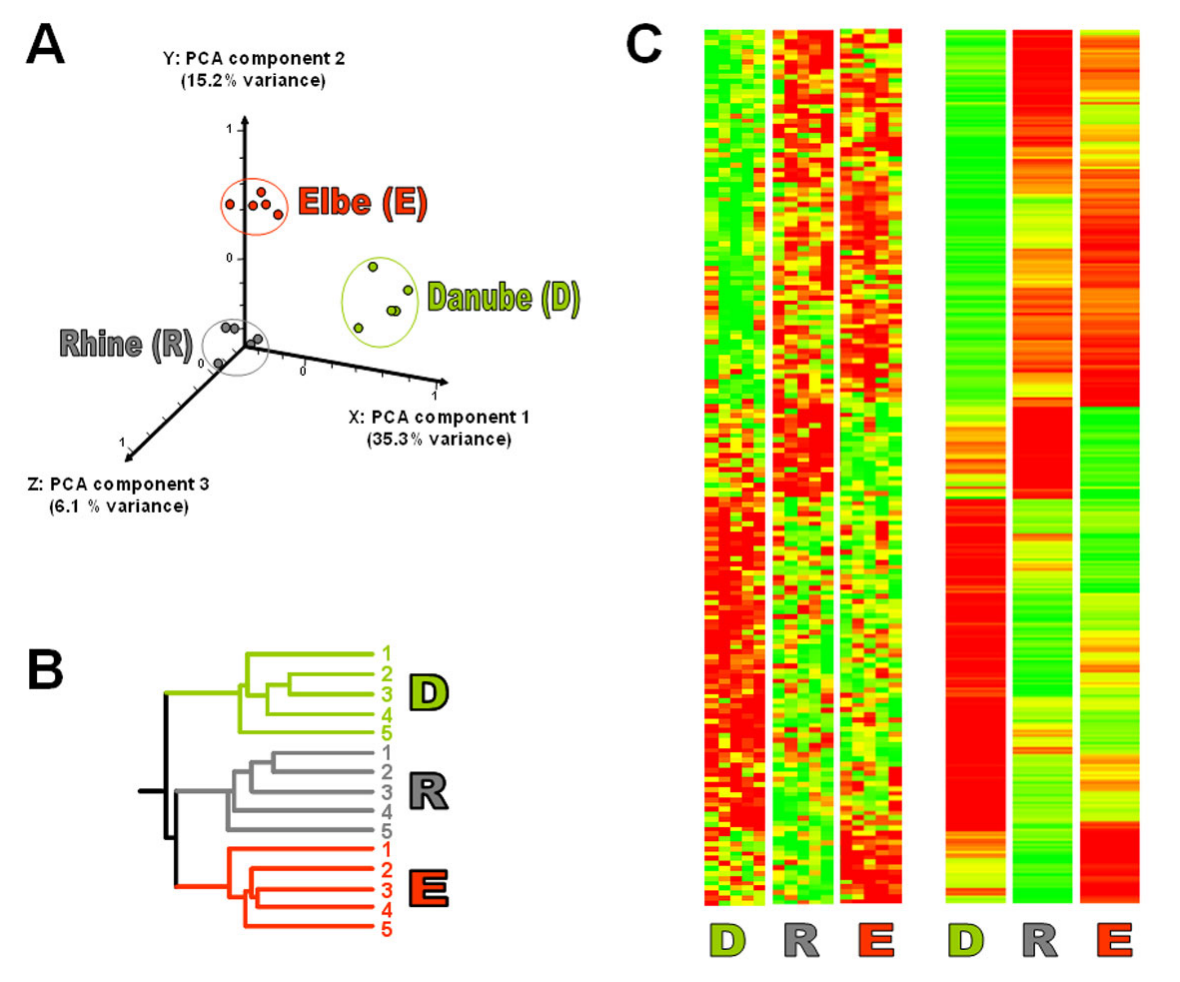
**Hierarchical cluster analysis**. Transcript profiles of 1331 *C. elegans *genes, which were found to be significantly differentially expressed as result of Elbe and/or Rhine sediment exposure (ANOVA, n = 5, p < 0.05, >1.4-fold change). The individual figures illustrate the relationship between the sediment samples to the reference (Danube sediment). (A) Principal components analysis, the first three components are included. (B) Condition tree demonstrating the relationship of the 15 individual replicates. (C) Hierarchical cluster analysis showing individual replicates (n = 5, left) and group averages (right). D – Danube sediment, E – Elbe sediment sample, R – Rhine sediment sample.

**Figure 2:**
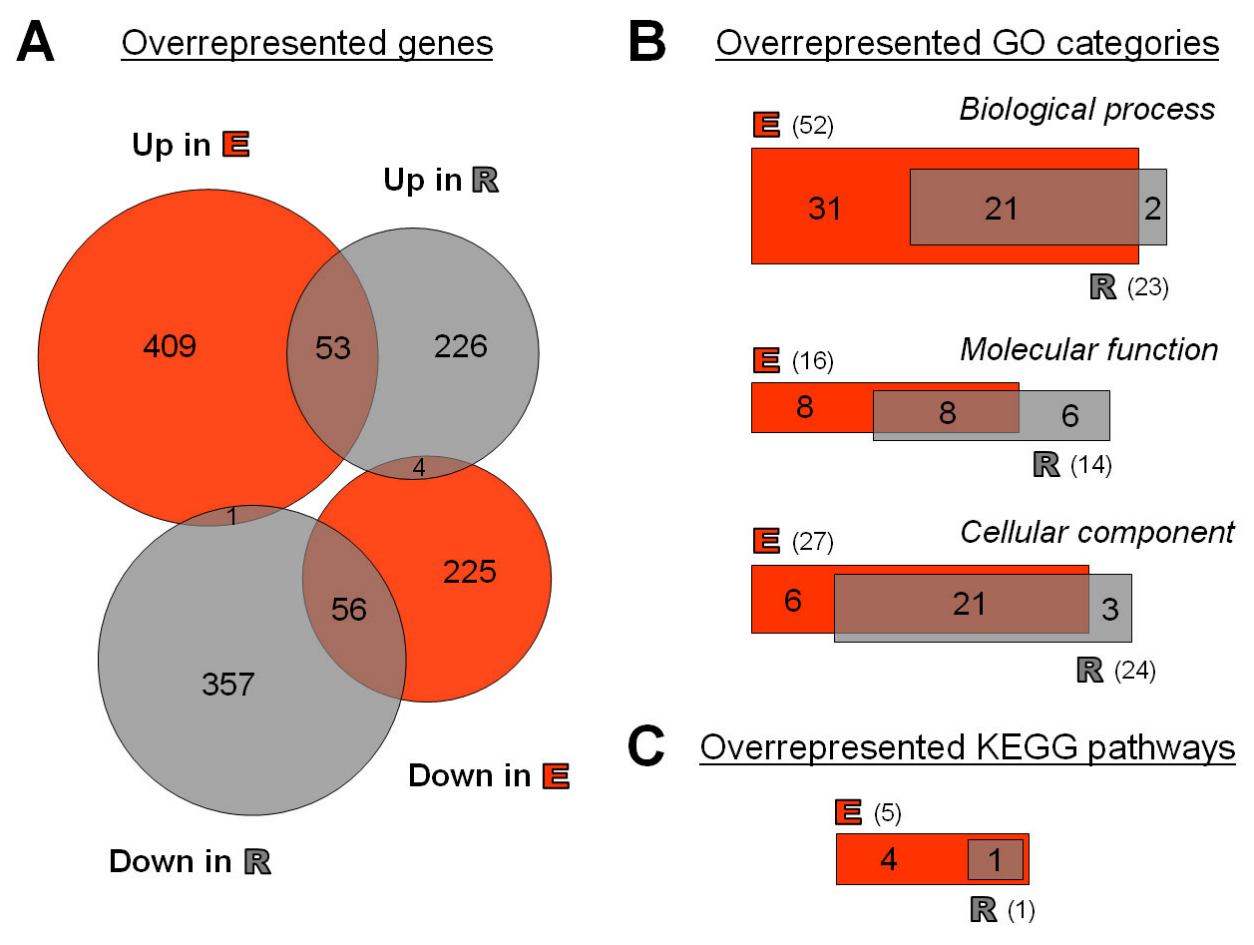
**Overrepresented genes in Venn diagrams**. Overview of genes and functional categories overrepresented in response to Elbe and/or Rhine sediment exposure. (A) Venn diagram showing the overlap of significantly up- and down-regulated genes (ANOVA, n = 5, p < 0.05, >1.4-fold change). Significantly overrepresented GO categories (B) and KEGG pathways (C) were created using Microarray Analysis Tools (p < 0.01, at least three members of an individual KEGG pathway). E – Elbe sediment sample, R – Rhine sediment sample.

The molecular genetic impact of the different river sediments on staged (synchronized) young adult *C. elegans*, exposed to the sediments for 48 h, was determined using a whole genome DNA microarray screening approach. The 19,873 oligonucleotides on the DNA microarray represent 20,445 transcripts of the *C. elegans *genome. Of these, 18,653 yielded good quality spots, of which following normalization, 13,078 genes passed stable and uniform log normal distribution settings (see Methods for details on data normalization and statistical analysis). Using Danube sediment as reference identified 4742 (Elbe) and 4999 (Rhine) transcripts that were differentially regulated by >1.4-fold. One-way ANOVA revealed that 748 and 697 transcripts were significantly (p < 0.05) altered in Elbe and Rhine, respectively (see Additional files 1 and 2). The complete dataset can be viewed in the National Center for Biotechnology Information's (NCBI) Gene Expression Omnibus (GEO) database (accession number GSE11837). The data was subjected to hierarchical clustering analyses (Figure 1). A principal component analysis (PCA) was imposed on the dataset to capture the cluster structure prior to further analysis, mainly to reduce the complexity of the data whilst simultaneously filtering out noise [17]. It shows that the five independent repetitions of each experimental trial are clustered closely together (Figure 1A, B), underlining the presence of a sound technical reproducibility. Graphic heatmaps present an entire overview of all significantly changing genes (Figure 1C); visualized by graduated green (down-regulated), yellow (unchanged) and red (up-regulated) colors. This analysis clusters genes that display similar expression patterns. A corresponding Venn diagram (Figure 2A) illustrates the total number of genes which are significantly up- or down-regulated (≥ 1.4-fold) and reveals an increase in the number of up-regulated genes in nematodes exposed to the Elbe sediment. In contrast, exposure to the Rhine sediment resulted in an increase in down-regulated genes. Interestingly, there is only a moderate overlap of genes that are differentially expressed in both sediments, namely 53 up- and 56 down-regulated genes.

### *C. elegans *exhibit specific functional gene classes overrepresented in response to river sediment exposure

**Figure 3:**
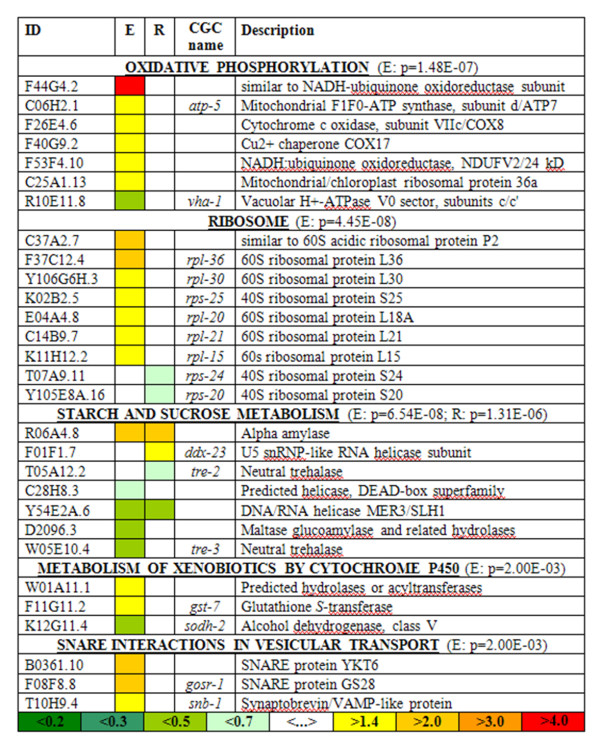
**Overrepresented KEGG pathways**. Identification, direction of change and description of 29 genes identified as members of overrepresented KEGG pathways.

The dataset was dissected further to identify categories that comprise different functionally related objects. This was achieved by Gene Ontology (GO) and Kyoto Encyclopedia of Genes and Genomes (KEGG) pathway screens. Both GO [18] and KEGG [19] are now an integral part of microarray analysis [20-22] as it is capable of extracting and summarizing information from expression data. Simple Venn diagrams (Figure 2B) summarize the number of significant overrepresented GO categories and show the level of correlation between the Rhine and Elbe samples, with a complete list of all significantly regulated GO categories shown in Additional file 3. This substantiates that both sediments display a distinctive similarity in cellular component groups but are only moderately correlated in terms of overrepresented molecular functions (see Additional files 4 and 5). Focusing on the 109 overlapping differentially regulated genes (listed in Additional file 6) these similarities become apparent by a down-regulation of catalytic activity, binding and metabolic process (GO:0003824, GO:0005488 and GO:0008152) as well as, the bi-directed regulation of cell and cell part components (GO:0005623 and GO:0044464). Further evaluation of differentially regulated biological processes also reveals clear-cut differences between the nematode's responses to Rhine and Elbe sediments. The Elbe sediment modulates over twice as many categories as the Rhine sediment. This notion is supported by the KEGG pathway analysis where five KEGG pathways are overrepresented in response to Elbe sediment exposure, but only one pathway (starch and sucrose metabolism) in response to the Rhine sediment (Figure 2C; Figure 3). Elbe and Rhine, but not of Danube sediments, induce several phase 1 or phase 2 metabolism genes (including P450s, GSTs, UGTs) or heat shock proteins (Additional files 1 and 2) and differentially regulate genes that overlap with other ecotoxicogenomic studies (Additional file 8). Moreover, GO categories GO:0016491 (Oxidoreductase activity), GO:0016787 (Hydrolase activity) (Additional file 3) and the KEGG pathway 'Metabolism of xenobiotics by cytochrome P450' were identified to be differentially regulated in the Elbe sediment derived sample. The Elbe sediment consistently induces the expression of ribosomal genes which may reflect an increased synthesis of ribosomes. Likewise, the process of oxidative phosphorylation is seemingly affected, as six members, including cytochrome c oxidase and F1F0-ATP synthase, are up-regulated but only one (*vha-1*) found to be down-regulated. Furthermore, though with a lower level of significance, members of the "metabolism of xenobiotics by cytochrome P450" and "SNARE interactions in vesicular transport" pathways were found (with the exception of *sodh-2*) to be up-regulated. It is noteworthy, that both exposures resulted in a down-regulation of several members of the starch and sucrose metabolism, including the trehalase encoding genes *tre-2 *and *tre-3*. In contrast, the expression of an α-amylase, known to facilitate the breakdown of glycogen in humans, was highly elevated. Both glycogen and trehalose are known to be essential for the maintenance and correct functioning of physiological processes in nematodes.

**Figure 4:**
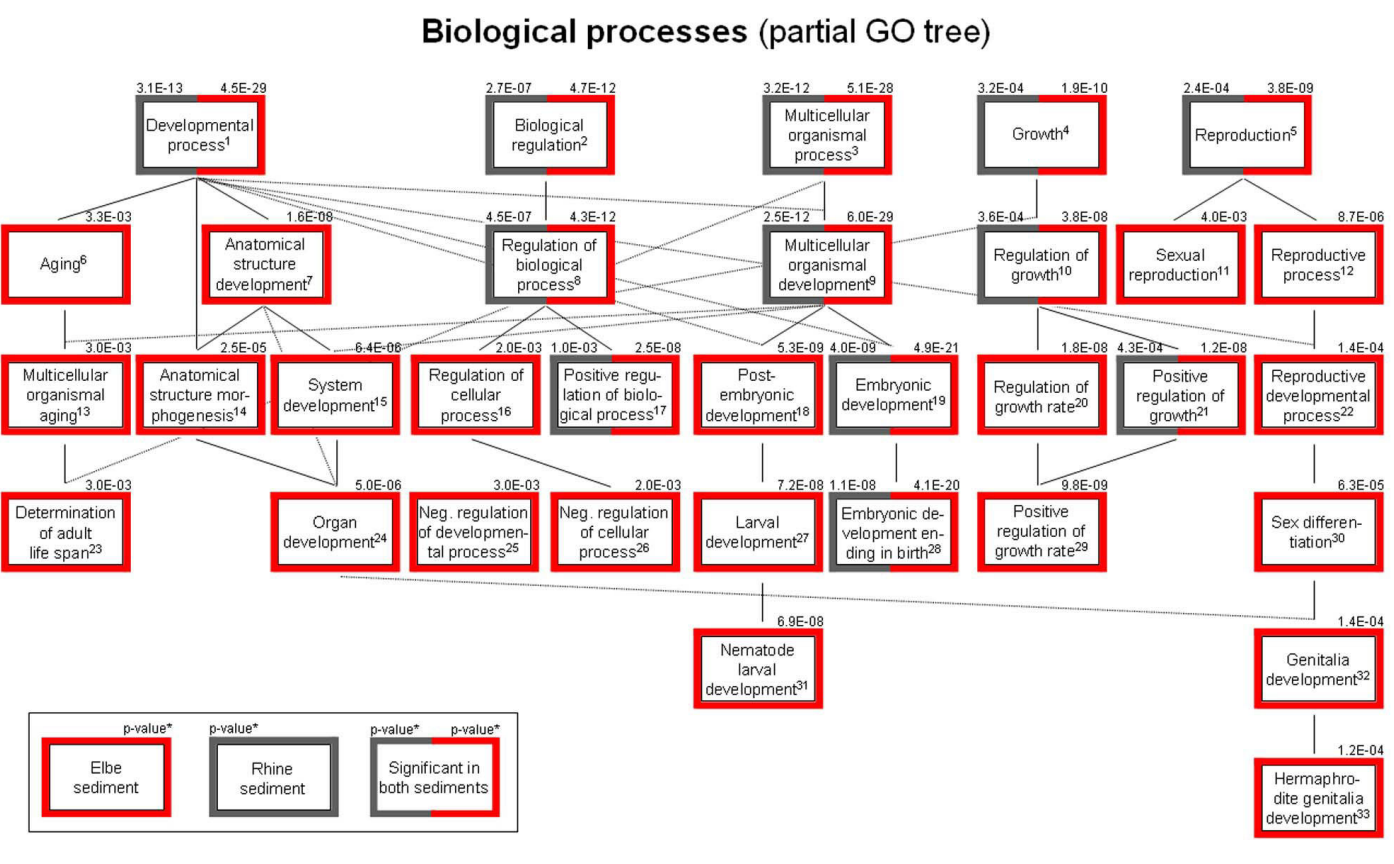
**Biological processes – partial GO tree**. Partial GO tree presenting relevant biological processes which were found to be overrepresented in *C elegans *exposed to Elbe and/or Rhine sediment.

Numerous differentially regulated parent GO terms and related derived daughter terms were analyzed in detail with a sub-selection of overrepresented biological processes illustrated in Figure 4 (a complete list of all significantly regulated GO categories is shown in Additional file 3). The diagram depicts that the majority of derived GO categories are only overrepresented in the Elbe sediment. This indicates that in particular the biological processes of "reproduction", "determination of adult life span" and several developmental processes (such as organ, larval and genitalia development) are differentially modulated by the Elbe sediment. A more detailed dissection focusing on six prominent categories substantiated this notion (Additional file 7). This lists the identity and brief description of the 58 genes that responded to the Elbe and Rhine sediments. In almost all categories a similar number of genes were up- as well as down-regulated, making a further analysis more challenging. Therefore it is currently only possible to speculate that several developmental processes affect *C. elegans *upon exposure to the Elbe sediment – only the process of aging provides more detailed cues, where most of the significantly regulated genes were found to be up-regulated. Finally, it is reassuring to observe that GO terms associated with reproduction are overrepresented in Rhine and Elbe, a finding that correlates well with the observed reduction in brood size in nematodes exposed to the respective sediments.

### Similarities of gene expression patterns in nematodes exposed to Elbe sediment and PCB52 treatment

The gene list of differentially regulated transcripts was compared to past toxicogenomic data where *C. elegans *were exposed to single xenobiotic compounds, PCB52 [11], Cd [12], or two humic substances [10]. Although the current data showed only limited overlap to the differential gene-lists obtained from humic substances and Cd exposures (with 10 or less matches), several genes matched those identified upon treatment to PCB52. In detail, 36 up- and 9 down-regulated genes were common to the Elbe and PCB52 gene list and 28 up- and 14 down-regulated genes to the Rhine list. Noteworthy is that both sediment samples induced the NADH-cytochrome b5 reductase encoding gene. Moreover, *wrn-1 *and *rpa-1*, both coding for proteins involved in DNA damage checkpoint functions, were found to be significantly induced by the Elbe sediment. Furthermore, the PCB52 inducible *cyp-35A5 *was found to be up-regulated in the Rhine sediment derived sample. C-type lectin (*clec-47*) was down-regulated when exposed to either sediment sample, PCB52 or Cd. A complete overview of matching genes is shown in Additional file 8.

## Discussion

Rivers all over the world have a history of being heavily polluted by untreated waste waters from industrial and municipal discharges. Chemical analyses of priority pollutants are typically used to monitor contamination levels and are complemented by bioassay-based approaches and their modifications to serve as simple, rapid and sensitive screening systems to predict bioavailability [23]. Changes in expression levels of single biomarkers (e.g. heat shock proteins, vitellogenins, or cytochromes P450) have been reported to reflect the toxic response and the underlying mode of action of few specific substances or environmental contaminants [24]. The use of DNA microarrays attempts to link ecotoxicological effects with the changes in global gene expression patterns [4,5]. This global approach is expected to cope more adequately with the complex matrices (such as soils and sediments) and to display the variety of potential modes of actions and strategies of the exposed organisms. Clearly, many species of invertebrates could be used to investigate transcriptional responsiveness in sediments, however two challenges need to be met, firstly few invertebrate species are omnipresent (and restricted to local habitats), and secondly none have, to date, been fully sequenced thus prohibiting the application of whole genome microarray technology. In contrast, the genome sequence of *C. elegans *has been deciphered in fine detail, clearly representing an added value to comprehensive ecotoxicogenomics. Although *C. elegans *is primarily a soil nematode, it has also been isolated from freshwater habitats (such as sediments) [25,26]. Given that its natural habitat is the interstitial space between particles filled with water, the exposure to contaminants is similar in soils and sediments, thus making *C. elegans *a suitable test organism for toxicity tests for both habitats. In the laboratory, the use of *C. elegans *has been validated for single compounds, such as steroid hormones, PCB52, Cd, DEHP, as well as natural mixtures, such as humic substances [9-14]. However, due to differences in experimental setups, e.g. the use of mixed populations vs. synchronized cultures or varying exposure times, the systematic and sound comparison between experiments is challenging and highlights, if anything, the need to streamline ecotoxicogenomic experiments. Of course, the use of *C. elegans *in sediment toxicity is not a new concept introduced by us, but supported by numerous publications in classical sediment toxicity testing [6-8], which have been instrumental for the implementation of the first standardized sediments toxicity test (ISO/DIS 10872 Draft).

The chemical analyses and three bioassays (reproduction, estrogenicity, genotoxicity) showed a clear pollution and toxicity gradient, with Elbe sediment being the most polluted characterized by elevated organic and heavy metal contamination. The YES test and comet assay identified the estrogenic and genotoxic potential of the Elbe sediment to be well above that of the Rhine and Danube sediment, respectively.

A laboratory media control (K-medium) was initially included, but deemed too artificial as subsequent PCA analysis identified the laboratory control exposures to cluster distinctly separate from the sediment exposures (see Additional file 9) which masked the differences between the three sediments and thus diminished the robustness of GO and KEGG analyses and biologically meaningful data interpretation (data not shown). Sediments from the three geographically separated rivers are of course heterogeneous with distinct physico-chemical properties which may modulate effects (toxicities) of chemical contamination, a notion that may apply to any control sediment as well (such as the Danube). It could therefore be argued that observed effects may be a combination of chemical contamination and physio-chemical properties of sediments from different rivers. Nevertheless, for reasons of ecological relevance, and justified by the low chemical burden as well as the results from the bioassays, we selected the Danube sediment as reference for the DNA microarray study.

Of course, true DNA damaging or estrogen responsive effects of sediments are best assessed exploiting one of the many budding *in vivo *techniques, including PCR based approaches to detect DNA damage in *C. elegans *[27,28] or to assess changes of estrogen responsive genes in *C. elegans*, such as vit-2 or vit-6 [29]. However, the focus of this work was not to determine the definitive toxic status of the river sediments, but to observe, in a proof of principle experiment, if changes in global gene expression patterns can be used to study (and differentiate) the responses of exposure to complex sediments. Nematodes, as any other organism, exposed to contaminated environments will modulate their metabolic resources and available energy to combat the environmental insult [30]. In line with this, we found that *C. elegans *exposed to Elbe or Rhine sediments down-regulated higher-ranking GO categories which include catalytic activities, binding and metabolic processes. An analogous strategy of *C. elegans *was observed by DNA microarray studies following single compound exposures to PCB52 [11] and Cd [12]. Interestingly, exposure to both sediments resulted in a down-regulation of several members of the starch and sucrose metabolism pathway, including the trehalase encoding genes *tre-2 *and *tre-3*. The disaccharide trehalose is an invertebrate sugar transport and storage material [31] that is present in *C. elegans *at all life stages. The highest concentrations of trehalose (up to 2.3% of dry weight) are found in eggs and dauer larvae, two diapausing stages that are highly resistant to environmental stressors [32], it is proposed to function as energy reserve and stress protectant [33]. In contrast, the gene expression of α-amylase, whose yeast and human orthologs help to facilitate the breakdown of glycogen [34], was found to have strongly increased. Although the Cd level of the Elbe sediment was found to be 5-fold higher than the Rhine sample, the sediment data presented here is remarkably divergent to the global gene expression pattern observed in laboratory exposures to Cadmium [12]. This underlines how single compound exposures, though valuable in their own right, cannot model conditions of true environmental complexity. Observed effects are a sum of effects of chemical contamination and their bioavailability as well as further biotic and abiotic properties of sediments which may mask typical single compound transcript profiles. Indeed, the comparison of several gene lists resulted in only one obvious match, namely with PCB52 responsive genes, including two DNA damage checkpoint protein encoding genes (*wrn-1*, *rpa-1*), which were found up-regulated in the Elbe derived sample, where the sediment was characterized by the highest measured genotoxicity and a high level amount of various POPs including different PCB compounds. In this context it is notable that the pathway involved in metabolism of xenobiotics by cytochrome P450 was induced in those nematodes exposed to the Elbe sediment, whereas the Rhine sediment failed to induce this pathway.

Besides well defined and annotated KEGG pathways, the Elbe sediment exposure resulted in the differential regulation of several related biological processes (GO categories), including development (organ, larval and genitalia development), aging (determination of adult life span) and a pool of 50 GO terms associated with reproduction. Detailed analysis of the latter GO terms will provide a detailed mechanistic and physiological/toxicological understanding of sediment toxicity and allow the linking of gene expression to higher level effects such as the observed reduction in brood size. Almost all members belonging to these categories are characterized by an abnormal developmental and/or aging phenotype (typically Age, Let, Emb, Ste, Gro, Egl, Lva; please find detailed information by referring to ), when silenced by RNAi. Given the significant estrogenicity of the Elbe sediment sample, as defined by the YES test, it is conceivable that at least some of the observed responses are due to this specific class of pollutants. Similarly, exposure to the Elbe sediment was shown to increase the aging process. This process is the determination of adult life span and provides a well-defined data record. First, genes deemed to be essential for embryogenesis and normal growth (*bec-1*, *sip-1*, *abi-1*) [35-37] or associated with an increased life-span (*old-2*) [38] were up-regulated. Second, the loss of *daf-2 *and *nhx-2*, found to be down-regulated, are known to promote a long life as well [39,40]. Likewise, a gain of function of *wrn-1 *[41], found to be up-regulated in this experiment, has been shown to shortened life span. This pattern of regulation, and the results concerning starch and sucrose metabolism pathway, discussed above, are expected to increase nematode's life span and induce the dauer stadium, well known as a survival pathway to overcome unfavorable environmental conditions.

## Conclusion

In summary, our results give a first insight into how microarrays can be used to evaluate differences in gene expression levels in *C. elegans *exposed to differently polluted river sediments. The chemical burden and toxicity of Elbe sediment sample were found to coincide with a substantially higher number of differentially regulated gene classes constituting the GO category of biological processes as well as several KEGG pathways. However, it has to be noted that river sediments are highly complex and vary in abiotic and biotic properties, thus significantly contributing to the modulation of baseline expression profiles. Ecotoxicogenomics should not aim to replace conventional aquatic monitoring techniques, but acts as a supplementary biochemical assay to unravel unknown mode of action pathways. The caveat of the pilot experiment, as disseminated here, is the limited sample number. Further replicates that go beyond the sampling strategy defined by DIN 38414 are needed to be able to fully comprehend the toxicity and associated effects of samples used in this study. Also, large databases comprising numerous expression profiles of individual, pure chemicals, simple mixtures, pore water extracts and in particular further polluted and unpolluted control samples with replicated sampling from the same site are called for, a notion that goes beyond the scope of this pilot study. Nevertheless, this contribution provides tantalizing insights of the potential of microarrays in "real word" ecotoxicogenomics.

## Methods

### Sampling sites

Fine grain-sized sediment samples were collected from three major German rivers in spring 2006. The sampling site Bad Abbach is an appendix-like oxbow lake of the river Danube and located at stream kilometer 2402.6. During low flow conditions, this oxbow lake does not receive much water from the river. Furthermore, the upstream catchment is not heavily industrialized, so that this site is characteristic of a low pollution status. The sampling site Bimmen at the river Rhine is positioned at stream kilometer 863.6 in close proximity to the German-Dutch border. This site reflects the pollution of the Rhine with some heavy metals and PCBs slightly elevated. The site Magdeburg of the river Elbe is located in a groyne field at stream kilometer 319.4. This sampling site is strongly affected by the upstream industrial areas, particularly in the catchments of the tributaries Saale and Mulde. It is one of the most polluted sites of the river Elbe, heavily contaminated with a variety of organic compounds and heavy metals.

Sediments (approximately the top 10 cm) were collected with a stainless steel van Veen grab. To minimize the effects of variability, at least three subsamples were taken and combined. Following the removal of large debris and subsequent homogenization on site, samples were transported in closed stainless steel containers to the laboratory. The supernatant of sediment pore water was collected following the centrifugation at 26,000 g for 20 min. For the YES tests and the Comet assays, pore water samples were filter sterilized using cellulose acetate filters with a pore size of 0.45 μm.

### Chemical analyses of the sediments

Total organic carbon (TOC), total nitrogen (N), and total sulfur (S), as well as water content (d.w.) and grain-size distribution were determined in six fractions. The chemical parameters comprise selected persistent organic pollutants (POP) such as DDT and its metabolites, HCH-isomers, hexachlorobenzene (HCB), octachlorostyrene (OCS), polychlorinated biphenyls (PCB 28, 52, 101, 118, 138, 153, 180), polycyclic aromatic hydrocarbons (PAH), total petrol hydrocarbons (TPH), tributyltin (TBT) as well as heavy metals and metalloids. TOC and water content were measured according to the German standards DIN 38409-H3 and DIN 38414-S2, respectively. Following microwave assisted digestion with aqua regia at 180°C in closed vessels, heavy metals, and metalloids were determined in the 20 μm fraction by inductively coupled plasma/mass spectrometry, atomic fluorescence spectroscopy (Hg) and hydride atomic absorption spectroscopy (As). To analyze organic contaminants, aliquots were homogenized, freeze-dried, sieved (< 2 mm) and then milled using a planetary mill with vessels of zirconium oxide. The extraction followed German standards (DIN 38414-S20 and 38407-F2). The analyses of chloroorganic compounds were performed by gas chromatography equipped with two ^63^Ni electron-capture detectors and two capillary columns of different polarity. 16 PAHs (according to US EPA 610) were quantified by fluorescence and diode array HPLC (gradient elution) (see DIN 38414-S21). TPH was analyzed according to ISO 16703:2004 and TBT by gas chromatography with flame photometric detection at a wave length of 610 nm after derivatization with sodium tetrabutylborate and extraction with n-hexane.

### Comet assay

Several earlier studies examined the potential of the *in vitro *comet assay using the rainbow trout gonad cell line-2 (RTG-2) or primary hepatocytes and gill cells as an indicator test for genotoxicity assessment of aquatic contaminants and surface waters [42,43] and proved to be generally sensitive and robust enough for the testing of native water samples even without extraction and concentration procedures. The comet assay was based on a common protocol [44] with modifications published previously [43]. Briefly, RTG-II cells (1.5 × 10^6 ^cells) were incubated at 20°C for 120 min with (Eagle's minimal essential medium) E-MEM, sediment pore water and DMSO. After trypsination, mixing E-MEM and low melting agarose, the cells were applied evenly on top of a pre-coated slide. The cell layer was covered with a protective layer of low melting agarose. Subsequently slides were incubated overnight in a lysis solution containing NaCl, EDTA, Tris (pH 10), sodium lauryl sulfate, Triton X-100, and DMSO at 4°C in the dark. To unwind the DNA, slides were immersed in an alkaline buffer (NaOH, EDTA). Electrophoresis was carried out in the same buffer in the dark for 30 min at 25 V and 300 mA. Thereafter, slides were neutralized and the DNA was stained with SYBR Green. For scoring, slides were sealed with a cover slip. From each slide 100 nucleoids (two replicates) were examined at 20× magnification using a fluorescence microscope (Zeiss Imager Z1, Germany) equipped with a monochrome CCD Camera (ProgRes MF cool, Jenoptik, Germany) and an image analysis system (Comet Imager 2.1/Metafer 4, MetaSystems, Germany). For each treatment the tail length, tail moment and % DNA were measured. Significance in DNA damage was assessed by the Dunnet's test at p < 0.05. The proportion of viable RTG-II cells at the end of the 2 h exposure (including negative and positive controls) was determined by a double cytofluorescence assay, which involves simultaneous staining of cells with Fluoresceine diacetat (FDA) and Propidium iodide (PI). After 2 min dyeing, cell viability was measured by flow cytometry (FACS Calibur, BD Biosciences, USA).

### Yeast estrogen screen

This assay utilized *Saccharomyces cerevisiae *strain BF3505 transfected with the receptor plasmid YEPE10 and followed a recently published protocol [45]. The estrogen assay was started by mixing the respective sample with reaction medium (10-fold concentrated growth medium supplemented with CuSO_4_, Ampicillin and Streptomycin) and cell-density-adjusted yeast strain. All samples were tested in six parallels. The loaded microplates were incubated for 18 h at 30°C in a shaking incubator (200 rpm). Subsequently, the cell density was measured at 600 nm. The assay for β-galactosidase was started by adding lacZ-buffer (lysis buffer containing salts, lacZ-substrate ONPG and Lyticase from *Arthrobacter luteus*). After 4 h of incubation at 37°C, enzyme activity was measured at 420 nm according to [46]. Induction ratios were calculated from β-galactosidase units in samples divided by those measured in the negative control, significance was assessed by Tukey's HSD test at p < 0.05.

### Nematode cultivation, synchronization, and exposure for gene expression analysis

*C. elegans *var. Bristol, strain N2, were maintained according to standard procedures [46-49]. To age-synchronize the culture, all individuals were rinsed off the plates and filtered over a 45 μm-gauze using K-medium [50], which retains all but first and second juvenile stage nematodes (L1 and L2). After two days at 20°C, L1 and L2 developed to juveniles of the fourth stage (L4) and young adults. The density of the nematode suspension was adjusted to 40,000 individuals per ml. Five replicates were set up for each of the three sediments treatments, each replicate consisting of 24 sub-replicates using 12-well polystyrene multi dishes (Nunc, Wiesbaden, Germany). Sediment (0.5 g wet weight) and 0.25 ml of bacterial suspension (approximately 10^10 ^*E. coli *cells/ml suspended in K-medium) were mixed and transferred to the appropriate test vials (sub-replicates). Nematode-suspension (0.5 ml, 20,000 individuals) was transferred to each test vial and the test organisms exposed for 48 h to the respective treatment. To recover the nematodes, the content of the vials were transferred into 50 ml centrifuge tubes using a suspension of colloidal silica (Ludox TM50; Sigma-Aldrich, Germany) in deionized water (density: 1.15 g cm^-3^) and centrifuged for 5 min at 800 × *g*. The supernatant, containing the majority of nematodes, was filtered over a 45 μm-gauze to remove small F1 larvae, rinsed back into the falcon tubes and centrifuged for 15 min at 800 × *g*. The harvested worm population therefore consists of staged young adult nematodes. Trizol (Invitrogen, Germany) was added to the nematode pellet and stored at -20°C until further use.

### Sediment toxicity test

The nematode bioassay was carried out, with few modifications, according to a standard method [50] used to draft ISO/DIS 10872 [51]. Sediment (0.5 g wet weight) was mixed with 0.5 ml of *E. coli *(OP50, approximately 10^10 ^cells ml^-1^) suspended in K-medium in 12-well polystyrene multi dishes (Nunc, Wiesbaden, Germany). Five first-stage (L1) juvenile worms were transferred to each test well. Four replicates were set up for each sediment. After 96 h at 20°C, the incubation was terminated by heat-killing the worms at 50°C. The samples were stained with 0.5 ml of aqueous Rose Bengal (0.5 g l-1). Nematodes were isolated from the sediment according to standard procedures (ASTM, 2001), using a mixture of a suspension of colloidal silica (Ludox TM50; Sigma-Aldrich, Germany) and deionized water (density: 1.13 g cm-3). Sediment and nematodes were removed from the test wells with a Pasteur pipette by washing with approximately 5 ml of Ludox. This suspension was transferred to a centrifuge tube (1 ml), thoroughly mixed, and centrifuged for 10 min at 800 × *g*. The supernatant, which contained the nematodes, was poured into a Petri dish, and the pellet, containing sediment particles, was resuspended with diluted Ludox and again centrifuged to extract any remaining nematodes. On average, 84% (± 18% SD; *n *= 68) of the test organisms were recovered by this procedure. Nematode reproduction was quantified by counting the juvenile offspring under a dissecting microscope at 25-fold magnification. The statistic significance was calculated using the one-way ANOVA with post-hoc Dunnett's test (p < 0.01).

### RNA preparation and cDNA synthesis

The RNA isolation followed standard procedures using Trizol reagent (Invitrogen, Germany), however modified to include a homogenization step with 0.5 mm glass beads to maximize cell breakage. RNA was purified subsequently using an RNeasy and DNase digestion kit (Qiagen, Germany) and quantified spectroscopically (NanoDrop1000, ThermoScientific, UK). All experiments were conducted following a reference design with the reference sample generated from a pool of RNA extracted from all sediment exposed worms as well as from the laboratory control. For hybridization, replicate samples (10 μg total RNA) of sediment and a universal reference were reverse transcribed using Moloney Murine Leukemia Virus (MMLV) reverse transcriptase (ABgene, Surrey, UK). The synthesized cDNAs were indirectly labeled with fluorescent dyes, Cy3 and Cy5 (GE Healthcare, UK) and purified using the CyScribe™ GFX Purification Kit (Amersham Biosciences, Buckinghamshire, UK). cDNA labeling quality and quantity was assessed by spectroscopic analysis.

### DNA microarrays

Global transcripts abundance was analyzed using the *C. elegans *oligonucleotide set version 1.1 (Operon™), comprising 19,873 70 mer oligonucleotides designed to the most 3' region of all predicted genes. The oligos were resuspended at 200 ng/μl with Pronto buffer (Corning Inc. Life Sciences UK) and printed onto UltraGAPS™ slides (Corning, Barry, UK) using a Genetix QArray 2 microarraying robot, and immobilized by UV cross linking. Slides were blocked using 1% Bovine Serum Albumin (BSA), 5 × SSC and 0.1% SDS (all Sigma, UK) at 42°C for 45 minutes, washed five times in sterile water and dried. Hybridizations utilized 35 pmol of Cy dye molecules for each channel at 42°C for 24 hours. After removal and washing, slides were scanned at 633 nm (Cy5) and 543 nm (Cy3) on a ScanArray™ Express microarray scanner (Perkin Elmer, Beaconsfield, UK).

### Analysis of microarray data

BlueFuse software (BlueGnome Ltd., Great Shelford, UK) was used to quantify the spot signals. Pre-normalization of raw data was performed via the print-tip Lowess method using the R package (written by Terry Speeds Microarray Data Analysis Group, University of Berkerley, USA). The pre-normalized R & G signal was exported to TIGR Multiexperiment Viewer (MeV) and Genespring GX 7.3.1 (Agilent Technologies, Stockport, UK) for robust statistical data analysis. Initial p-value multiple False Discovery Rate (FDR) correction by Bonferroni, Benjamin-Hochberg, or Westfall-Young Step Down yielded few genes, namely 34 genes (24 up regulated and 10 down regulated) with Rhine and 41 genes (24 up regulated and 17 down regulated) genes with Elbe samples. However, these low numbers are prohibitive for meaningful Gene ontology and Pathway analysis. Rather than "relax" the p-value after ANOVA and FDR correction we opted to limit the data by selecting the 0.7 to 1.4 limits of the normal distribution (equivalent to 71% of the normally distributed data). The data output was analyzed following a per chip and per gene median polishing algorithm and quality assessment of the normalization procedure performed by Boxplot and MA plot visualization. Only data that were present in all 3 sediments and within the 0.7 to 1.4 limits of the normal distribution were used for further analysis. Although this dataset can be expected to contain some false positives (type 1 error), it minimizes the introduction of "false negatives" (type 2 error) without overly minimizing the data set. Particularly in this case it was deemed ideal as we were not seeking to identify individual differentially regulated transcripts (which is when a false positive becomes a problem and Bonferroni/Hochberg type correction valuable – if not essential), but focus on more gene overlapping GO and KEGG pathway analyses.

Principal component analyses (PCA) and condition tree analyses were used to identify the distribution of sample patterns. The cut-off value for up- and down-regulation was set to 1.4-fold, based on previous microarray profiling studies that showed that the magnitude of gene expression differences is frequently below 2-fold [52,53]. Differentially expressed gene lists were created employing one way ANOVA (p < 0.05) without multiple sample correction. Microarray Analysis Tools (developed by James Lund, University of Kentucky, USA) were used to perform functional analyses to identify biological processes and significantly changing gene classes.

### Data accessibility

The transcript profiles reported in this paper have been deposited in the National Center for Biotechnology Information's (NCBI) Gene Expression Omnibus (GEO) database. The accession numbers is GSE11837.

## Authors' contributions

GR, RM, SH and SRS conceived the work. EC and GR provided the river sediments, isolated the pore water and carried out chemical analyses, the Comet assay and the YES test. SH cultivated and exposed *C. elegans *both for reproduction assay and the treatment for gene expression analysis. RM, SCS and SM carried out all DNA microarray experiments including RNA isolation and preparation of cDNA. Analysis and interpretation of microarray data was performed by SCS, RM and SRS. RM, SRS and CEWS wrote most parts of the paper. All authors have read and approved the manuscript.

## Supplementary Material

Additional file 1 **Differentially regulated genes due to the exposure to the Elbe sediment**. Significantly changing transcripts in *C. elegans *exposed to the Elbe (E) sediment [ANOVA, p < 0.05 without multiple sample correction, fold-change to reference sediment Danube (D) > 1.4 (up-regulated) or < 0.7 (down-regulated)]. Click here for file

Additional file 2 **Differentially regulated genes due to the exposure to the Rhine sediment**. Significantly changing transcripts in *C. elegans *exposed to the Rhine (R) sediment [ANOVA, p < 0.05 without multiple sample correction, fold-change to reference sediment Danube (D) > 1.4 (up-regulated) or < 0.7 (down-regulated)]. Click here for file

Additional file 3 **List of all differentially regulated GO categories**. Biological GO processes overrepresented in sediment exposed nematodes using the Danube sediment sample as reference. Click here for file

Additional file 4 **Molecular functions – partial GO tree**. Partial GO tree presenting relevant molecular functions which were found to be overrepresented in *C. elegans *exposed to Elbe and/or Rhine sediments. Click here for file

Additional file 5 **Cellular components – partial GO tree**. Partial GO tree presenting relevant cellular components which were found to be overrepresented in *C. elegans *exposed to Elbe and/or Rhine sediments. Click here for file

Additional file 6 **Overlapping differentially regulated genes of Elbe and Rhine**. *C. elegans *transcripts that significantly changed in response to both Elbe and Rhine sediment exposure [ANOVA, p < 0.05 without multiple sample correction, fold-change to reference sediment Danube > 1.4 (up-regulated) or < 0.7 (down-regulated), for color code see below]. Click here for file

Additional file 7 **Description of 58 differentially expressed genes involved in several reproduction associated, aging regulating and/or developmental processes**. Identification, direction of change and description of all 58 genes which belong to one or more of the selected GO categories. Click here for file

Additional file 8 **Overlapping differentially regulated genes of selected toxico- genomic studies in *C. elegans***. *C. elegans' *transcripts significantly changed in response to sediment exposures (this study) and PCB52 [11], Cd [12] and two humic substances [10]. Click here for file

Additional file 9 **Principal component analysis (PCA) including a liquid medium laboratory control**. PCA of significantly changing genes in *C. elegans *exposed to three river sample sediments: Danube (red), Elbe (yellow) and Rhine (blue) or S-basal, an artificial laboratory control (black). Note that PCA analysis identifies that the laboratory control exposure clusters distinctly separate from the three sediment exposures when laboratory control (**A**) or the Danube sediment (**B**) is used as the baseline dataset. Click here for file
