# Supplementary material for: Gene expression profiling to characterize sediment toxicity – a pilot study using Caenorhabditis elegans whole genome microarrays
Source: BMC Genomics. 2009 Apr 14;10:160. doi: 10.1186/1471-2164-10-160 (PMC2674462; doi:10.1186/1471-2164-10-160)
Supplement: Additional file 1 — Differentially regulated genes due to the exposure to the Elbe sediment. Significantly changing transcripts in C. elegans exposed to the Elbe (E) sediment [ANOVA, p < 0.05 without multiple sample correction, fold-change to reference sediment Danube (D) > 1.4 (up-regulated) or < 0.7 (down-regulated)]. [file 1471-2164-10-160-S1.doc]

### Additional file 1 – Differentially regulated genes due to the exposure to the Elbe sediment

Significantly changing transcripts in *C. elegans* exposed to the Elbe (E) sediment [ANOVA, p<0.05 without multiple sample correction, fold-change to reference sediment Danube (D) > 1.4 (up-regulated) or < 0.7 (down-regulated)].

| **WormBASE ID** | **Elbe** | | **Fold-change over control** | **p -value** | **GCG name** | **Description** |
| --- | --- | --- | --- | --- | --- | --- |
|  | **Mean** | **SE** |  |  |  |  |
| UP-REGULATED GENES | | | | | | |
| F44G4.2 | 12.555 | 1.315 | 3.80 | 0.022 |  | Unnamed protein |
| F19B2.5 | 37.235 | 0.069 | 3.79 | 0.042 |  | Helicase-like transcription factor HLTF/DNA helicase RAD5 |
| C34F11.6 | 25.471 | 0.122 | 3.59 | 0.011 | *msp-49* | Major sperm protein domain |
| T23D8.9a | 2.232 | 0.329 | 3.40 | 0.041 |  | Unnamed protein |
| K10C3.2 | 2.745 | 0.136 | 3.39 | 0.041 |  | Regulator of ATP-sensitive K+ channels Alpha-endosulfine/ARPP-19 |
| Y24D9A.5 | 1.793 | 0.428 | 3.38 | 0.043 |  |  |
| F38E11.3 | 3.772 | 0.22 | 3.34 | 0.045 | *cpi-1* | Atrazine chlorohydrolase/guanine deaminase |
| Y39G10AR.8 | 6.103 | 1.304 | 3.29 | 0.046 |  | Translation initiation factor 2, gamma subunit (eIF-2gamma; GTPase) |
| F57A8.6 | 3.274 | 0.943 | 3.16 | 0.019 |  | Unnamed protein |
| Y116F11B.13 | 3.093 | 0.373 | 3.07 | 0.045 |  |  |
| M04G7.1 | 1.817 | 0.174 | 3.03 | 0.039 |  | Secreted surface protein |
| C51F7.1 | 5.490 | 0.0893 | 3.02 | 0.040 | *frm-7* | Membrane-associated protein tyrosine phosphatase PTP-BAS |
| C44B12.1 | 17.754 | 0.182 | 2.92 | 0.046 |  |  |
| C17G10.9b | 3.630 | 0.514 | 2.89 | 0.049 |  |  |
| ZK622.3a | 2.485 | 0.983 | 2.88 | 0.046 |  |  |
| K06B4.8 | 1.441 | 1.022 | 2.87 | 0.035 |  | Nuclear hormone receptor |
| F44F1.5 | 1.875 | 0.133 | 2.85 | 0.034 |  | Uncharacterized coiled-coil containing protein |
| F28A12.1 | 11.138 | 0.602 | 2.85 | 0.034 |  | Non voltage-gated ion channels (DEG/ENaC family) |
| F55G1.7 | 2.048 | 0.181 | 2.85 | 0.032 |  |  |
| H22D07.1 | 1.739 | 0.306 | 2.83 | 0.040 |  | 7 transmembrane receptor |
| Y71F9B.13b | 2.533 | 0.475 | 2.82 | 0.045 |  |  |
| R06A4.8 | 1.968 | 0.425 | 2.82 | 0.040 |  | Alpha amylase |
| F09F7.6 | 12.396 | 0.172 | 2.76 | 0.004 |  |  |
| F26G5.1 | 1.536 | 0.28 | 2.76 | 0.047 |  |  |
| F21D12.1b | 1.730 | 0.138 | 2.75 | 0.048 | *nhr-21* |  |
| H11E01.1 | 3.815 | 0.18 | 2.75 | 0.039 |  | Predicted transposase |
| K10H10.2 | 4.993 | 0.265 | 2.74 | 0.038 |  | Cystathionine beta-synthase and related enzymes |
| F38E11.6 | 4.783 | 0.523 | 2.73 | 0.034 |  |  |
| F18A1.5 | 3.016 | 0.272 | 2.71 | 0.040 |  | Single-stranded DNA-binding replication protein A (RPA), large (70 kD) subunit |
| Y69H2.11 | 2.015 | 0.658 | 2.70 | 0.043 |  | Non voltage-gated ion channels (DEG/ENaC family) |
| C09F12.2 | 5.427 | 0.374 | 2.65 | 0.049 |  |  |
| F28F8.2 | 3.594 | 0.322 | 2.63 | 0.034 | *acs-2* | Long chain fatty acid acyl-CoA ligase |
| JC8.3a | 20.439 | 0.0599 | 2.62 | 0.040 | *rpl-12* | 40S ribosomal protein S2 |
| H11E01.3 | 1.181 | 0.125 | 2.56 | 0.049 |  |  |
| F36H5.1 | 23.901 | 0.121 | 2.55 | 0.046 |  | Uncharacterized protein, contains BTB/POZ domain |
| C01G10.1 | 2.749 | 0.254 | 2.51 | 0.033 |  | Uncharacterized protein, contains BRCT, WSN domains and ankyrin repeats |
| F42H10.9 | 5.733 | 0.202 | 2.51 | 0.044 |  |  |
| T21H3.5 | 1.423 | 0.111 | 2.51 | 0.036 |  |  |
| Y9C9A.4 | 1.544 | 0.258 | 2.50 | 0.045 | *str-169* |  |
| F48F7.6 | 1.859 | 0.505 | 2.50 | 0.046 |  |  |
| C53B4.5 | 13.574 | 0.135 | 2.49 | 0.048 | *col-119* | Collagens (type IV and type XIII), and related proteins |
| T16H12.10 | 2.135 | 0.196 | 2.48 | 0.016 |  | No Significant Match |
| Y39A3CL.4b | 2.618 | 0.652 | 2.48 | 0.045 |  | Uncharacterized conserved protein with TLDc domain |
| T19B4.1 | 7.404 | 0.0966 | 2.47 | 0.036 |  | Peptidylglycine alpha-amidating monooxygenase |
| C50F4.1 | 2.226 | 0.0744 | 2.47 | 0.045 |  |  |
| Y116A8C.9 | 1.665 | 0.229 | 2.47 | 0.047 |  | Predicted membrane protein |
| C07B5.3 | 8.374 | 0.0656 | 2.46 | 0.001 |  | Unnamed protein |
| C37A2.7 | 30.642 | 0.236 | 2.46 | 0.049 |  |  |
| T24C2.2 | 1.262 | 0.219 | 2.46 | 0.034 |  | Unnamed protein |

| C53B7.3 | 9.655 | 0.331 | 2.45 | 0.032 |  |  |
| --- | --- | --- | --- | --- | --- | --- |
| F48A11.5 | 3.995 | 0.434 | 2.45 | 0.034 |  |  |
| F55G1.6 | 7.966 | 0.284 | 2.45 | 0.032 |  | Unnamed protein |
| F37E3.3 | 878,8 | 0.444 | 2.44 | 0.034 |  |  |
| T12D8.8 | 7.650 | 0.288 | 2.43 | 0.038 |  | Hsp70-interacting protein Hip/component of progesterone receptor complexes |
| R05D8.5 | 5.031 | 0.416 | 2.40 | 0.044 | *srx-28* | 7-transmembrane receptor |
| K06H6.4 | 1.647 | 0.273 | 2.40 | 0.035 |  | Extracellular protein with conserved cysteines |
| F36H12.8 | 2.076 | 0.187 | 2.39 | 0.034 |  | Casein kinase (serine/threonine/tyrosine protein kinase) |
| T12G3.1 | 8.382 | 0.233 | 2.39 | 0.031 |  | Uncharacterized conserved protein, contains ZZ-type Zn-finger |
| C35C5.5 | 1.445 | 0.622 | 2.39 | 0.044 | *lev-8* | Acetylcholine receptor |
| Y59A8B.9 | 2.553 | 0.202 | 2.39 | 0.045 |  | Microtubule-binding protein involved in cell cycle control |
| C01F1.1 | 4.846 | 0.363 | 2.39 | 0.031 |  | Transcription initiation factor IIF, large subunit (RAP74) |
| F17H10.1 | 1.496 | 0.212 | 2.38 | 0.012 |  | Uncharacterized conserved protein |
| M88.2 | 10.310 | 0.309 | 2.38 | 0.043 |  | Mitochondrial ribosomal protein S34 |
| F48F7.5 | 1.609 | 0.176 | 2.36 | 0.025 |  | Unnamed protein |
| ZK1098.5 | 2.942 | 0.424 | 2.36 | 0.044 |  | Transport protein particle (TRAPP) complex subunit |
| F30B5.4 | 2.950 | 0.606 | 2.35 | 0.047 |  | Unnamed protein |
| C54G7.3b | 1.210 | 0.156 | 2.34 | 0.048 | *lgx-1* |  |
| T26A5.9 | 8.656 | 0.131 | 2.33 | 0.041 | *dlc-1* | Dynein light chain type 1 |
| B0414.3 | 7.766 | 0.225 | 2.33 | 0.032 | *hil-5* | Histone H1 |
| Y38F2AR.1 | 2.876 | 0.422 | 2.33 | 0.043 |  | Unnamed protein |
| C18B2.5a | 1.552 | 0.117 | 2.32 | 0.048 |  |  |
| R11A5.3 | 4.517 | 0.215 | 2.32 | 0.036 |  |  |
| R08A2.3 | 3.604 | 0.24 | 2.32 | 0.044 |  |  |
| K09A9.3 | 3.969 | 0.171 | 2.32 | 0.035 | *ent-2* | Nucleoside transporter |
| F43D9.4 | 41.016 | 0.203 | 2.30 | 0.032 | *sip-1* | Alpha crystallins |
| T05H4.4 | 1.821 | 0.158 | 2.28 | 0.036 |  | NADH-cytochrome b-5 reductase |
| C53A5.3 | 2.127 | 0.231 | 2.27 | 0.033 | *hda-1* | Histone deacetylase complex, catalytic component RPD3 |
| C25E10.7 | 29.973 | 0.299 | 2.26 | 0.032 |  | Uncharacterized protein |
| C52E12.1 | 1.463 | 0.105 | 2.25 | 0.050 |  | Predicted E3 ubiquitin ligase |
| B0513.4 | 4.741 | 0.104 | 2.24 | 0.037 |  |  |
| F11A6.1a | 5.461 | 0.464 | 2.22 | 0.039 | *kpc-1* | Subtilisin-like proprotein convertase |
| C16A11.4 | 2.480 | 0.133 | 2.21 | 0.041 |  | Uncharacterized protein |
| F54B8.2 | 1.239 | 0.395 | 2.20 | 0.038 |  | Nuclear hormone receptor |
| K08C9.2 | 5.122 | 0.291 | 2.20 | 0.040 |  | Unnamed protein |
| F25A2.1 | 6.022 | 0.125 | 2.20 | 0.034 |  | Predicted lipase |
| C09G5.8 | 1.316 | 0.341 | 2.19 | 0.045 |  | Unnamed protein |
| D1037.3 | 10.182 | 0.288 | 2.19 | 0.042 | *ftn-2* | Ferritin |
| B0361.10 | 1.803 | 0.451 | 2.18 | 0.042 |  | SNARE protein YKT6, synaptobrevin/VAMP syperfamily |
| C56C10.8 | 22.740 | 0.337 | 2.18 | 0.023 | *icd-1* | RNA polymerase II general transcription factor BTF3 and related proteins |
| Y51H7C.10 | 36.297 | 0.102 | 2.18 | 0.045 |  |  |
| C44E4.4 | 2.801 | 0.092 | 2.17 | 0.033 |  | RNA-binding protein La |
| F37C12.4 | 38.407 | 0.133 | 2.17 | 0.049 | *rpl-36* | 60S ribosomal protein L36 |
| K04F1.10 | 1.983 | 0.0865 | 2.17 | 0.041 |  | Predicted receptor |
| H13N06.3b | 2.015 | 0.346 | 2.17 | 0.048 | *gob-1* | Unnamed protein |
| F54D1.2 | 2.209 | 0.285 | 2.15 | 0.042 | *col-127* | Collagens (type IV and type XIII), and related proteins |
| F02E9.2a | 2.425 | 0.237 | 2.15 | 0.044 | *lin-28* | Predicted RNA-binding protein containing PIN domain |
| F01E11.2 | 2.287 | 0.162 | 2.15 | 0.001 |  |  |
| C05D12.1 | 1.065 | 0.178 | 2.14 | 0.009 |  | Predicted membrane protein |
| F26A1.3 | 1.586 | 0.593 | 2.14 | 0.032 |  | Casein kinase (serine/threonine/tyrosine protein kinase) |
| Y55F3BL.1 | 1.231 | 0.181 | 2.14 | 0.044 |  | Mitochondrial ribosomal protein L17 |
| R11G11.7 | 1.742 | 0.186 | 2.13 | 0.036 | *pqn-60* | Predicted alpha-helical protein |
| C03A7.7 | 6.266 | 0.132 | 2.13 | 0.033 | *abu-6* | Uncharacterized protein |
| T06E6.3 | 1.111 | 0.22 | 2.13 | 0.036 | *srx-40* | 7-transmembrane receptor |
| K11D9.1b | 2.843 | 0.142 | 2.13 | 0.048 | *klp-7* | Kinesin-like protein |
| W03D2.4 | 7.570 | 0.269 | 2.12 | 0.037 | *pcn-1* | DNA polymerase delta processivity factor |
| R01E6.1 | 2.270 | 0.134 | 2.12 | 0.046 | *odr-1* | Natriuretic peptide receptor, guanylate cyclase |
| C27D6.9 | 1.393 | 0.328 | 2.12 | 0.032 | *srb-2* |  |

| F08F8.8 | 1.018 | 0.286 | 2.12 | 0.046 |  | SNARE protein GS28 |
| --- | --- | --- | --- | --- | --- | --- |
| T28F4.5 | 18.978 | 0.128 | 2.12 | 0.031 |  | Unnamed protein |
| C16D9.8 | 4.468 | 0.468 | 2.12 | 0.032 |  |  |
| T01C3.8 | 1.988 | 0.152 | 2.12 | 0.044 |  |  |
| Y66A7A.4 | 4.264 | 0.466 | 2.12 | 0.042 |  | Unnamed protein |
| T25E12.4b | 1.665 | 0.241 | 2.11 | 0.040 |  |  |
| ZK973.4 | 2.164 | 0.391 | 2.11 | 0.044 |  | VAMP-associated protein involved in inositol metabolism |
| T23B7.1 | 11.409 | 0.435 | 2.11 | 0.015 |  | Confirmed |
| F35A5.4 | 7.542 | 0.0993 | 2.11 | 0.026 |  | Uncharacterized protein |
| F09C12.7 | 18.977 | 0.271 | 2.10 | 0.009 | *msp-74* |  |
| M05D6.5 | 1.892 | 0.0835 | 2.10 | 0.019 |  | Uncharacterized protein, induced by hypoxia |
| Y116F11B.3 | 2.560 | 0.159 | 2.09 | 0.045 | *pcp-4* | Hydrolytic enzymes of the alpha/beta hydrolase fold |
| F57B10.8 | 4.724 | 0.0511 | 2.09 | 0.035 |  | TBP-binding protein, activator of basal transcription |
| B0457.3 | 1.025 | 0.123 | 2.08 | 0.000 |  |  |
| K05C4.8 | 13.301 | 0.192 | 2.08 | 0.041 |  | Unnamed protein |
| B0238.5 | 1.449 | 0.321 | 2.07 | 0.032 | *srt-67* | Integral membrane O-acyltransferase |
| C51F7.2 | 1.114 | 0.289 | 2.07 | 0.040 | *srg-45* | Receptor-like protein, Srg family |
| F43E2.1 | 2.043 | 0.184 | 2.06 | 0.050 |  | S-M checkpoint control protein CID1 |
| F48A9.2 | 3.201 | 0.246 | 2.06 | 0.034 |  |  |
| C06B3.9 | 1.747 | 0.255 | 2.06 | 0.037 | *str-252* | 7-transmembrane olfactory receptor |
| ZK105.2 | 1.699 | 0.412 | 2.06 | 0.043 |  |  |
| F56C9.5 | 1.347 | 0.261 | 2.06 | 0.003 | *acbp-4* | Acyl-CoA-binding protein |
| T05F1.3 | 33.293 | 0.181 | 2.06 | 0.036 | *rps-19* | 40S ribosomal protein S19 |
| F09E5.7 | 2.776 | 0.456 | 2.06 | 0.047 |  |  |
| C24D10.7 | 9.513 | 0.208 | 2.05 | 0.039 | *nlp-9* | Unnamed protein |
| R09B3.3 | 28.993 | 0.0805 | 2.05 | 0.038 |  | mRNA cleavage and polyadenylation factor I complex |
| K02A11.2 | 1.481 | 0.228 | 2.04 | 0.046 |  | Unnamed protein |
| C24H12.4a | 2.309 | 0.0723 | 2.04 | 0.046 |  | RNA helicase |
| C24B9.3 | 1.888 | 0.043 | 2.04 | 0.048 |  |  |
| F58G6.4 | 3.153 | 0.102 | 2.04 | 0.047 |  | Ligand-gated ion channel |
| C03A7.14 | 2.711 | 0.219 | 2.03 | 0.033 | *abu-8* | Uncharacterized protein |
| F14D7.7 | 2.071 | 0.252 | 2.03 | 0.034 |  | Uncharacterized protein |
| C36E8.5 | 14.358 | 0.275 | 2.02 | 0.014 | *tbb-2* | Beta tubulin |
| Y116A8A.2 | 1.846 | 0.271 | 2.01 | 0.043 |  |  |
| R07A4.4 | 2.266 | 0.185 | 2.01 | 0.028 |  | Unnamed protein |
| F46A9.4 | 5.560 | 0.276 | 2.01 | 0.034 | *skr-2* | SCF ubiquitin ligase, Skp1 component |
| C10A4.2 | 11.846 | 0.0701 | 2.01 | 0.005 |  |  |
| C07G1.2 | 1.655 | 0.171 | 2.00 | 0.031 |  |  |
| F22B7.9 | 1.971 | 0.139 | 2.00 | 0.048 |  | Predicted methyltransferase |
| F35F10.8 | 2.064 | 0.224 | 2.00 | 0.034 |  | No Significant Match |
| H12I19.4 | 2.601 | 0.127 | 2.00 | 0.038 |  | Integral membrane O-acyltransferase |
| H10D12.2 | 2.481 | 0.0959 | 2.00 | 0.039 |  |  |
| C50B6.4 | 1.568 | 0.12 | 2.00 | 0.033 | *col-161* | Collagens (type IV and type XIII), and related proteins |
| F14D7.4 | 1.044 | 0.237 | 2.00 | 0.034 |  |  |
| ZK1193.1 | 11.229 | 0.28 | 1.99 | 0.021 | *col-19* | Collagens (type IV and type XIII), and related proteins |
| F56C3.6 | 1.872 | 0.191 | 1.99 | 0.039 | *dgn-2* | Dystroglycan |
| EEED8.15 | 1.721 | 0.223 | 1.99 | 0.045 |  |  |
| H39E20.1 | 6.267 | 0.0833 | 1.98 | 0.041 |  |  |
| M01G12.6 | 4.144 | 0.304 | 1.98 | 0.040 |  | 7-transmembrane olfactory receptor |
| F07E5.1 | 2.921 | 0.125 | 1.98 | 0.034 |  | Predicted transposase |
| K09E3.5 | 6.635 | 0.181 | 1.98 | 0.026 |  | Uncharacterized protein |
| C55B6.3 | 1.226 | 0.195 | 1.97 | 0.033 |  | Predicted unusual protein kinase |
| M18.1 | 2.249 | 0.183 | 1.97 | 0.013 | *col-129* | Collagens (type IV and type XIII), and related proteins |
| F59F4.2 | 13.589 | 0.127 | 1.97 | 0.035 |  | Predicted membrane protein |
| Y41C4A.4e | 2.157 | 0.0542 | 1.97 | 0.050 | *crh-1* |  |
| C32F10.4 | 12.843 | 0.376 | 1.97 | 0.033 |  |  |
| W09H1.1 | 2.326 | 0.184 | 1.97 | 0.042 |  |  |
| F59A1.11 | 3.545 | 0.457 | 1.97 | 0.040 |  | No Significant Match |

| Y106G6H.3 | 24.559 | 0.143 | 1.96 | 0.042 | *rpl-30* | 60S ribosomal protein L30 |
| --- | --- | --- | --- | --- | --- | --- |
| Y73B6BL.29 | 2.039 | 0.147 | 1.96 | 0.047 |  | Predicted pseudouridylate synthase |
| F40F8.1 | 2.212 | 0.12 | 1.96 | 0.032 |  | Uridylate kinase/adenylate kinase |
| K08C7.2 | 1.303 | 0.216 | 1.96 | 0.050 | *fmo-1* | Flavin-containing monooxygenase |
| F54A3.5 | 9.583 | 0.122 | 1.95 | 0.045 |  | Uncharacterized conserved protein |
| T05H4.1 | 1.725 | 0.135 | 1.95 | 0.036 | *acl-8* | Lysophosphatidic acid acyltransferase LPAAT |
| Y54G9A.7 | 2.485 | 0.303 | 1.94 | 0.045 |  |  |
| R02F2.5 | 3.900 | 0.186 | 1.94 | 0.006 |  | Partially_confirmed TR:Q21648 AAA50721.1 |
| E03D2.2a | 2.718 | 0.261 | 1.93 | 0.039 | *nlp-9* | Unnamed protein |
| F37D6.3 | 2.293 | 0.113 | 1.93 | 0.020 |  |  |
| W01A11.1 | 1.558 | 0.145 | 1.93 | 0.037 |  | Predicted hydrolases or acyltransferases |
| C36A4.9 | 3.320 | 0.174 | 1.92 | 0.045 |  |  |
| K01A2.5 | 1.494 | 0.0716 | 1.92 | 0.041 |  | Predicted hydrolase |
| K07A1.11 | 2.294 | 0.25 | 1.92 | 0.035 | *rba-1* | Nucleosome remodeling factor, subunit CAF1/NURF55/MSI1 |
| F29G6.3b | 8.146 | 0.154 | 1.92 | 0.040 |  | Unnamed protein |
| T01C4.1 | 4.589 | 0.0832 | 1.92 | 0.036 |  | Chitinase |
| Y51H7C.13 | 2.296 | 0.253 | 1.92 | 0.043 |  | Unnamed protein |
| T03F7.5 | 4.519 | 0.36 | 1.92 | 0.031 |  | Unnamed protein |
| W03D2.7 | 1.827 | 0.13 | 1.92 | 0.047 |  |  |
| F59A6.2 | 2.826 | 0.202 | 1.91 | 0.009 |  |  |
| C44B7.10 | 3.590 | 0.217 | 1.91 | 0.050 |  | Acetyl-CoA hydrolase |
| C25A8.4 | 2.875 | 0.123 | 1.90 | 0.050 |  | Chitinase |
| C16C4.7 | 1.240 | 0.312 | 1.90 | 0.033 |  |  |
| C16C10.11 | 3.314 | 0.125 | 1.89 | 0.006 |  | Uncharacterized conserved protein |
| F56H1.5 | 1.246 | 0.41 | 1.89 | 0.039 |  | Zinc carboxypeptidase |
| ZK1321.1 | 3.075 | 0.15 | 1.89 | 0.010 |  |  |
| F58F6.2 | 4.020 | 0.149 | 1.88 | 0.039 | *col-105* | Collagens (type IV and type XIII), and related proteins |
| T10H9.4 | 5.665 | 0.136 | 1.88 | 0.040 | *snb-1* | Synaptobrevin/VAMP-like protein |
| H02I12.1 | 7.315 | 0.243 | 1.88 | 0.038 |  | Unnamed protein |
| Y17G7B.8 | 1.755 | 0.097 | 1.87 | 0.041 |  | Uncharacterized protein |
| F53A9.8 | 39.230 | 0.0823 | 1.87 | 0.042 |  | Confirmed TR:Q20693 AAC46563.1 |
| F21C10.8 | 1.388 | 0.147 | 1.87 | 0.032 | *pqn-31* |  |
| F59E12.3 | 1.663 | 0.13 | 1.87 | 0.039 |  | No Significant Match |
| F53C11.6 | 1.015 | 0.117 | 1.86 | 0.035 | *twk-32* | Tandem pore domain K+ channel |
| H06I04.2 | 1.037 | 0.102 | 1.86 | 0.043 | *sft-1* | Mitochondrial protein Surfeit 1/SURF1/SHY1 |
| W02D7.9 | 853 | 0.134 | 1.86 | 0.037 |  |  |
| B0336.6 | 2.524 | 0.345 | 1.86 | 0.048 |  | Abl interactor ABI-1, contains SH3 domain |
| C17C3.2 | 11.186 | 0.442 | 1.86 | 0.007 |  | Unnamed protein |
| C06H2.1 | 13.590 | 0.154 | 1.86 | 0.037 |  | Mitochondrial F1F0-ATP synthase, subunit d/ATP7 |
| K02B12.8 | 9.326 | 0.359 | 1.85 | 0.046 | *zhp-3* | Uncharacterized protein involved in synaptonemal complex formation |
| K02B2.5 | 51.984 | 0.0718 | 1.85 | 0.045 | *rps-25* | 40S ribosomal protein S25 |
| M03F4.7 | 2.418 | 0.11 | 1.85 | 0.036 |  | Peroxidase/oxygenase |
| Y39B6A.11 | 1.481 | 0.289 | 1.85 | 0.043 |  |  |
| R05G9.3 | 3.062 | 0.171 | 1.85 | 0.042 |  |  |
| K03E5.2 | 1.822 | 0.201 | 1.84 | 0.040 |  |  |
| F58A3.4 | 2.588 | 0.182 | 1.84 | 0.035 |  | Uncharacterized protein, contains major sperm protein (MSP) domain |
| C24B9.9 | 2.383 | 0.151 | 1.84 | 0.039 | *dod-3* | Unnamed protein |
| Y46E12A.3 | 1.943 | 0.12 | 1.84 | 0.047 |  | Glutaredoxin-related protein |
| F41G3.5 | 2.236 | 0.254 | 1.84 | 0.038 |  | Casein kinase (serine/threonine/tyrosine protein kinase) |
| F58B3.6 | 3.431 | 0.289 | 1.84 | 0.037 |  | Interferon-related protein PC4 like |
| D2013.8 | 4.437 | 0.191 | 1.84 | 0.050 | *scp-1* |  |
| F08F3.8 | 2.123 | 0.164 | 1.83 | 0.034 |  | Protein tyrosine phosphatase, contains WSN domain |
| T26E4.7 | 2.002 | 0.231 | 1.83 | 0.038 |  | Glycosyltranferase |
| C45G7.3 | 9.391 | 0.216 | 1.83 | 0.044 |  | Unnamed protein |
| W02D9.10 | 1.851 | 0.151 | 1.83 | 0.037 |  | Unnamed protein |
| C54G6.2 | 1.876 | 0.0585 | 1.83 | 0.050 |  | Uncharacterized conserved protein |
| Y102A5C.33 | 1.400 | 0.194 | 1.82 | 0.042 | *srz-95* | 7-transmembrane receptor |
| C25A1.8 | 7.239 | 0.215 | 1.82 | 0.033 |  | C-type lectin |

| C47A10.3 | 6.185 | 0.227 | 1.82 | 0.037 |  | No Significant Match |
| --- | --- | --- | --- | --- | --- | --- |
| F35A5.1 | 4.420 | 0.19 | 1.81 | 0.014 |  | Unnamed protein |
| C50E3.6 | 937,7 | 0.0787 | 1.81 | 0.048 |  |  |
| F47B7.1 | 3.852 | 0.299 | 1.81 | 0.025 |  | Stress responsive protein |
| F45D3.2 | 2.759 | 0.0744 | 1.81 | 0.034 |  |  |
| F42C5.2 | 5.281 | 0.128 | 1.81 | 0.037 |  | 7 transmembrane receptor |
| C39B5.5 | 6.381 | 0.271 | 1.81 | 0.042 |  | Predicted lipase |
| F33H2.2 | 3.022 | 0.187 | 1.81 | 0.039 |  | Uncharacterized conserved protein |
| T05D4.1 | 1.776 | 0.267 | 1.81 | 0.038 |  | Fructose-biphosphate aldolase |
| B0280.9 | 1.673 | 0.166 | 1.80 | 0.034 |  | WD40 repeat protein |
| T28H11.6 | 4.232 | 0.397 | 1.80 | 0.033 | *ssp-11* | Uncharacterized protein, contains major sperm protein (MSP) domain |
| C32D5.10 | 1.445 | 0.119 | 1.80 | 0.048 |  | Predicted E3 ubiquitin ligase |
| C47E12.3 | 2.321 | 0.159 | 1.80 | 0.017 |  | Glycosyl hydrolase, family 47 |
| R07B5.7 | 2.416 | 0.0995 | 1.80 | 0.033 |  |  |
| F26F12.3b | 10.746 | 0.214 | 1.80 | 0.042 |  |  |
| F36A2.7 | 6.853 | 0.215 | 1.80 | 0.034 |  | Unnamed protein |
| F07H5.6 | 19.435 | 0.192 | 1.79 | 0.042 |  | Predicted TR:Q19176 CAA92658.1 |
| F32B6.9 | 20.234 | 0.278 | 1.79 | 0.034 |  | Bestrophin (Best vitelliform macular dystrophy-associated protein) |
| F28B3.10 | 4.283 | 0.128 | 1.79 | 0.047 |  | Unnamed protein |
| W05F2.3 | 7.227 | 0.26 | 1.79 | 0.037 |  |  |
| T02G5.9a | 2.299 | 0.149 | 1.79 | 0.009 | *krs-1* |  |
| C18E9.9 | 2.976 | 0.206 | 1.79 | 0.042 |  |  |
| F55F8.5 | 1.931 | 0.0588 | 1.78 | 0.035 | *tag-345* | Microtubule binding protein YTM1 (contains WD40 repeats) |
| C24H12.2 | 6.419 | 0.147 | 1.78 | 0.033 |  | No Significant Match |
| T01C8.3 | 1.910 | 0.156 | 1.78 | 0.050 |  | Unnamed protein |
| F54D11.1 | 4.754 | 0.169 | 1.78 | 0.035 |  | SAM-dependent methyltransferases |
| F57B10.1 | 2.091 | 0.131 | 1.78 | 0.035 |  | CREB/ATF family transcription factor |
| Y50D4A.2 | 3.582 | 0.166 | 1.77 | 0.049 |  | Tryptophan-rich basic nuclear protein |
| F26E4.6 | 17.140 | 0.0924 | 1.77 | 0.049 |  | Cytochrome c oxidase, subunit VIIc/COX8 |
| C23G10.11 | 2.248 | 0.12 | 1.77 | 0.040 |  |  |
| T05F1.6a | 42.306 | 0.125 | 1.76 | 0.041 | *hsr-9* | DNA damage checkpoint protein RHP9/CRB2/53BP1 |
| T13H5.1 | 3.097 | 0.121 | 1.76 | 0.004 |  |  |
| Y24D9A.1b | 2.310 | 0.159 | 1.76 | 0.048 |  |  |
| C04G6.1b | 980 | 0.277 | 1.76 | 0.050 | *mpk-2* |  |
| F15A4.2 | 1.042 | 0.159 | 1.76 | 0.037 |  | Unnamed protein |
| C18D11.1 | 1.279 | 0.288 | 1.76 | 0.040 |  |  |
| C36E8.4 | 1.328 | 0.199 | 1.75 | 0.049 |  | Uncharacterized conserved protein |
| ZK593.6 | 5.908 | 0.0898 | 1.75 | 0.031 | *lgg-2* | Microtubule-associated anchor protein;autophagy and membrane trafficking |
| ZK858.5 | 3.374 | 0.145 | 1.75 | 0.037 |  |  |
| Y46G5A.17 | 3.699 | 0.299 | 1.74 | 0.047 |  | Carnitine O-acyltransferase CPTI |
| ZK816.5 | 15.177 | 0.153 | 1.74 | 0.007 | *dhs-26* | Reductases with broad range of substrate specificities |
| T01C3.3 | 3.571 | 0.2 | 1.74 | 0.036 |  | Predicted E3 ubiquitin ligase |
| ZK938.5 | 1.101 | 0.168 | 1.74 | 0.044 | *old-2* | Fibroblast/platelet-derived growth factor receptor |
| E04A4.8 | 24.267 | 0.15 | 1.74 | 0.043 | *rpl-20* | 60S ribosomal protein L18A |
| T04A8.3 | 2.255 | 0.194 | 1.74 | 0.039 |  | Lectin C-type domain/CUB domain |
| B0495.10c | 1.198 | 0.192 | 1.74 | 0.050 |  |  |
| D2021.8 | 6.373 | 0.265 | 1.73 | 0.012 |  | Ankyrin repeat and DHHC-type Zn-finger domain containing proteins |
| H12I19.1 | 3.415 | 0.258 | 1.73 | 0.038 |  | No Significant Match |
| C18C4.10c | 2.158 | 0.0939 | 1.73 | 0.051 | *klc-2* |  |
| Y51H7BR.6 | 1.536 | 0.0729 | 1.73 | 0.044 |  | No Significant Match |
| ZK1248.1 | 1.388 | 0.172 | 1.73 | 0.041 |  | M13 family peptidase |
| F44E2.6 | 1.789 | 0.13 | 1.72 | 0.014 |  | Predicted pilin-like transcription factor |
| F54C9.4 | 1.777 | 0.0741 | 1.72 | 0.042 | *col-38* | Collagens (type IV and type XIII), and related proteins |
| C31E10.5 | 1.683 | 0.114 | 1.72 | 0.033 |  | Uncharacterized conserved protein |
| F53H4.5 | 5.953 | 0.153 | 1.72 | 0.035 |  | Nuclear DEAF-1 related transcriptional regulator (suppressin) |
| R03E9.3 | 2.613 | 0.0693 | 1.72 | 0.047 | *abts-4* | Na+-independent Cl/HCO3 exchanger AE1 |
| K04G7.11 | 2.591 | 0.124 | 1.72 | 0.049 |  | Cyclin D-interacting protein GCIP |
| B0302.2 | 982,9 | 0.158 | 1.72 | 0.030 |  |  |

| K04F10.4c | 1.540 | 0.173 | 1.71 | 0.035 | *bli-4* | Subtilisin-like proprotein convertase |
| --- | --- | --- | --- | --- | --- | --- |
| T22A3.4a | 42.355 | 0.17 | 1.71 | 0.037 |  |  |
| F18A11.3 | 1.026 | 0.168 | 1.71 | 0.034 |  | Confirmed TR:O01323 CAB04134.1 |
| Y39B6A.25 | 2.811 | 0.268 | 1.71 | 0.048 |  | Unnamed protein |
| C47B2.2b | 38.003 | 0.158 | 1.71 | 0.043 |  | Predicted uracil phosphoribosyltransferase |
| Y75B8A.14 | 1.963 | 0.208 | 1.71 | 0.043 |  | Putative transcription factor FET5 |
| K07A12.6 | 641,7 | 0.121 | 1.71 | 0.035 | *hot-5* | Unnamed protein |
| R06F6.2 | 2.567 | 0.164 | 1.70 | 0.034 |  | Vacuolar assembly/sorting protein PEP5/VPS11 |
| Y50D7A.5 | 1.031 | 0.0953 | 1.70 | 0.045 |  |  |
| C53D5.3 | 1.968 | 0.291 | 1.70 | 0.042 |  |  |
| R04B5.8 | 3.150 | 0.143 | 1.70 | 0.002 | *srd-49* | Chemoreceptor/7TM receptor |
| B0047.3 | 837,8 | 0.0495 | 1.70 | 0.032 |  | Uncharacterized protein, contains BTB/POZ domain |
| K08A2.2 | 1.157 | 0.0594 | 1.70 | 0.042 |  | Uncharacterized protein |
| F26F4.10 | 1.836 | 0.0857 | 1.70 | 0.048 | *rrt-1* | Arginyl-tRNA synthetase |
| F14H12.2 | 2.344 | 0.141 | 1.69 | 0.032 |  | Unnamed protein |
| F20D12.2 | 1.674 | 0.0739 | 1.69 | 0.020 |  | Nuclear protein export factor |
| B0213.2 | 8.533 | 0.204 | 1.68 | 0.038 |  | Confirmed |
| R107.6 | 2.565 | 0.225 | 1.68 | 0.034 | *cls-2* | CLIP-associating protein |
| R08F11.7 | 1.780 | 0.229 | 1.68 | 0.036 | *cnc-4* | Unnamed protein |
| T15B7.8 | 16.443 | 0.101 | 1.68 | 0.040 |  | Uncharacterized conserved protein |
| B0303.9 | 5.828 | 0.086 | 1.68 | 0.011 |  | Vacuolar sorting protein VPS33/slp1 (Sec1 family) |
| M01G12.13 | 9.010 | 0.178 | 1.68 | 0.040 | *sri-13* | Predicted olfactory G-protein coupled receptor |
| T08B6.5 | 8.680 | 0.177 | 1.68 | 0.039 |  |  |
| F08D12.9 | 705,5 | 0.204 | 1.67 | 0.034 |  | Predicted transposase |
| C30G4.4 | 1.684 | 0.166 | 1.67 | 0.050 |  |  |
| T28A11.2 | 2.208 | 0.11 | 1.67 | 0.042 |  | Predicted secreted cysteine rich protein found only in C.elegans |
| Y69A2AR.21 | 1.596 | 0.152 | 1.67 | 0.046 |  | Uncharacterized conserved protein |
| Y105C5B.18 | 4.788 | 0.0639 | 1.67 | 0.050 |  | Unnamed protein |
| T24H7.5b | 4.939 | 0.177 | 1.67 | 0.042 | *tat-4* | P-type ATPase |
| C50B8.2 | 1.482 | 0.111 | 1.67 | 0.033 | *bir-2* | Apoptosis inhibitor IAP1 and related BIR domain proteins |
| F15D3.5 | 7.405 | 0.236 | 1.67 | 0.037 |  |  |
| F13G3.4 | 2.231 | 0.106 | 1.67 | 0.039 | *dylt-1* | Dynein light chain |
| F57G8.2 | 1.752 | 0.149 | 1.66 | 0.041 | *srv-36* | Uncharacterized protein |
| D2096.10 | 1.752 | 0.113 | 1.66 | 0.046 |  | Unnamed protein |
| F55A11.3 | 13.260 | 0.0787 | 1.66 | 0.034 |  | E3 ubiquitin ligase |
| F26D10.12 | 1.260 | 0.165 | 1.66 | 0.041 |  | C-type lectin |
| T05E11.3 | 7.102 | 0.178 | 1.66 | 0.031 |  | Endoplasmic reticulum glucose-regulated protein |
| Y53F4B.39 | 1.166 | 0.0922 | 1.66 | 0.044 |  | Glyoxylase |
| T01C4.4 | 11.512 | 0.222 | 1.65 | 0.036 | *srx-34* | 7-transmembrane receptor |
| Y71A12B.6 | 2.192 | 0.156 | 1.65 | 0.045 |  | C-type lectin |
| F30F8.3 | 1.160 | 0.0919 | 1.65 | 0.046 |  | PDZ domain |
| ZK1067.6 | 1.121 | 0.184 | 1.65 | 0.031 |  | RNA-binding protein Fusilli, contains RRM domain |
| ZK867.1b | 2.725 | 0.0928 | 1.65 | 0.047 |  | Zn-finger |
| R03E9.1 | 6.293 | 0.218 | 1.65 | 0.009 | *mdl-1* | Upstream transcription factor 2/L-myc-2 protein |
| C25F9.6 | 1.279 | 0.0935 | 1.65 | 0.039 |  |  |
| M01E11.2 | 1.666 | 0.201 | 1.64 | 0.036 |  |  |
| Y92H12BR.2 | 1.494 | 0.134 | 1.64 | 0.045 |  |  |
| K01A2.2 | 5.349 | 0.154 | 1.64 | 0.041 | *far-7* |  |
| F07C3.9 | 1.936 | 0.104 | 1.64 | 0.032 |  |  |
| R10D12.12 | 1.721 | 0.11 | 1.64 | 0.044 |  | Predicted glycosyltransferase |
| F53C11.8 | 1.279 | 0.114 | 1.64 | 0.035 |  | Conserved WD40 repeat-containing protein AN11 |
| Y46D2A.2 | 1.597 | 0.19 | 1.64 | 0.044 |  |  |
| T26F2.1 | 2.686 | 0.26 | 1.63 | 0.036 |  | Predicted chitinase |
| C44B12.2 | 7.490 | 0.258 | 1.63 | 0.033 | *ost-1* | Matricellular protein Osteonectin/SPARC/BM-40 |
| Y41C4A.1 | 1.051 | 0.169 | 1.63 | 0.050 |  | Splicing factor RNPS1, SR protein superfamily |
| C04F12.9 | 1.486 | 0.116 | 1.63 | 0.041 |  | Ribonuclease H |
| C10G8.3 | 3.805 | 0.194 | 1.63 | 0.042 |  | Serine proteinase inhibitor (KU family) |
| C14B9.7 | 27.097 | 0.171 | 1.63 | 0.034 | *rpl-21* | 60S ribosomal protein L21 |

| F14B6.3 | 1.373 | 0.12 | 1.63 | 0.039 |  | Unnamed protein |
| --- | --- | --- | --- | --- | --- | --- |
| F35E2.1 | 2.398 | 0.168 | 1.63 | 0.039 |  | Unnamed protein |
| T01G9.2a | 3.886 | 0.0633 | 1.63 | 0.046 |  | Uncharacterized conserved protein |
| Y60A3A.1 | 1.576 | 0.115 | 1.62 | 0.044 | *unc-51* | Serine/threonine-protein kinase involved in autophagy |
| F11A1.2 | 5.844 | 0.15 | 1.62 | 0.036 |  |  |
| F28B1.5 | 1.417 | 0.121 | 1.62 | 0.037 |  | Unnamed protein |
| H02I12.5 | 3.615 | 0.138 | 1.62 | 0.038 |  | Unnamed protein |
| K02D10.2 | 4.059 | 0.0927 | 1.62 | 0.013 |  |  |
| C09G5.5 | 7.390 | 0.149 | 1.62 | 0.050 | *col-80* | Collagens (type IV and type XIII), and related proteins |
| Y65B4BL.7 | 1.366 | 0.179 | 1.62 | 0.046 |  |  |
| T07H8.7 | 5.114 | 0.167 | 1.61 | 0.040 | *sre-12* | Sre G protein-coupled chemoreceptor |
| R10E8.4 | 4.054 | 0.177 | 1.61 | 0.040 |  | Uncharacterized protein |
| Y113G7B.1 | 1.002 | 0.135 | 1.61 | 0.043 |  |  |
| T07C4.4 | 25.674 | 0.083 | 1.61 | 0.007 | *spp-1* | Unnamed protein |
| C18A11.1 | 3.748 | 0.0342 | 1.61 | 0.048 |  | Unnamed protein |
| C06G3.10 | 2.189 | 0.0483 | 1.61 | 0.047 | *cgo-2* | Low density lipoprotein receptor |
| Y61A9LA.8 | 2.330 | 0.114 | 1.61 | 0.049 |  | Nuclear polyadenylated RNA binding protein |
| F28E10.2 | 2.311 | 0.153 | 1.61 | 0.048 |  |  |
| T16A1.3 | 1.649 | 0.0847 | 1.61 | 0.036 |  | F-box domain |
| H21P03.1 | 2.798 | 0.0461 | 1.61 | 0.040 | *mbf-1* | Transcription factor MBF1 |
| C50H11.9 | 1.186 | 0.153 | 1.61 | 0.045 | *str-244* | 7-transmembrane olfactory receptor |
| Y69A2AR.18b | 18.850 | 0.142 | 1.61 | 0.047 |  | F0F1-type ATP synthase, gamma subunit |
| T02E1.4 | 1.976 | 0.155 | 1.60 | 0.036 |  | Hydroxysteroid 17-beta dehydrogenase 11 |
| K02D10.1b | 3.785 | 0.111 | 1.60 | 0.048 |  |  |
| T14E8.3 | 2.455 | 0.104 | 1.60 | 0.040 | *dop-3* |  |
| Y41G9A.6 | 4.186 | 0.105 | 1.60 | 0.047 |  |  |
| K12D9.5 | 8.518 | 0.145 | 1.60 | 0.035 | *srw-120* | 7-transmembrane olfactory receptor |
| Y46C8AL.4 | 2.487 | 0.198 | 1.59 | 0.044 |  | C-type lectin |
| K02A6.1 | 1.687 | 0.108 | 1.59 | 0.020 |  |  |
| T21B4.5 | 28.783 | 0.123 | 1.59 | 0.038 | *srh-68* | Predicted olfactory G-protein coupled receptor |
| F10D11.4 | 21.631 | 0.125 | 1.59 | 0.034 |  | Unnamed protein |
| C04F12.7 | 3.417 | 0.196 | 1.59 | 0.041 |  | Unnamed protein |
| Y54F10AM.4c | 2.321 | 0.201 | 1.59 | 0.051 | *ceh-44* | Transcription factor/CCAAT displacement protein CDP1 |
| B0336.2 | 3.678 | 0.209 | 1.59 | 0.043 | *arf-1.2* | GTP-binding ADP-ribosylation factor Arf1 |
| F10G7.5 | 2.054 | 0.15 | 1.59 | 0.046 |  | Monocarboxylate transporter |
| ZK1225.4 | 3.986 | 0.145 | 1.59 | 0.041 |  | Splicing factor RNPS1, SR protein superfamily |
| C28G1.3 | 4.451 | 0.227 | 1.59 | 0.049 |  | Exocyst complex, subunit SEC15 |
| C05E4.9a | 4.184 | 0.169 | 1.58 | 0.043 | *gei-7* | Isocitrate lyase |
| C05E7.2 | 1.219 | 0.157 | 1.58 | 0.043 |  | Unnamed protein |
| C08A9.8 | 11.282 | 0.071 | 1.57 | 0.031 |  | Uncharacterized protein |
| C04B4.2 | 1.189 | 0.0566 | 1.57 | 0.001 |  | Unnamed protein |
| R09B5.9 | 2.639 | 0.22 | 1.57 | 0.036 |  | Predicted chitinase |
| F44F4.7 | 1.122 | 0.15 | 1.57 | 0.049 | *sra-12* | Sra family integral membrane protein |
| C02C6.3 | 1.026 | 0.0428 | 1.56 | 0.048 |  | Leucine rich repeat |
| K09B11.9 | 22.216 | 0.0532 | 1.56 | 0.042 |  |  |
| F57B10.5 | 1.973 | 0.0783 | 1.56 | 0.035 |  | emp24/gp25L/p24 family of membrane trafficking proteins |
| Y46H3C.1 | 3.025 | 0.0959 | 1.56 | 0.044 | *srw-100* | 7-transmembrane olfactory receptor |
| K02F6.3 | 2.462 | 0.0722 | 1.56 | 0.035 |  | WSN domain |
| H20J04.2 | 1.796 | 0.0735 | 1.56 | 0.046 |  | Chromatin remodeling complex WSTF-ISWI |
| F40G9.2 | 2.329 | 0.0734 | 1.56 | 0.041 |  | Cytochrome c oxidase assembly protein/Cu2+ chaperone COX17 |
| Y17D7A.3 | 1.022 | 0.149 | 1.55 | 0.038 | *nhr-65* |  |
| W02D9.7 | 4.126 | 0.158 | 1.55 | 0.037 |  |  |
| Y66A7A.6 | 1.101 | 0.151 | 1.55 | 0.037 | *gly-8* | Polypeptide N-acetylgalactosaminyltransferase |
| T06F4.1b | 2.552 | 0.213 | 1.55 | 0.044 |  | Unnamed protein |
| T23B12.5 | 1.491 | 0.194 | 1.55 | 0.037 |  | FGF receptor activating protein 1 |
| Y74C9A.1 | 2.438 | 0.0996 | 1.55 | 0.044 |  | Unnamed protein |
| K11H3.2 | 2.064 | 0.0642 | 1.55 | 0.034 |  |  |
| F40F8.7 | 4.353 | 0.0672 | 1.55 | 0.015 | *pqm-1* | Zn-finger |

| K04A8.9 | 1.822 | 0.116 | 1.55 | 0.035 | *spp-19* |  |
| --- | --- | --- | --- | --- | --- | --- |
| F48F5.1 | 1.295 | 0.144 | 1.55 | 0.040 |  | Protein tyrosine phosphatase, contains WSN domain |
| T26G10.1 | 1.482 | 0.0875 | 1.55 | 0.018 |  | ATP-dependent RNA helicase |
| R04D3.7 | 3.271 | 0.108 | 1.55 | 0.017 | *srd-42* | Chemoreceptor/7TM receptor |
| Y71D11A.2 | 1.481 | 0.0999 | 1.55 | 0.044 | *smr-1* | Splicing factor SPF30 |
| R11F4.3 | 2.474 | 0.0376 | 1.54 | 0.013 |  | Unnamed protein |
| R07B7.11 | 10.564 | 0.11 | 1.54 | 0.031 | *gana-1* | Alpha-D-galactosidase (melibiase) |
| F53F4.10 | 3.620 | 0.155 | 1.54 | 0.035 |  | NADH:ubiquinone oxidoreductase, NDUFV2/24 kD subunit |
| F17C11.2 | 1.538 | 0.062 | 1.54 | 0.011 |  | Unnamed protein |
| M6.1a | 1.674 | 0.0772 | 1.54 | 0.049 | *ifc-2* | Nuclear envelope protein lamin, intermediate filament superfamily |
| K11H12.2 | 46.942 | 0.161 | 1.53 | 0.035 | *rpl-15* | 60s ribosomal protein L15 |
| T22E7.1 | 1.140 | 0.115 | 1.53 | 0.049 |  | Leucine rich repeat |
| F13G11.2 | 2.477 | 0.208 | 1.53 | 0.041 |  |  |
| C46C11.3 | 2.039 | 0.12 | 1.53 | 0.031 |  | Unnamed protein |
| ZC404.10 | 2.735 | 0.0942 | 1.52 | 0.032 |  | Unnamed protein |
| F36A4.7 | 2.699 | 0.0778 | 1.52 | 0.047 | *ama-1* | RNA polymerase II, large subunit |
| Y54E5A.6 | 1.580 | 0.193 | 1.51 | 0.042 |  | tRNA-dihydrouridine synthase |
| F18C5.2 | 1.214 | 0.096 | 1.51 | 0.050 | *wrn-1* | ATP-dependent DNA helicase |
| Y82E9BL.14 | 1.024 | 0.14 | 1.51 | 0.051 |  | Uncharacterized protein |
| F53H1.1a | 1.744 | 0.115 | 1.50 | 0.046 |  | RNA helicase |
| Y50E8A.9 | 3.257 | 0.0662 | 1.50 | 0.043 |  | Phospholipid scramblase |
| F58A3.5 | 15.080 | 0.196 | 1.50 | 0.035 |  | Uncharacterized protein with conserved cysteine |
| T05C7.1 | 2.484 | 0.173 | 1.50 | 0.040 |  |  |
| Y51H1A.3b | 1.034 | 0.22 | 1.50 | 0.042 |  | NADH:ubiquinone oxidoreductase, NDUFB8/ASHI subunit |
| T08G5.1 | 50.746 | 0.0942 | 1.50 | 0.040 |  |  |
| F47G9.6 | 7.307 | 0.067 | 1.49 | 0.010 |  | Unnamed protein |
| ZK858.7 | 2.929 | 0.136 | 1.49 | 0.037 |  | tRNA(1-methyladenosine) methyltransferase, subunit GCD10 |
| F58E10.5 | 18.649 | 0.0634 | 1.49 | 0.040 | *end-3* | GATA-4/5/6 transcription factors |
| F13H8.10c | 767,2 | 0.113 | 1.49 | 0.051 | *bpl-1* |  |
| F55H12.3 | 3.423 | 0.0962 | 1.49 | 0.044 |  | Fibrillins and related Ca2+-binding EGF-like domains |
| K08C7.6 | 34.937 | 0.157 | 1.49 | 0.038 |  |  |
| W08E12.7 | 3.279 | 0.0859 | 1.48 | 0.042 |  | Metallopeptidase |
| F53G12.5a | 4.423 | 0.105 | 1.48 | 0.044 | *mex-3* | Predicted RNA binding protein, contains KH domain |
| C17H12.6 | 1.014 | 0.134 | 1.47 | 0.039 |  | Confirmed TR:O46340 AAB88318.1 |
| T13F3.4 | 1.254 | 0.095 | 1.47 | 0.038 |  | Predicted secreted cysteine rich protein found only in C.elegans |
| T09A5.10 | 1.965 | 0.123 | 1.47 | 0.041 | *lin-5* | Unnamed protein |
| Y68A4A.9 | 5.374 | 0.111 | 1.47 | 0.041 |  | No Significant Match |
| C26B2.8 | 1.174 | 0.0823 | 1.47 | 0.027 |  |  |
| F57A8.1 | 846 | 0.121 | 1.47 | 0.044 |  |  |
| T04F3.1 | 1.383 | 0.124 | 1.47 | 0.050 |  | 1-aminocyclopropane-1-carboxylate synthase, and related proteins |
| W02D7.6 | 1.586 | 0.0939 | 1.47 | 0.049 |  | C2H2-type Zn-finger |
| K05B2.5 | 5.884 | 0.117 | 1.47 | 0.049 | *pes-22* | Monocarboxylate transporter |
| T22E5.1 | 1.878 | 0.107 | 1.46 | 0.050 |  |  |
| C02B8.4 | 1.982 | 0.14 | 1.45 | 0.044 | *hlh-8* | Transcription factor TWIST |
| F54F2.2b | 1.467 | 0.201 | 1.45 | 0.045 | *zfp-1* |  |
| F58G6.1 | 1.597 | 0.11 | 1.45 | 0.046 |  | Amphiphysin |
| K07B1.4 | 2.466 | 0.0903 | 1.45 | 0.046 |  |  |
| K10D2.6 | 6.586 | 0.125 | 1.45 | 0.037 |  | NADP/FAD dependent oxidoreductase |
| C01G8.1 | 1.140 | 0.174 | 1.45 | 0.032 |  |  |
| F11G11.2 | 4.290 | 0.132 | 1.44 | 0.032 | *gst-7* | Glutathione S-transferase |
| B0213.6 | 8.741 | 0.135 | 1.44 | 0.038 | *cyp-34A8* | Cytochrome P450 CYP2 subfamily |
| T05H10.2 | 1.736 | 0.112 | 1.44 | 0.047 | *apn-1* | Major apurinic/apyrimidinic endonuclease/3'-repair diesterase APN1 |
| D1054.3 | 2.659 | 0.111 | 1.44 | 0.048 |  | Suppressor of G2 allele of skp1 |
| M01D1.3 | 46.447 | 0.131 | 1.44 | 0.043 |  | BTB domain protein |
| R13G10.1 | 1.277 | 0.101 | 1.43 | 0.043 | *dpy-27* | Structural maintenance of chromosome protein 4 |
| T28B8.1 | 2.460 | 0.0771 | 1.43 | 0.037 |  |  |
| B0213.4 | 27.940 | 0.0855 | 1.43 | 0.038 |  | Confirmed |
| R05D11.1 | 9.365 | 0.102 | 1.42 | 0.023 |  | TGFbeta receptor signaling protein SMAD and related proteins |

| C25A1.13 | 1.645 | 0.109 | 1.42 | 0.033 |  | Mitochondrial/chloroplast ribosomal protein 36a |
| --- | --- | --- | --- | --- | --- | --- |
| T11A5.3 | 6.048 | 0.0518 | 1.42 | 0.031 |  | No Significant Match |
| T05F1.7 | 41.393 | 0.0858 | 1.41 | 0.036 |  |  |
| Y53H1C.1 | 1.659 | 0.128 | 1.41 | 0.043 | *aat-9* |  |
| H10E21.1 | 1.657 | 0.147 | 1.41 | 0.050 |  |  |
| R06C1.1 | 3.936 | 0.073 | 1.41 | 0.040 | *hda-3* | Histone deacetylase complex, catalytic component RPD3 |
| Y66D12A.12 | 3.689 | 0.12 | 1.41 | 0.047 |  | Zn-finger |
| DOWN-REGULATED GENES | | | | | | |
| F40F9.5 | 25.274 | 0.056 | 0.71 | 0.034 |  | Predicted transporter ADD1 (major facilitator superfamily) |
| T20B3.6 | 1.269 | 0.0685 | 0.71 | 0.042 | *phy-3* | Prolyl 4-hydroxylase alpha subunit |
| Y39A3B.1 | 465.5 | 0.0926 | 0.70 | 0.043 |  | Aminopeptidases of the M20 family |
| Y110A2AL.7 | 847.3 | 0.089 | 0.70 | 0.043 |  | Unnamed protein |
| T09D3.4 | 1.224 | 0.0977 | 0.70 | 0.040 |  | No Significant Match |
| F02D8.2 | 979.9 | 0.0541 | 0.70 | 0.034 | *grd-12* | Unnamed protein |
| F30B5.7 | 1.023 | 0.102 | 0.70 | 0.047 |  | Fe2+/Zn2+ regulated transporter |
| C05D10.1b | 1.133 | 0.0623 | 0.69 | 0.049 |  |  |
| C53B4.1 | 932.6 | 0.105 | 0.69 | 0.048 |  | Synaptic vesicle transporter SVOP and related transporters |
| F17H10.2 | 1.385 | 0.11 | 0.69 | 0.024 |  | Unnamed protein |
| Y59H11AR.1 | 788.4 | 0.0806 | 0.69 | 0.045 |  |  |
| T24F1.3 | 1.418 | 0.0822 | 0.68 | 0.044 |  |  |
| R09A8.1 | 883 | 0.0797 | 0.68 | 0.022 |  | Unnamed protein |
| ZC190.6 | 1.064 | 0.0782 | 0.68 | 0.041 |  | Unnamed protein |
| F59F5.8 | 843.2 | 0.0979 | 0.68 | 0.045 |  |  |
| C11H1.8 | 747.6 | 0.0954 | 0.68 | 0.049 |  |  |
| T14B4.6 | 573.2 | 0.122 | 0.68 | 0.027 | *dpy-2* | Collagens (type IV and type XIII), and related proteins |
| T16H12.9 | 1.339 | 0.0754 | 0.67 | 0.047 |  | Unnamed protein |
| C05E11.4 | 2.359 | 0.0622 | 0.67 | 0.031 | *amt-1* | Ammonia permease |
| F45E4.9 | 674.1 | 0.106 | 0.67 | 0.034 | *hmg-5* | HMG box-containing protein |
| T10H4.4 | 1.033 | 0.0796 | 0.67 | 0.038 |  | Phytanoyl-CoA hydroxylase associated protein |
| C04E12.11 | 1.437 | 0.0369 | 0.67 | 0.048 |  |  |
| C03H5.5 | 718.7 | 0.0933 | 0.67 | 0.033 |  | RNA polymerase I-associated factor - PAF67 |
| F46C5.8 | 612.1 | 0.0663 | 0.67 | 0.025 |  | Golgi proteins involved in ER retention (RER) |
| C26D10.6 | 1.205 | 0.0745 | 0.66 | 0.051 |  | Unnamed protein |
| Y57G11A.2 | 2.426 | 0.0479 | 0.66 | 0.038 |  | Uncharacterized protein |
| F09F7.5c | 1.559 | 0.112 | 0.66 | 0.048 |  | Unnamed protein |
| B0302.1a | 1.180 | 0.164 | 0.66 | 0.042 | *kin-25* |  |
| ZK1128.3 | 853 | 0.0707 | 0.66 | 0.047 |  |  |
| M02B1.2 | 2.001 | 0.0642 | 0.65 | 0.036 |  | Reticulocalbin, calumenin, DNA supercoiling factor |
| C28C12.7a | 1.296 | 0.0939 | 0.65 | 0.048 | *spp-10* | Prosaposin |
| Y51F10.2 | 951.1 | 0.102 | 0.65 | 0.049 |  |  |
| Y94A7B.5 | 889.5 | 0.114 | 0.65 | 0.041 | *srh-298* | Predicted olfactory G-protein coupled receptor |
| R10E8.6 | 527.3 | 0.0777 | 0.65 | 0.044 |  | Uncharacterized protein |
| R12E2.6 | 546.5 | 0.0777 | 0.64 | 0.040 |  | Unnamed protein |
| K06C4.9 | 922 | 0.0346 | 0.64 | 0.048 |  | Unnamed protein |
| W01G7.5 | 1.264 | 0.061 | 0.64 | 0.042 | *lem-2* | Unnamed protein |
| Y46C8AL.5 | 1.447 | 0.104 | 0.64 | 0.050 |  | C-type lectin |
| D2023.6 | 1.005 | 0.111 | 0.64 | 0.033 |  | Protein phosphatase 4 regulatory subunit 2 related protein |
| R17.2 | 1.785 | 0.103 | 0.64 | 0.038 |  | Glucose-repressible alcohol dehydrogenase transcriptional effector |
| W08F4.7 | 1.484 | 0.121 | 0.64 | 0.037 |  |  |
| C16C10.2 | 876 | 0.0914 | 0.64 | 0.044 |  | Uncharacterized conserved protein |
| ZK1067.4 | 944.1 | 0.114 | 0.64 | 0.049 |  | Predicted membrane protein |
| C44F1.2 | 1.492 | 0.0808 | 0.64 | 0.049 |  | Nuclear DEAF-1 related transcriptional regulator (suppressin) |
| ZK177.8b | 2.775 | 0.0782 | 0.63 | 0.045 |  | Metal-dependent phosphohydrolase |
| B0462.1 | 626.3 | 0.0877 | 0.63 | 0.037 |  | Uncharacterized protein |
| C09H10.7 | 1.023 | 0.066 | 0.63 | 0.002 |  |  |
| ZC504.5 | 653.9 | 0.113 | 0.63 | 0.049 | *gur-3* | Unnamed protein |
| ZK662.3a | 1.263 | 0.143 | 0.63 | 0.040 | *nhr-48* |  |
| EEED8.8 | 474.1 | 0.108 | 0.63 | 0.046 | *ndx-6* | Transient receptor potential-related channel 7 |

| C26E1.1 | 919.2 | 0.11 | 0.63 | 0.033 |  |  |
| --- | --- | --- | --- | --- | --- | --- |
| K12B6.3 | 1.839 | 0.0626 | 0.63 | 0.035 |  | Triacylglycerol lipase |
| C05D11.11a | 761.1 | 0.0898 | 0.62 | 0.036 | *mel-32* | Glycine/serine hydroxymethyltransferase |
| C44B11.4 | 1.018 | 0.0699 | 0.62 | 0.048 |  |  |
| F21C3.5 | 1.372 | 0.147 | 0.62 | 0.049 |  | Prefoldin subunit 6, KE2 family |
| T27E9.5 | 1.173 | 0.0732 | 0.62 | 0.044 |  | Phosphatidylserine synthase |
| F17E5.1a | 11.820 | 0.0604 | 0.62 | 0.046 | *lin-2* | Calcium/calmodulin-dependent serine protein kinase |
| T27A10.6 | 883 | 0.0905 | 0.62 | 0.049 |  |  |
| Y106G6D.3 | 762.6 | 0.0722 | 0.61 | 0.041 |  | Unnamed protein |
| M01G12.5 | 1.007 | 0.0943 | 0.61 | 0.040 |  | Serpin |
| C07H6.7 | 1.007 | 0.0676 | 0.61 | 0.003 | *lin-39* | Transcription factor zerknullt and related HOX domain proteins |
| F35F10.9 | 781.8 | 0.102 | 0.61 | 0.034 | *srbc-1* | Unnamed protein |
| T05H4.5 | 705.4 | 0.0841 | 0.61 | 0.036 |  | NADH-cytochrome b-5 reductase |
| C37A5.7 | 595 | 0.0803 | 0.61 | 0.039 |  | Uncharacterized conserved protein |
| C49F8.3 | 635.2 | 0.147 | 0.61 | 0.046 |  | Unnamed protein |
| C53C7.1 | 687.2 | 0.0564 | 0.61 | 0.041 |  |  |
| F53H4.2 | 1.000 | 0.0895 | 0.61 | 0.035 |  | Unnamed protein |
| Y44A6E.1 | 1.209 | 0.0651 | 0.61 | 0.050 |  |  |
| F28G4.4 | 935.6 | 0.0661 | 0.60 | 0.038 |  | Uncharacterized protein |
| C28H8.3 | 1.689 | 0.0598 | 0.60 | 0.048 |  | Predicted helicase, DEAD-box superfamily |
| R11D1.3 | 729.6 | 0.0924 | 0.60 | 0.031 |  | Small secreted protein with conserved cysteines |
| ZC477.10 | 1.243 | 0.0768 | 0.60 | 0.050 |  | Serine/threonine specific protein phosphatase PP1 |
| C27C12.4 | 5.334 | 0.128 | 0.60 | 0.043 |  | Uncharacterized conserved protein |
| Y111B2A.15 | 863.4 | 0.132 | 0.60 | 0.045 |  | Protein-tyrosine sulfotransferase TPST1/TPST2 |
| F59F5.3 | 7.207 | 0.0821 | 0.60 | 0.049 |  | Fibroblast/platelet-derived growth factor receptor |
| ZC374.2 | 1.525 | 0.0938 | 0.60 | 0.049 |  |  |
| F45B8.3 | 1.939 | 0.169 | 0.60 | 0.043 |  |  |
| T04C4.1 | 840.4 | 0.0635 | 0.60 | 0.049 |  | Uncharacterized conserved protein |
| Y92C3A.1 | 868.3 | 0.0999 | 0.60 | 0.046 |  | No Significant Match |
| JC8.7 | 2.000 | 0.0876 | 0.59 | 0.040 |  | Uncharacterized conserved protein |
| Y49E10.9 | 1.813 | 0.0526 | 0.59 | 0.043 | *tag-222* | Transporter, ABC superfamily (Breast cancer resistance protein) |
| K04C1.3 | 501.9 | 0.123 | 0.59 | 0.047 |  | Unnamed protein |
| ZC8.1 | 517.9 | 0.123 | 0.59 | 0.032 |  | C-3 sterol dehydrogenase/3-beta-hydroxysteroid dehydrogenase |
| C15H7.4 | 649.4 | 0.0924 | 0.58 | 0.039 |  | Unnamed protein |
| F25H5.7 | 893.6 | 0.0257 | 0.58 | 0.037 |  | Protein tyrosine phosphatase |
| C09G12.9 | 612.7 | 0.075 | 0.58 | 0.041 |  | Vacuolar sorting protein/ubiquitin receptor VPS23 |
| C06B8.2a | 546.5 | 0.102 | 0.58 | 0.050 |  |  |
| T06C10.6 | 830.6 | 0.169 | 0.58 | 0.049 | *kin-26* | Protein tyrosine kinase |
| K12D9.11 | 591.1 | 0.126 | 0.58 | 0.035 | *sodh-2* | Alcohol dehydrogenase, class V |
| T28A11.9 | 1.037 | 0.0911 | 0.58 | 0.037 | *srj-8* | 7-transmembrane olfactory receptor |
| C11G10.1 | 1.068 | 0.0826 | 0.58 | 0.003 |  |  |
| K07A12.7 | 837.6 | 0.0757 | 0.58 | 0.045 |  | Mitochondrial/choloroplast ribosomal protein S15 |
| T26A8.3 | 856.1 | 0.0564 | 0.57 | 0.032 |  | Unnamed protein |
| ZC196.7 | 1.536 | 0.122 | 0.57 | 0.037 | *glr-5* | Glutamate-gated kainate-type ion channel receptor subunit GluR5 |
| W03H9.4 | 925.5 | 0.133 | 0.57 | 0.042 |  | Cactin |
| T24B8.5 | 841.9 | 0.106 | 0.57 | 0.015 |  | Secreted surface protein |
| Y54G2A.14 | 1.160 | 0.0663 | 0.56 | 0.044 |  | C-type lectin |
| C03A7.12 | 895.6 | 0.11 | 0.56 | 0.047 |  | UDP-glucuronosyl and UDP-glucosyl transferase |
| C33F10.5b | 640.1 | 0.123 | 0.56 | 0.045 | *rig-6* |  |
| C35D10.14 | 747.3 | 0.0411 | 0.56 | 0.046 | *clec-5* | C-type lectin |
| F10F2.9 | 1.051 | 0.158 | 0.56 | 0.028 | *pqn-29* |  |
| Y105E8A.17 | 654.6 | 0.14 | 0.56 | 0.048 |  | DNA methyltransferase 1-associated protein-1 |
| K03A1.4 | 1.117 | 0.12 | 0.56 | 0.034 |  | Calmodulin and related proteins (EF-Hand superfamily) |
| T22F3.7 | 1.256 | 0.0926 | 0.56 | 0.037 |  | Permease of the major facilitator superfamily |
| T09B4.4 | 1.077 | 0.0711 | 0.56 | 0.036 |  | Calmodulin and related proteins (EF-Hand superfamily) |
| C17F4.3 | 641.3 | 0.157 | 0.56 | 0.033 |  | Unnamed protein |
| K09F6.3 | 698.2 | 0.117 | 0.56 | 0.049 |  | Protein tyrosine phosphatase |
| F23F1.3 | 574.7 | 0.143 | 0.55 | 0.050 |  | F-box domain |

| F10E7.3 | 1.085 | 0.15 | 0.55 | 0.037 |  |  |
| --- | --- | --- | --- | --- | --- | --- |
| T04F8.1 | 661.8 | 0.149 | 0.55 | 0.025 |  | Sideroflexin |
| Y47H9C.1 | 884.1 | 0.0938 | 0.55 | 0.042 |  | Uncharacterized protein |
| C44C10.2 | 483.2 | 0.104 | 0.55 | 0.045 |  | Cytochrome P450 CYP4/CYP19/CYP26 subfamilies |
| ZK697.2 | 1.055 | 0.142 | 0.55 | 0.037 |  | Hormone receptors |
| W03F8.5 | 434 | 0.153 | 0.55 | 0.039 |  |  |
| F10E9.7 | 689.6 | 0.0859 | 0.55 | 0.048 |  |  |
| ZC395.1 | 547.3 | 0.124 | 0.55 | 0.048 |  |  |
| F35H12.5 | 447.9 | 0.0732 | 0.55 | 0.048 |  | Unnamed protein |
| C16C2.3 | 918.6 | 0.104 | 0.54 | 0.039 | *ocrl-1* | Inositol polyphosphate 5-phosphatase and related proteins |
| Y43B11AR.1 | 1.003 | 0.144 | 0.54 | 0.046 |  |  |
| F25E5.13 | 448.6 | 0.093 | 0.54 | 0.044 | *srh-92* | Predicted olfactory G-protein coupled receptor |
| ZK666.10 | 1.108 | 0.137 | 0.53 | 0.038 |  | Confirmed |
| F54B8.1 | 1.271 | 0.153 | 0.53 | 0.038 |  | Uncharacterized protein |
| ZC404.8 | 676 | 0.113 | 0.53 | 0.032 | *spn-4* | Ataxin 2-binding protein (RRM superfamily) |
| Y73B3A.20 | 935 | 0.17 | 0.53 | 0.048 |  |  |
| K06B9.4 | 686.6 | 0.0892 | 0.53 | 0.047 |  |  |
| F20H11.5 | 585.3 | 0.163 | 0.53 | 0.034 |  | D-aspartate oxidase |
| C25D7.4 | 738 | 0.14 | 0.52 | 0.033 |  | Uncharacterized protein |
| Y59H11AM.1 | 592.4 | 0.0895 | 0.52 | 0.045 |  | Major sperm protein domain |
| ZK809.4 | 1.572 | 0.0699 | 0.52 | 0.050 | *ent-1* | Nucleoside transporter |
| F01D4.2 | 851.3 | 0.123 | 0.52 | 0.033 | *ugt-44* | UDP-glucuronosyl and UDP-glucosyl transferase |
| Y92H12BL.4 | 819.6 | 0.098 | 0.52 | 0.051 |  | Predicted GTPase activating protein |
| F37A4.3 | 644.9 | 0.106 | 0.52 | 0.005 |  |  |
| ZK1236.6 | 786.6 | 0.151 | 0.52 | 0.039 | *pqn-96* |  |
| F53A9.1 | 1.403 | 0.126 | 0.52 | 0.048 |  | Predicted TR:Q20690 AAC46556.1 |
| C04E12.4 | 467.6 | 0.0514 | 0.52 | 0.049 |  | Predicted peptide:N-glycanase |
| B0414.2 | 1.061 | 0.126 | 0.52 | 0.044 | *rnt-1* | Runt and related transcription factors |
| T02E1.1 | 327 | 0.0928 | 0.52 | 0.036 | *spe-12* |  |
| F41D3.11 | 1.010 | 0.128 | 0.52 | 0.038 |  | Glycosyltranferase |
| C05D2.1c | 502.7 | 0.182 | 0.52 | 0.050 | *daf-4* |  |
| M28.6 | 946.3 | 0.164 | 0.51 | 0.010 |  | Predicted esterase |
| C36A4.6 | 619.4 | 0.115 | 0.51 | 0.013 | *cyp-25A4* | Cytochrome P450 CYP3/CYP5/CYP6/CYP9 subfamilies |
| C16C8.2 | 816.7 | 0.128 | 0.51 | 0.043 |  | Peroxidase/oxygenase |
| F01G4.5 | 907.6 | 0.0969 | 0.51 | 0.033 |  | N-acetylglucosaminyltransferase complex, subunit PIG-Q/GPI1 |
| T15H9.3 | 768.9 | 0.0552 | 0.50 | 0.009 | *hlh-6* | Transcription factor HAND2/Transcription factor TAL1/TAL2/LYL1 |
| T01G1.1 | 1.008 | 0.129 | 0.50 | 0.036 | *klp-12* |  |
| F14B8.5b | 728 | 0.123 | 0.50 | 0.048 |  | Unnamed protein |
| C33E10.10 | 910.8 | 0.0746 | 0.50 | 0.039 |  | Predicted dehydrogenase |
| F40F12.5b | 1.194 | 0.126 | 0.50 | 0.043 |  | Familial cylindromatosis protein |
| Y43F4B.4 | 620 | 0.168 | 0.50 | 0.038 | *npp-18* | Nuclear pore complex component (sc Seh1) |
| T28D9.2d | 774.5 | 0.125 | 0.50 | 0.048 | *rsp-5* | Alternative splicing factor SRp55/B52/SRp75 (RRM superfamily) |
| T21C9.7 | 881.9 | 0.143 | 0.49 | 0.031 | *srg-32* | Receptor-like protein, Srg family |
| F55G11.5 | 1.765 | 0.164 | 0.49 | 0.040 | *dod-22* | Uncharacterized protein |
| F02C12.2 | 465.1 | 0.137 | 0.49 | 0.026 |  | Reductases with broad range of substrate specificities |
| F46H5.4 | 542.3 | 0.115 | 0.49 | 0.012 |  | PH domain-containing protein |
| F45C12.2 | 511.1 | 0.0944 | 0.49 | 0.034 |  |  |
| C08F11.5 | 796.9 | 0.062 | 0.49 | 0.039 |  | Uncharacterized protein |
| ZC84.5 | 944 | 0.0949 | 0.49 | 0.001 |  | No Significant Match |
| C03F11.2 | 838.5 | 0.203 | 0.49 | 0.036 |  | Uncharacterized conserved protein |
| F47E1.1 | 395.4 | 0.148 | 0.49 | 0.046 |  | Unnamed protein |
| Y54G2A.24 | 738.1 | 0.126 | 0.49 | 0.046 |  |  |
| W10G11.11 | 936.5 | 0.0859 | 0.49 | 0.037 |  | Cysteine rich domain (CW domain) |
| ZC196.4 | 766.1 | 0.192 | 0.48 | 0.037 |  | Unnamed protein |
| Y43D4A.6 | 1.136 | 0.121 | 0.48 | 0.043 |  | Checkpoint kinase and related serine/threonine protein kinases |
| F26H9.2 | 803.2 | 0.161 | 0.48 | 0.034 |  | RPEL repeat-containing protein |
| F31D4.6 | 809.4 | 0.127 | 0.48 | 0.038 | *try-4* | Trypsin |
| Y54E2A.6 | 815 | 0.0759 | 0.48 | 0.042 |  | DNA/RNA helicase MER3/SLH1, DEAD-box superfamily |

| Y43F8C.4 | 857.4 | 0.149 | 0.48 | 0.043 |  |  |
| --- | --- | --- | --- | --- | --- | --- |
| C33E10.6 | 4.018 | 0.0315 | 0.48 | 0.043 |  | Unnamed protein |
| F12E12.4 | 642.4 | 0.173 | 0.48 | 0.045 |  | Uncharacterized protein, contains BTB/POZ domain |
| T20B12.8 | 739.2 | 0.158 | 0.48 | 0.036 | *hmg-4* | Nucleosome-binding factor SPN, POB3 subunit |
| C50A2.2 | 735.3 | 0.122 | 0.48 | 0.039 |  | Unnamed protein |
| T09F5.9 | 1.262 | 0.115 | 0.48 | 0.036 | *clec-47* | C-type lectin |
| K09E3.6 | 1.046 | 0.167 | 0.47 | 0.039 |  | Uncharacterized protein |
| Y39A1A.22 | 616.1 | 0.126 | 0.47 | 0.041 |  | Predicted small molecule transporter |
| Y34B4A.5 | 490.9 | 0.0721 | 0.47 | 0.046 |  | Uncharacterized protein |
| C15A7.2 | 673.3 | 0.145 | 0.47 | 0.048 |  | Predicted membrane protein |
| C49G7.9 | 1.179 | 0.0795 | 0.47 | 0.033 |  |  |
| D2092.2 | 626.6 | 0.195 | 0.47 | 0.033 | *ssp-11* | Uncharacterized protein, contains major sperm proteindomain |
| T06A4.1 | 1.238 | 0.144 | 0.47 | 0.047 |  |  |
| D2096.3 | 537.5 | 0.154 | 0.47 | 0.015 |  | Maltase glucoamylase and related hydrolases |
| T10C6.10 | 665.9 | 0.174 | 0.47 | 0.038 |  | Uncharacterized protein |
| C01C7.1 | 562.8 | 0.103 | 0.47 | 0.045 | *ark-1* | ACK and related non-receptor tyrosine kinases |
| ZK593.3 | 667.5 | 0.195 | 0.47 | 0.031 |  |  |
| C33F10.12 | 937.9 | 0.195 | 0.46 | 0.046 |  | Mitochondrial phosphate carrier protein |
| F46C5.9 | 474.3 | 0.185 | 0.46 | 0.034 |  | WD40 repeat protein |
| T07D10.3 | 1.197 | 0.0826 | 0.46 | 0.036 |  | Unnamed protein |
| Y71G12B.23 | 644.2 | 0.143 | 0.46 | 0.046 |  |  |
| F32B5.1 | 661.7 | 0.0723 | 0.46 | 0.047 |  | Creatine kinases |
| C30A5.10b | 772.8 | 0.14 | 0.46 | 0.048 |  | Unnamed protein |
| M7.9 | 497.2 | 0.148 | 0.46 | 0.036 |  | Uncharacterized conserved protein |
| R10E11.8 | 547.7 | 0.161 | 0.46 | 0.031 | *vha-1* | Vacuolar H+-ATPase V0 sector, subunits c/c' |
| C16B8.4 | 760.8 | 0.0565 | 0.45 | 0.049 |  |  |
| Y32B12A.5 | 453.8 | 0.189 | 0.45 | 0.042 |  |  |
| ZK1010.5 | 1.044 | 0.157 | 0.45 | 0.037 |  | Unnamed protein |
| T22B11.3 | 686.4 | 0.165 | 0.45 | 0.039 |  | Protein tyrosine kinase |
| T27D12.4 | 1.480 | 0.136 | 0.45 | 0.045 | *pes-5* | Transposon-encoded proteins with TYA, reverse transcriptase |
| T27F6.2 | 681.9 | 0.0843 | 0.45 | 0.038 | *clec-12* | C-type lectin |
| Y40D12A.3 | 729.5 | 0.215 | 0.45 | 0.045 | *srh-40* | Predicted olfactory G-protein coupled receptor |
| B0507.5 | 516 | 0.202 | 0.45 | 0.032 |  |  |
| C05E4.1 | 697 | 0.124 | 0.44 | 0.033 | *srp-2* | Serpin |
| R186.1 | 816.7 | 0.135 | 0.44 | 0.036 |  | Uncharacterized conserved protein |
| W04D2.3 | 565.4 | 0.112 | 0.44 | 0.031 | *inx-11* | Innexin-type channels |
| F11A1.3c | 720.7 | 0.196 | 0.44 | 0.046 | *daf-12* | Unnamed protein |
| C25G4.8 | 727 | 0.158 | 0.44 | 0.030 |  | Uncharacterized protein |
| Y48B6A.12 | 971.2 | 0.154 | 0.43 | 0.043 |  | NADP+-dependent malic enzyme |
| C24A1.4 | 606 | 0.118 | 0.43 | 0.033 |  | No Significant Match |
| F08G2.7 | 1.073 | 0.127 | 0.43 | 0.041 |  | Unnamed protein |
| B0213.14 | 474.7 | 0.241 | 0.43 | 0.039 |  | Unnamed protein |
| C15H11.4 | 812.8 | 0.177 | 0.43 | 0.033 | *dhs-22* | Dehydrogenases with different specificities |
| Y52B11A.3 | 582.5 | 0.0761 | 0.43 | 0.042 |  | NADH-cytochrome b-5 reductase |
| E01A2.4 | 814.8 | 0.225 | 0.43 | 0.044 |  | Uncharacterized conserved protein |
| C35B1.1 | 920.2 | 0.0578 | 0.43 | 0.046 | *ubc-1* | Ubiquitin-protein ligase |
| C49H3.9 | 2.031 | 0.0927 | 0.43 | 0.046 |  | Uncharacterized conserved protein |
| F07A5.4 | 757.4 | 0.15 | 0.43 | 0.047 |  |  |
| F34D10.3 | 794.6 | 0.161 | 0.43 | 0.025 |  |  |
| F59H6.9 | 573.2 | 0.107 | 0.42 | 0.042 |  | Uncharacterized protein, contains BTB/POZ domain |
| F56C11.3 | 426.1 | 0.156 | 0.42 | 0.039 |  | Mitochondrial sulfhydryl oxidase |
| F38B6.4 | 405.1 | 0.137 | 0.42 | 0.047 |  | 5'-phosphoribosylglycinamide formyltransferase |
| C06C3.8 | 604.9 | 0.134 | 0.42 | 0.024 |  | Uncharacterized protein |
| M151.1 | 451.1 | 0.165 | 0.42 | 0.047 |  |  |
| M02A10.1 | 1.048 | 0.13 | 0.41 | 0.032 |  |  |
| Y55B1BM.1b | 418 | 0.116 | 0.41 | 0.049 |  |  |
| W06E11.2 | 750.8 | 0.187 | 0.41 | 0.034 |  |  |
| F01G4.1 | 626.7 | 0.167 | 0.41 | 0.022 | *psa-4* | Chromatin remodeling complex SWI/SNF |

| Y37F4.1 | 807.9 | 0.244 | 0.41 | 0.048 |  |  |
| --- | --- | --- | --- | --- | --- | --- |
| Y57G11A.4 | 686.7 | 0.0617 | 0.41 | 0.051 |  |  |
| C18G1.9 | 578.7 | 0.153 | 0.41 | 0.039 |  | Unnamed protein |
| K10C2.5 | 507 | 0.234 | 0.41 | 0.035 | *grl-6* | GROUND domains |
| B0035.15 | 573.6 | 0.129 | 0.41 | 0.014 |  | Uncharacterized conserved protein |
| T27A10.5 | 446.2 | 0.193 | 0.41 | 0.032 |  |  |
| Y37H2A.7 | 915.9 | 0.212 | 0.40 | 0.044 |  | Uncharacterized protein |
| F10G7.9b | 575.7 | 0.223 | 0.40 | 0.050 |  |  |
| F58G11.6 | 1.008 | 0.181 | 0.40 | 0.035 |  | Putative myrosinase precursor |
| T03D3.6 | 504.4 | 0.158 | 0.39 | 0.036 | *srj-45* | 7-transmembrane olfactory receptor |
| Y51A2D.15 | 1.289 | 0.0955 | 0.39 | 0.050 |  | Uncharacterized coiled-coil protein |
| Y38A10A.6 | 591.7 | 0.216 | 0.39 | 0.047 |  | ATP-dependent RNA helicase |
| Y44A6D.5 | 711.6 | 0.144 | 0.39 | 0.042 |  | Branched chain aminotransferase BCAT1 |
| Y54G2A.6 | 834.9 | 0.17 | 0.39 | 0.046 |  | C-type lectin |
| T06C12.7 | 556.8 | 0.161 | 0.39 | 0.038 | *nhr-84* | Hormone receptors |
| C23H5.7 | 383.2 | 0.161 | 0.39 | 0.039 |  | Cyclic nucleotide-gated cation channel CNGA1-3 |
| F54D10.3 | 1.043 | 0.205 | 0.39 | 0.041 |  | Uncharacterized protein |
| ZK652.5 | 487.6 | 0.09 | 0.38 | 0.029 | *ceh-23* | Transcription factor EMX1 |
| T06C10.3 | 583.4 | 0.219 | 0.38 | 0.045 |  | Protein tyrosine kinase |
| K12G11.4 | 534.3 | 0.188 | 0.38 | 0.035 |  | Predicted alpha-helical protein |
| C25E10.9a | 866.3 | 0.145 | 0.38 | 0.032 | *isl-2* | Uncharacterized protein |
| F48C1.1a | 694 | 0.274 | 0.38 | 0.035 |  | alpha-mannosidase II Partially_confirmed |
| H20E11.1 | 497.9 | 0.119 | 0.38 | 0.041 |  | Unnamed protein |
| T07C4.8 | 671 | 0.172 | 0.38 | 0.044 | *ced-9* | Anti-apoptotic Bcl-2 family proteins |
| AH10.4 | 653 | 0.179 | 0.37 | 0.032 |  | Unnamed protein |
| F40G12.9 | 678.1 | 0.0825 | 0.37 | 0.034 |  | Unnamed protein |
| R08E5.1 | 618.3 | 0.308 | 0.37 | 0.046 |  | SAM-dependent methyltransferases |
| D1005.5 | 511 | 0.0709 | 0.37 | 0.032 |  | No Significant Match |
| W05E10.4 | 469 | 0.141 | 0.36 | 0.031 | *tre-3* | Neutral trehalase |
| C54G7.1 | 5.871 | 0.0997 | 0.36 | 0.006 |  |  |
| Y113G7A.5 | 532.8 | 0.215 | 0.36 | 0.050 |  | Ligand-gated ion channel |
| C25F6.2 | 501.6 | 0.144 | 0.36 | 0.045 | *dlg-1* | Membrane-associated guanylate kinase MAGUK |
| ZK632.13 | 480.9 | 0.199 | 0.36 | 0.033 | *lin-52* | Uncharacterized conserved protein |
| C50F4.9 | 659.9 | 0.18 | 0.36 | 0.035 |  |  |
| B0523.1 | 610.8 | 0.223 | 0.36 | 0.049 | *kin-31* | Protein tyrosine kinase |
| T14B1.2 | 545.3 | 0.132 | 0.35 | 0.050 |  | Unnamed protein |
| R08C7.10c | 750.3 | 0.208 | 0.35 | 0.047 |  | Sister chromatid cohesion protein |
| F09G8.5 | 540.6 | 0.207 | 0.35 | 0.016 |  | Uncharacterized conserved protein |
| F38A1.5 | 652.3 | 0.0942 | 0.34 | 0.039 |  | C-type lectin |
| C02E7.3 | 555 | 0.207 | 0.34 | 0.033 | *srh-20* | Predicted olfactory G-protein coupled receptor |
| C04E6.5 | 472.1 | 0.107 | 0.34 | 0.046 |  | Ubiquitin-specific protease |
| ZK185.2 | 597.2 | 0.283 | 0.33 | 0.050 |  | Predicted divalent cation transporter |
| Y75B8A.16 | 416.4 | 0.262 | 0.33 | 0.044 |  | Predicted G-protein coupled receptor |
| T28D6.3 | 660.4 | 0.0789 | 0.33 | 0.038 |  |  |
| Y75B8A.27 | 549.4 | 0.145 | 0.33 | 0.043 |  |  |
| F48C1.4 | 631.1 | 0.172 | 0.33 | 0.035 |  |  |
| F30F8.7 | 663.1 | 0.192 | 0.32 | 0.034 |  |  |
| B0495.4 | 617 | 0.0807 | 0.32 | 0.048 | *nhx-2* | Sodium/hydrogen exchanger protein |
| F33D11.5 | 465.9 | 0.178 | 0.32 | 0.034 |  | Tandem pore domain K+ channel |
| C25G6.3 | 623.3 | 0.111 | 0.32 | 0.032 |  | Acyl-CoA synthetase |
| F57B9.3 | 322.1 | 0.221 | 0.31 | 0.033 |  | Translation initiation factor 4F, helicase subunit |
| F35H10.5 | 457.5 | 0.173 | 0.31 | 0.017 |  |  |
| K08D10.10 | 485.2 | 0.141 | 0.30 | 0.032 |  | 7-transmembrane receptor |
| C54D1.4 | 475.8 | 0.19 | 0.29 | 0.032 | *alh-10* | Aldehyde dehydrogenase |
| T05B11.1 | 482.3 | 0.19 | 0.28 | 0.032 |  |  |
| F17C8.5 | 497.1 | 0.185 | 0.25 | 0.040 | *twk-6* | Tandem pore domain K+ channel |
| C03G6.5 | 1.150 | 0.176 | 0.25 | 0.047 |  | Predicted secreted cysteine rich protein found only in *C. elegans* |
| F22F4.3 | 789.8 | 0.266 | 0.23 | 0.032 |  |  |

| Y44E3A.1 | 680.7 | 0.284 | 0.22 | 0.043 |  |  |
| --- | --- | --- | --- | --- | --- | --- |
| C14C11.5 | 525.2 | 0.313 | 0.17 | 0.032 | *srx-117* | 7-transmembrane receptor |
