# Supplementary material for: Gene expression profiling to characterize sediment toxicity – a pilot study using Caenorhabditis elegans whole genome microarrays
Source: BMC Genomics. 2009 Apr 14;10:160. doi: 10.1186/1471-2164-10-160 (PMC2674462; doi:10.1186/1471-2164-10-160)
Supplement: Additional file 2 — Differentially regulated genes due to the exposure to the Rhine sediment. Significantly changing transcripts in C. elegans exposed to the Rhine (R) sediment [ANOVA, p < 0.05 without multiple sample correction, fold-change to reference sediment Danube (D) > 1.4 (up-regulated) or < 0.7 (down-regulated)]. [file 1471-2164-10-160-S2.doc]

### Additional file 2 – Differentially regulated genes due to the exposure to the Rhine sediment

Significantly changing transcripts in *C. elegans* exposed to the Rhine (R) sediment [ANOVA, p<0.05 without multiple sample correction, fold-change to reference sediment Danube (D) > 1.4 (up-regulated) or < 0.7 (down-regulated)].

| **WormBASE ID** | **Rhine** | | **Fold-change over control** | **p -value** | **GCG name** | **Description** |
| --- | --- | --- | --- | --- | --- | --- |
|  | **Mean** | **SE** |  |  |  |  |
| UP-REGULATED GENES | | | | | | |
| C29E6.1 | 5.699 | 2.069 | 10.20 | 0.050 | *let-653* |  |
| K06B4.5 | 2.229 | 0.769 | 9.46 | 0.047 |  | Nuclear hormone receptor |
| Y80D3A.6 | 2.826 | 0.372 | 5.91 | 0.043 | *cyp-42A1* | Cytochrome P450 CYP4/CYP19/CYP26 subfamilies |
| F44F1.1 | 3.719 | 0.455 | 5.10 | 0.046 |  | Cytosolic Ca2+-dependent cysteine protease (calpain), large subunit (EF-Hand protein superfamily) |
| T19E7.3 | 3.135 | 0.953 | 4.91 | 0.008 | *bec-1* | Beclin-like protein |
| ZK1010.6 | 2.615 | 0.611 | 4.85 | 0.039 |  | Unnamed protein |
| F18E3.7a | 743.5 | 0.127 | 4.72 | 0.045 |  | D-aspartate oxidase |
| M7.13 | 7.323 | 2.907 | 4.60 | 0.030 | *str-3* | 7-transmembrane olfactory receptor |
| T02D1.1 | 1.421 | 0.267 | 4.52 | 0.042 |  |  |
| F40H7.7 | 2.347 | 0.673 | 4.22 | 0.006 | *srx-104* | 7-transmembrane receptor |
| F47B8.6 | 2.018 | 0.864 | 4.19 | 0.019 |  | Unnamed protein |
| Y57G11C.24c | 3.269 | 0.566 | 4.05 | 0.001 | *eps-8* |  |
| F32D8.9 | 4.393 | 1.322 | 4.03 | 0.002 | *spp-16* |  |
| F46F5.6 | 1.109 | 0.248 | 3.96 | 0.044 |  | Unnamed protein |
| F58A4.6 | 3.602 | 0.68 | 3.79 | 0.022 |  | Unnamed protein |
| Y73B3A.4 | 1.911 | 0.718 | 3.71 | 0.006 |  | Unnamed protein |
| Y71F9AL.2 | 3.257 | 0.689 | 3.71 | 0.039 |  | Casein kinase (serine/threonine/tyrosine protein kinase) |
| T02G5.11 | 2.109 | 0.528 | 3.70 | 0.032 |  |  |
| Y43C5B.2 | 1.819 | 0.627 | 3.57 | 0.034 |  | Protein tyrosine kinase |
| F44F1.5 | 3.279 | 0.51 | 3.50 | 0.046 |  | Uncharacterized coiled-coil containing protein |
| F35F10.1 | 2.184 | 0.792 | 3.38 | 0.004 |  | Predicted peptide:N-glycanase |
| R13G10.4 | 2.299 | 0.473 | 3.36 | 0.007 |  | Predicted membrane protein, contains two CBS domains |
| Y73B3A.15 | 2.432 | 0.679 | 3.33 | 0.001 |  |  |
| F41B5.10 | 2.084 | 1.113 | 3.22 | 0.027 |  | Hormone receptors |
| F41D3.7 | 4.119 | 1.049 | 3.22 | 0.035 |  |  |
| F54D1.2 | 3.813 | 0.593 | 3.18 | 0.014 | *col-127* | Collagens (type IV and type XIII), and related proteins |
| W03F11.6a | 3.041 | 1.091 | 3.17 | 0.023 |  |  |
| Y58A7A.4 | 2.632 | 0.694 | 3.16 | 0.031 |  | Unnamed protein |
| Y71H2AR.2 | 1.946 | 0.817 | 3.03 | 0.032 |  | Cysteine proteinase Cathepsin L |
| K10D11.5 | 2.676 | 0.889 | 3.02 | 0.010 |  | Uncharacterized protein |
| Y71D11A.1 | 2.365 | 0.396 | 2.96 | 0.007 |  | Cadherin repeats |
| H22K11.2 | 2.494 | 1.2 | 2.95 | 0.038 |  | Beta-galactosidase |
| C54G7.3b | 1.679 | 0.263 | 2.94 | 0.016 | *lgx-1* |  |
| Y102A5C.3 | 2.671 | 0.843 | 2.93 | 0.009 |  | Uncharacterized protein |
| R05A10.2 | 1.498 | 0.0399 | 2.93 | 0.045 |  |  |
| Y54F10BM.1 | 2.035 | 0.599 | 2.91 | 0.008 |  | Serine/threonine protein phosphatase |
| T10B11.6 | 1.971 | 0.6 | 2.91 | 0.019 |  | Uncharacterized conserved protein |
| C55A6.10 | 2.352 | 0.448 | 2.90 | 0.011 |  | Uncharacterized conserved protein |
| R06A4.8 | 2.098 | 0.392 | 2.89 | 0.019 |  | Alpha amylase |
| W03H9.3 | 2.385 | 0.774 | 2.87 | 0.026 |  | Uncharacterized protein |
| VC5.5 | 2.158 | 0.577 | 2.84 | 0.050 | *nhr-135* | Hormone receptors |
| F57G12.2 | 3.314 | 0.46 | 2.83 | 0.021 |  |  |
| C01G8.5b | 3.058 | 0.453 | 2.82 | 0.003 | *erm-1* | Radixin, moesin and related proteins of the ERM family |
| R08F11.1 | 1.870 | 0.192 | 2.80 | 0.015 |  | Predicted bile acid beta-glucosidase |
| Y54C5B.1 | 1.982 | 0.623 | 2.79 | 0.045 |  | DNA-binding centromere protein B (CENP-B) |
| K04C2.5 | 3.320 | 0.82 | 2.74 | 0.039 |  |  |
| F25H5.1b | 2.288 | 0.398 | 2.72 | 0.026 | *tag-15* | LIM domain |
| F45E12.6 | 3.065 | 0.253 | 2.71 | 0.034 |  | Predicted TR:Q20433 AAA68793.1 |
| F43E2.1 | 3.136 | 0.397 | 2.69 | 0.024 |  | S-M checkpoint control protein CID1 and related nucleotidyltransferases |

| M02F4.3 | 2.197 | 0.106 | 2.69 | 0.003 |  | Predicted membrane protein, contains two CBS domains |
| --- | --- | --- | --- | --- | --- | --- |
| F39G3.6 | 1.049 | 0.295 | 2.68 | 0.019 |  | Integral membrane O-acyltransferase |
| F55G1.6 | 9.996 | 0.462 | 2.67 | 0.017 |  | Unnamed protein |
| M02D8.2 | 5.516 | 0.858 | 2.67 | 0.034 |  |  |
| C06B3.1 | 3.093 | 1.229 | 2.66 | 0.045 |  | 7-transmembrane olfactory receptor |
| R10E9.2 | 3.359 | 0.598 | 2.63 | 0.022 |  | Unnamed protein |
| F11H8.4b | 2.648 | 0.549 | 2.63 | 0.027 | *cyk-1* | RhoA GTPase effector DIA/Diaphanous |
| R151.1 | 3.857 | 0.472 | 2.63 | 0.003 |  |  |
| F55D1.1 | 3.768 | 0.53 | 2.60 | 0.006 |  |  |
| Y71D11A.5 | 2.623 | 0.462 | 2.59 | 0.021 |  | Ligand-gated ion channel |
| T05F1.8 | 4.566 | 0.454 | 2.56 | 0.004 |  | Mitochondrial phosphate carrier protein |
| ZK816.3 | 2.225 | 0.483 | 2.54 | 0.023 |  | Predicted TR:Q23609 AAA82324.1 |
| F56C3.5 | 2.403 | 0.435 | 2.52 | 0.003 |  |  |
| F09G8.3 | 2.144 | 0.274 | 2.51 | 0.014 |  | Mitochondrial/chloroplast ribosomal protein S9 |
| T09B4.1 | 6.849 | 0.449 | 2.48 | 0.030 |  | Predicted Dolichyl-phosphate-mannose-protein mannosyltransferase |
| ZK829.2 | 2.089 | 0.315 | 2.48 | 0.013 | *hdl-1* | Aromatic-L-amino-acid/L-histidine decarboxylase |
| K02A2.2 | 1.402 | 0.214 | 2.48 | 0.010 | *srd-55* | Chemoreceptor/7TM receptor |
| C50F4.1 | 2.567 | 0.136 | 2.48 | 0.001 |  |  |
| R01E6.6 | 3.912 | 0.495 | 2.45 | 0.030 |  | Unnamed protein |
| M01F1.3 | 4.771 | 0.374 | 2.44 | 0.019 |  | Lipoate synthase |
| H14E04.4 | 3.173 | 0.454 | 2.44 | 0.006 |  |  |
| C05D11.13 | 1.906 | 0.541 | 2.44 | 0.028 |  | Zn-finger protein and coiled-coil protein |
| C04G2.1 | 1.811 | 0.415 | 2.43 | 0.015 |  | Uncharacterized protein with conserved cysteine |
| Y62H9A.11 | 1.478 | 0.52 | 2.43 | 0.041 |  |  |
| F01D4.3 | 1.725 | 0.356 | 2.42 | 0.027 |  | Protein tyrosine kinase |
| ZK546.14 | 1.963 | 0.364 | 2.42 | 0.043 |  |  |
| W05H5.4 | 2.490 | 0.365 | 2.42 | 0.011 | *srh-27* | Predicted olfactory G-protein coupled receptor |
| R102.4a | 3.966 | 0.503 | 2.41 | 0.030 |  | Threonine aldolase |
| Y39G10AR.17 | 2.049 | 0.382 | 2.41 | 0.038 |  |  |
| Y43E12A.2 | 2.544 | 0.372 | 2.41 | 0.003 |  | Predicted Zn-finger protein |
| Y37A1B.1 | 2.840 | 0.182 | 2.40 | 0.001 | *lst-3* | Predicted DNA-binding protein, contains SAP domain |
| C18E3.4 | 1.861 | 0.182 | 2.40 | 0.007 |  |  |
| K04F1.10 | 2.478 | 0.129 | 2.40 | 0.020 |  | Predicted receptor |
| Y116F11B.3 | 3.365 | 0.225 | 2.39 | 0.012 | *pcp-4* | Hydrolytic enzymes of the alpha/beta hydrolase fold |
| Y54E10BR.2 | 3.815 | 0.548 | 2.36 | 0.026 |  | GTP-binding ADP-ribosylation factor-like protein yARL3 |
| R07B5.7 | 4.137 | 0.366 | 2.35 | 0.027 |  |  |
| R11G11.7 | 2.245 | 0.29 | 2.35 | 0.049 | *pqn-60* | Predicted alpha-helical protein |
| K07B1.5a | 2.450 | 0.4 | 2.35 | 0.025 | *acl-14* | Lysophosphatidic acid acyltransferase LPAAT and related acyltransferases |
| F13H8.10c | 1.606 | 0.619 | 2.34 | 0.034 | *bpl-1* |  |
| R02E12.2 | 3.649 | 0.633 | 2.32 | 0.034 |  | Conserved protein Mo25 |
| F57A8.3 | 1.484 | 0.216 | 2.32 | 0.007 | *str-118* | 7-transmembrane olfactory receptor |
| Y119D3A.4 | 1.128 | 0.293 | 2.31 | 0.035 |  | Uncharacterized protein |
| Y82E9BR.19 | 2.234 | 0.419 | 2.30 | 0.030 |  |  |
| R01H2.2 | 3.899 | 0.556 | 2.29 | 0.022 |  | Peptidases |
| F14F4.3b | 3.208 | 0.294 | 2.29 | 0.023 | *mrp-5* | Multidrug resistance-associated protein/mitoxantrone resistance protein, ABC superfamily |
| K07A12.7 | 4.135 | 0.856 | 2.28 | 0.036 |  | Mitochondrial/choloroplast ribosomal protein S15 |
| F25H5.8 | 2.290 | 0.646 | 2.28 | 0.040 |  | Stress responsive protein |
| C30A5.7a | 2.096 | 0.321 | 2.28 | 0.013 | *unc-86* | Transcription factor ACJ6/BRN-3, contains POU and HOX domains |
| T01C3.8 | 2.438 | 0.23 | 2.27 | 0.033 |  |  |
| H23N18.3 | 5.035 | 0.25 | 2.26 | 0.029 | *ugt-8* | UDP-glucuronosyl and UDP-glucosyl transferase |
| F13G3.11 | 1.751 | 0.206 | 2.26 | 0.011 |  | Mitochondrial/chloroplast ribosomal protein L13 |
| R09A8.1 | 3.464 | 0.585 | 2.26 | 0.014 |  | Unnamed protein |
| Y75B8A.32 | 2.915 | 0.345 | 2.26 | 0.036 |  | Predicted DNA-binding protein |
| T25D3.4 | 2.836 | 0.456 | 2.26 | 0.029 |  | Predicted transporter ADD1 (major facilitator superfamily) |
| T05H4.4 | 2.407 | 0.433 | 2.25 | 0.050 |  | NADH-cytochrome b-5 reductase |
| R11D1.7 | 4.042 | 0.376 | 2.25 | 0.012 |  |  |
| Y46G5A.26 | 2.612 | 0.329 | 2.25 | 0.016 |  | GABA receptor |
| ZK39.8 | 1.147 | 0.322 | 2.24 | 0.035 |  | C-type lectin |

| C36E8.1 | 2.908 | 0.127 | 2.23 | 0.032 |  | RNA polymerase I transcription factor |
| --- | --- | --- | --- | --- | --- | --- |
| C16D2.1 | 1.900 | 0.148 | 2.23 | 0.006 |  |  |
| H24K24.3b | 3.060 | 0.515 | 2.22 | 0.026 |  |  |
| C09F12.2 | 4.958 | 0.308 | 2.22 | 0.041 |  |  |
| C52B11.3 | 4.463 | 0.431 | 2.22 | 0.016 |  | 7 transmembrane receptor |
| F49E11.1a | 3.133 | 0.318 | 2.21 | 0.005 | *mbk-2* | Dual-specificity tyrosine-phosphorylation regulated kinase |
| F10G7.10a | 1.459 | 0.1 | 2.21 | 0.002 |  | Predicted ubiquitin-protein ligase of the N-recognin family |
| F41D3.11 | 4.557 | 0.484 | 2.20 | 0.011 |  | Glycosyltranferase |
| T07D10.5 | 2.951 | 0.324 | 2.19 | 0.028 |  | C-type lectin Predicted TR:O02302 CAB04714.1 |
| F56A4.7 | 3.139 | 0.201 | 2.19 | 0.034 | *str-154* | 7-transmembrane olfactory receptor |
| ZC487.4 | 1.322 | 0.334 | 2.19 | 0.033 | *grl-9* | GROUND domains (extracellular cysteine-containing domain sometimes associated with HOG domains) |
| F26D2.3 | 1.744 | 0.245 | 2.18 | 0.029 |  |  |
| Y105C5A.9 | 3.295 | 0.406 | 2.18 | 0.015 |  | Unnamed protein |
| ZK40.1 | 3.594 | 0.225 | 2.17 | 0.002 | *acl-9* | Lysophosphatidic acid acyltransferase LPAAT and related acyltransferases |
| C04E12.5 | 1.466 | 0.472 | 2.15 | 0.032 |  | Predicted peptide:N-glycanase |
| Y49F6C.4 | 1.452 | 0.519 | 2.15 | 0.034 |  | Uncharacterized protein, contains BTB/POZ domain |
| T23D5.11 | 1.692 | 0.337 | 2.15 | 0.032 | *str-8* | 7-transmembrane olfactory receptor |
| Y68A4A.9 | 9.482 | 0.358 | 2.14 | 0.015 |  | No Significant Match |
| T27E4.6 | 3.042 | 0.17 | 2.11 | 0.020 |  | Predicted acyltransferase |
| T12B5.1 | 3.653 | 0.406 | 2.11 | 0.031 |  | Uncharacterized protein |
| K12H6.4 | 6.100 | 0.336 | 2.11 | 0.016 |  |  |
| ZK632.12 | 2.387 | 0.361 | 2.10 | 0.036 |  | FYVE finger containing protein |
| F59C12.1 | 2.220 | 0.143 | 2.09 | 0.025 | *cdh-9* | Cadherin repeats |
| C01F6.4 | 5.664 | 0.595 | 2.09 | 0.030 | *fem-3* |  |
| C46C2.4 | 1.205 | 0.337 | 2.09 | 0.027 |  | Hydrolytic enzymes of the alpha/beta hydrolase fold |
| F28C12.6 | 2.395 | 0.493 | 2.09 | 0.036 |  | Transposase-encoded protein linked to peptidase |
| Y111B2A.20 | 1.619 | 0.125 | 2.09 | 0.023 |  | UDP-galactose transporter related protein |
| K09A9.3 | 3.915 | 0.115 | 2.08 | 0.025 | *ent-2* | Nucleoside transporter |
| F39G3.7 | 1.070 | 0.233 | 2.08 | 0.016 | *prx-6* | Peroxisome assembly factor 2 containing the AAA+-type ATPase domain |
| C08F11.7 | 1.451 | 0.145 | 2.07 | 0.019 |  | Uncharacterized protein |
| T27C4.2 | 4.656 | 0.481 | 2.07 | 0.021 |  |  |
| F52H2.4 | 5.298 | 0.566 | 2.06 | 0.040 |  | Na+:iodide/myo-inositol/multivitamin symporters |
| F17H10.1 | 1.446 | 0.202 | 2.06 | 0.032 |  | Uncharacterized conserved protein |
| T05A8.4 | 12.616 | 0.236 | 2.06 | 0.030 |  | M13 family peptidase |
| C16A11.4 | 2.729 | 0.216 | 2.04 | 0.009 |  | Uncharacterized protein |
| F34H10.2 | 3.296 | 0.145 | 2.03 | 0.038 |  |  |
| Y54G2A.21 | 1.467 | 0.215 | 2.03 | 0.048 |  |  |
| T27A10.6 | 3.438 | 0.463 | 2.03 | 0.013 |  |  |
| F41G3.5 | 2.941 | 0.415 | 2.03 | 0.049 |  | Casein kinase (serine/threonine/tyrosine protein kinase) |
| T05F1.12 | 2.212 | 0.136 | 2.03 | 0.024 |  |  |
| Y39G10AL.3 | 1.468 | 0.132 | 2.02 | 0.040 | *cdk-7* | Cdk activating kinase (CAK)/RNA polymerase II transcription initiation/nucleotide excision repair factor TFIIH/TFIIK, kinase subunit CDK7 |
| T12B3.4 | 2.118 | 0.172 | 2.01 | 0.043 |  | Cell cycle-associated protein |
| T05F1.5 | 5.716 | 0.157 | 2.01 | 0.015 |  | Unnamed protein |
| Y43F8A.5 | 1.502 | 0.211 | 2.01 | 0.031 |  | Predicted transporter ADD1 (major facilitator superfamily) |
| F49D11.9 | 2.032 | 0.363 | 2.00 | 0.047 | *tag-296* | Uncharacterized conserved protein |
| T06G6.11 | 1.899 | 0.255 | 1.99 | 0.021 |  | Uncharacterized conserved protein, contains WD40 repeats and FYVE domains |
| F58A6.11 | 2.753 | 0.364 | 1.99 | 0.026 | *srb-13* | Sra family integral membrane protein |
| T12C9.7 | 2.305 | 0.27 | 1.99 | 0.008 |  |  |
| T01H10.7 | 20.935 | 0.348 | 1.99 | 0.025 |  | No Significant Match |
| C53A5.3 | 1.999 | 0.161 | 1.99 | 0.001 | *hda-1* | Histone deacetylase complex, catalytic component RPD3 |
| B0222.6 | 2.560 | 0.282 | 1.99 | 0.048 | *col-144* | Collagens (type IV and type XIII), and related proteins |
| F55G1.5 | 2.399 | 0.275 | 1.99 | 0.019 |  | Mitochondrial solute carrier protein |
| ZK1225.4 | 5.432 | 0.193 | 1.96 | 0.006 |  | Splicing factor RNPS1, SR protein superfamily |
| F31A9.1 | 1.996 | 0.35 | 1.96 | 0.020 |  | Unnamed protein |
| W06F12.1a | 4.923 | 0.281 | 1.96 | 0.015 | *lit-1* | Nemo-like MAPK-related serine/threonine protein kinase |
| C53B7.3 | 7.646 | 0.144 | 1.95 | 0.049 |  |  |
| F56A12.1 | 4.674 | 0.378 | 1.95 | 0.018 | *unc-39* | Transcription factor SIX and related HOX domain proteins |
| ZC449.6 | 2.496 | 0.208 | 1.94 | 0.037 |  | C-3 sterol dehydrogenase/3-beta-hydroxysteroid dehydrogenase and related dehydrogenases |

| C06B8.3 | 1.237 | 0.232 | 1.93 | 0.003 |  | Hormone receptors |
| --- | --- | --- | --- | --- | --- | --- |
| C18A11.1 | 5.265 | 0.169 | 1.93 | 0.026 |  | Unnamed protein |
| F48D6.4c | 2.666 | 0.304 | 1.93 | 0.034 |  | Unnamed protein |
| Y51H1A.3b | 1.631 | 0.352 | 1.93 | 0.024 |  | NADH:ubiquinone oxidoreductase, NDUFB8/ASHI subunit |
| H05C05.2b | 2.059 | 0.417 | 1.93 | 0.029 |  |  |
| T24A6.15 | 1.150 | 0.403 | 1.91 | 0.034 | *grl-28* | GROUND domains (extracellular cysteine-containing domain sometimes associated with HOG domains) |
| W04G5.7 | 2.283 | 0.292 | 1.91 | 0.030 |  | Uncharacterized protein |
| ZC196.9 | 3.807 | 0.216 | 1.91 | 0.007 |  | Membrane protein |
| F43G6.8 | 1.990 | 0.238 | 1.91 | 0.032 |  | Predicted E3 ubiquitin ligase |
| T10B10.8 | 3.901 | 0.367 | 1.90 | 0.027 |  | Glycosyl transferase, family 8 - glycogenin |
| F36H5.1 | 20.259 | 0.0973 | 1.90 | 0.042 |  | Uncharacterized protein, contains BTB/POZ domain |
| K07A1.11 | 2.417 | 0.189 | 1.90 | 0.020 | *rba-1* | Nucleosome remodeling factor, subunit CAF1/NURF55/MSI1 |
| Y53H1C.1 | 2.618 | 0.284 | 1.89 | 0.013 | *aat-9* |  |
| W05B2.4 | 3.061 | 0.265 | 1.88 | 0.025 |  | Dyneins, heavy chain |
| T01G9.6a | 2.461 | 0.212 | 1.87 | 0.018 | *kin-10* | Casein kinase II, beta subunit |
| F43D9.3 | 2.656 | 0.337 | 1.86 | 0.036 |  | Vesicle trafficking protein Sly1 (Sec1 family) |
| Y53F4B.17 | 1.667 | 0.225 | 1.86 | 0.037 |  | Uncharacterized protein |
| T09A5.7 | 1.516 | 0.126 | 1.86 | 0.044 |  | Uncharacterized conserved protein |
| F52B5.1 | 2.443 | 0.23 | 1.85 | 0.013 | *abts-1* | Na+-independent Cl/HCO3 exchanger AE1 and related transporters (SLC4 family) |
| ZK858.7 | 4.053 | 0.201 | 1.85 | 0.008 |  | tRNA(1-methyladenosine) methyltransferase, subunit GCD10 |
| T01D1.5 | 2.183 | 0.221 | 1.85 | 0.022 |  |  |
| R04D3.3 | 2.019 | 0.178 | 1.85 | 0.050 |  | Unnamed protein |
| K03E5.2 | 2.049 | 0.188 | 1.84 | 0.016 |  |  |
| C47B2.7a | 2.299 | 0.186 | 1.84 | 0.010 |  | Selenocysteine-specific elongation factor |
| F43G6.3 | 3.471 | 0.217 | 1.83 | 0.037 |  | No Significant Match |
| F01F1.7 | 1.632 | 0.265 | 1.83 | 0.015 |  | U5 snRNP-like RNA helicase subunit |
| R04A9.3 | 2.526 | 0.215 | 1.82 | 0.015 |  |  |
| H12D21.7 | 2.820 | 0.273 | 1.82 | 0.043 |  | Mercaptopyruvate sulfurtransferase/thiosulfate sulfurtransferase |
| Y66D12A.12 | 5.568 | 0.35 | 1.82 | 0.039 |  | Zn-finger |
| Y9D1A.1 | 2.190 | 0.16 | 1.81 | 0.036 |  | No Significant Match |
| T04C12.2 | 1.258 | 0.276 | 1.80 | 0.026 | *srh-75* |  |
| F09C12.7 | 17.738 | 0.273 | 1.79 | 0.020 | *msp-74* |  |
| M01G12.11 | 2.329 | 0.217 | 1.79 | 0.040 |  |  |
| F29G6.3b | 8.655 | 0.143 | 1.79 | 0.008 |  | Unnamed protein |
| K08C9.2 | 4.634 | 0.188 | 1.79 | 0.042 |  | Unnamed protein |
| C01B10.3 | 2.318 | 0.2 | 1.78 | 0.008 |  | Inositol polyphosphate 5-phosphatase, type I |
| Y53C12B.3b | 2.595 | 0.173 | 1.78 | 0.032 | *nos-3* | Unnamed protein |
| K01A2.5 | 1.586 | 0.129 | 1.78 | 0.022 |  | Predicted hydrolase |
| C25A8.4 | 3.089 | 0.171 | 1.78 | 0.049 |  | Chitinase |
| Y17G7B.8 | 1.924 | 0.154 | 1.77 | 0.031 |  | Uncharacterized protein |
| F44C8.9 | 3.058 | 0.224 | 1.76 | 0.026 |  |  |
| W07A8.5 | 2.505 | 0.228 | 1.76 | 0.047 | *srxa-6* | Uncharacterized protein |
| Y69A2AR.21 | 1.863 | 0.142 | 1.76 | 0.015 |  | Uncharacterized conserved protein |
| F28E10.2 | 2.956 | 0.178 | 1.75 | 0.028 |  |  |
| K07C6.5 | 2.266 | 0.059 | 1.75 | 0.039 | *cyp-35A5* | Cytochrome P450 CYP2 subfamily |
| C24F3.5 | 1.270 | 0.206 | 1.74 | 0.029 | *abt-1* | Lipid exporter ABCA1 and related proteins, ABC superfamily |
| F32D1.4 | 1.361 | 0.197 | 1.74 | 0.005 | *grl-13* | GROUND domains (extracellular cysteine-containing domain sometimes associated with HOG domains) |
| B0454.3 | 1.306 | 0.259 | 1.74 | 0.027 | *sri-28* | Predicted olfactory G-protein coupled receptor |
| K10D6.1 | 3.164 | 0.244 | 1.74 | 0.039 |  | Ligand-gated ion channel |
| F14D7.4 | 984 | 0.136 | 1.72 | 0.046 |  |  |
| F53C3.5 | 1.261 | 0.21 | 1.72 | 0.047 |  | Uncharacterized protein, contains CX module |
| K07B1.4 | 3.369 | 0.209 | 1.71 | 0.027 |  |  |
| F44F4.7 | 1.335 | 0.113 | 1.71 | 0.012 | *sra-12* | Sra family integral membrane protein |
| T06A1.3 | 1.858 | 0.1 | 1.70 | 0.001 |  |  |
| C54G6.1b | 4.549 | 0.28 | 1.70 | 0.027 |  | Unnamed protein |
| C29F7.7 | 1.344 | 0.237 | 1.70 | 0.029 |  | Unnamed protein |
| W09H1.1 | 2.157 | 0.096 | 1.70 | 0.009 |  |  |
| K11G12.1 | 2.191 | 0.153 | 1.69 | 0.023 | *nas-11* | Meprin A metalloprotease |

| F53B2.1 | 11.049 | 0.139 | 1.69 | 0.038 |  |  |
| --- | --- | --- | --- | --- | --- | --- |
| T05H4.11 | 3.493 | 0.268 | 1.68 | 0.027 |  |  |
| F30A10.3 | 1.563 | 0.156 | 1.67 | 0.030 |  | Inositol polyphosphate multikinase, component of the ARGR transcription regulatory complex |
| K10B3.6 | 2.555 | 0.141 | 1.65 | 0.024 |  | Predicted starch-binding protein |
| F55C12.2 | 1.820 | 0.0926 | 1.65 | 0.013 |  |  |
| Y40C5A.4a | 2.964 | 0.219 | 1.65 | 0.019 |  |  |
| T26E4.7 | 1.951 | 0.126 | 1.64 | 0.028 |  | Glycosyltranferase |
| C17E7.12 | 1.644 | 0.186 | 1.64 | 0.046 |  |  |
| B0414.3 | 5.717 | 0.0791 | 1.64 | 0.015 | *hil-5* | Histone H1 |
| F32E10.6 | 1.100 | 0.333 | 1.64 | 0.048 |  | Heterochromatin-associated protein HP1 and related CHROMO domain proteins |
| R04B5.7 | 2.300 | 0.291 | 1.63 | 0.035 |  |  |
| R02D3.5 | 2.727 | 0.0926 | 1.63 | 0.033 |  | Protein farnesyltransferase, alpha subunit/protein geranylgeranyltransferase type I, alpha subunit |
| C31H1.3 | 7.152 | 0.289 | 1.62 | 0.048 |  |  |
| T12D8.8 | 5.289 | 0.138 | 1.62 | 0.019 |  | Hsp70-interacting protein Hip/Transient component of progesterone receptor complexes and an Hsp70-binding protein |
| T28A11.2 | 2.432 | 0.161 | 1.62 | 0.019 |  | Predicted secreted cysteine rich protein found only in C.elegans |
| F35G12.5 | 6.479 | 0.217 | 1.62 | 0.016 |  |  |
| M03F4.7 | 2.324 | 0.073 | 1.62 | 0.031 |  | Reticulocalbin, calumenin, DNA supercoiling factor, and related Ca2+-binding proteins of the CREC family (EF-Hand protein superfamily) |
| F48A11.5 | 2.658 | 0.119 | 1.61 | 0.010 |  |  |
| K07F5.6 | 7.700 | 0.153 | 1.61 | 0.030 |  | Protein tyrosine phosphatase |
| K10D2.3 | 3.461 | 0.196 | 1.61 | 0.020 |  | S-M checkpoint control protein CID1 and related nucleotidyltransferases |
| C54D1.2 | 3.403 | 0.183 | 1.60 | 0.042 |  | C-type lectin |
| C50E10.4 | 8.343 | 0.0709 | 1.60 | 0.047 | *sop-2* | Unnamed protein |
| Y50E8A.9 | 3.913 | 0.116 | 1.59 | 0.032 |  | Phospholipid scramblase |
| R05H10.6 | 2.806 | 0.131 | 1.59 | 0.022 | *cdh-7* | Cadherin repeats |
| C06A8.9 | 1.040 | 0.177 | 1.59 | 0.010 | *glr-4* | Glutamate-gated kainate-type ion channel receptor subunit GluR5 and related subunits |
| H02F09.3 | 1.151 | 0.0819 | 1.57 | 0.017 |  | Unnamed protein |
| F47B8.3 | 2.241 | 0.0676 | 1.57 | 0.029 |  | Glutaredoxin-related protein |
| Y54E5B.2 | 2.783 | 0.157 | 1.57 | 0.047 |  | WD-repeat protein WDR6, WD repeat superfamily |
| F58G11.4 | 2.575 | 0.151 | 1.57 | 0.037 |  | Predicted transporter/transmembrane protein |
| C43H8.1 | 1.999 | 0.148 | 1.56 | 0.030 |  | Uncharacterized conserved protein |
| Y17G7B.18b | 1.434 | 0.252 | 1.56 | 0.032 |  | Predicted methyltransferase |
| C13C4.3 | 950.7 | 0.0849 | 1.56 | 0.003 | *nhr-136* | Nuclear hormone receptor |
| C42C1.2 | 1.450 | 0.164 | 1.56 | 0.022 |  | Serine/threonine phosphatase |
| T02E1.7 | 5.132 | 0.175 | 1.55 | 0.035 |  | Putative cargo transport protein ERV29 |
| T09E8.1e | 8.090 | 0.215 | 1.54 | 0.044 |  |  |
| T22E5.1 | 2.211 | 0.0999 | 1.54 | 0.026 |  |  |
| Y39A1C.4 | 2.969 | 0.191 | 1.54 | 0.032 |  | Unnamed protein |
| Y40A1A.3 | 4.353 | 0.0792 | 1.54 | 0.025 |  |  |
| ZK792.2 | 1.722 | 0.165 | 1.53 | 0.049 | *inx-8* | Innexin-type channels |
| T27B7.6 | 1.899 | 0.177 | 1.52 | 0.041 |  | Hormone receptors |
| T27A10.2 | 5.949 | 0.188 | 1.52 | 0.044 |  |  |
| F53F1.3 | 2.172 | 0.13 | 1.51 | 0.031 |  | Aldo/keto reductase family proteins |
| T28H11.6 | 3.841 | 0.264 | 1.51 | 0.030 | *ssp-11* | Uncharacterized protein, contains major sperm protein (MSP) domain |
| F53C3.12 | 838.9 | 0.183 | 1.50 | 0.035 |  | Beta, beta-carotene 15,15'-dioxygenase and related enzymes |
| M01H9.3b | 5.618 | 0.134 | 1.50 | 0.024 |  |  |
| R09D1.11 | 19.145 | 0.0782 | 1.50 | 0.048 |  | Chitinase |
| F18A11.4 | 1.550 | 0.0831 | 1.49 | 0.043 |  |  |
| W02D7.9 | 762.4 | 0.0702 | 1.49 | 0.018 |  |  |
| Y73F8A.33 | 2.960 | 0.116 | 1.49 | 0.041 |  | C2H2-type Zn-finger |
| T24D1.3 | 6.203 | 0.151 | 1.48 | 0.046 |  | Unnamed protein |
| F59G1.3 | 1.532 | 0.129 | 1.48 | 0.032 | *vps-35* | Membrane coat complex Retromer, subunit VPS35 |
| ZK909.3 | 1.319 | 0.106 | 1.47 | 0.032 |  | Predicted guanosine polyphosphate pyrophosphohydrolase/synthase |
| Y43C5A.3 | 1.897 | 0.136 | 1.46 | 0.038 |  | Confirmed TR:Q9XXF1 CAA19503.1 |
| F28H1.5 | 21.450 | 0.136 | 1.46 | 0.014 |  |  |
| T07D3.9 | 1.321 | 0.097 | 1.44 | 0.045 |  |  |
| ZK250.8 | 2.026 | 0.129 | 1.42 | 0.024 |  |  |
| F57C7.1a | 3.171 | 0.359 | 1.42 | 0.036 |  | Transcription initiation factor TFIID, subunit BDF1 and related bromodomain proteins |
| C31H1.8 | 1.527 | 0.148 | 1.42 | 0.031 |  |  |

| DOWN-REGULATED GENES | | | | | | |
| --- | --- | --- | --- | --- | --- | --- |
| DY3.5 | 1.475 | 0.102 | 0.71 | 0.048 | *pqn-26* | Glutaredoxin and related proteins |
| C33A12.2 | 1.806 | 0.107 | 0.71 | 0.049 |  | Confirmed TR:Q18356 CAA92786.1 |
| T13A10.8 | 1.635 | 0.0632 | 0.71 | 0.013 |  | No Significant Match |
| C25G4.10 | 12.200 | 0.309 | 0.70 | 0.027 |  | Fibronectin |
| C49C8.2 | 1.496 | 0.0676 | 0.70 | 0.026 |  |  |
| F56B6.5 | 2.114 | 0.0956 | 0.70 | 0.049 | *uvt-6* | 7 transmembrane receptor |
| F13D12.3 | 1.039 | 0.0915 | 0.70 | 0.043 |  |  |
| F49E7.1b | 1.601 | 0.0635 | 0.70 | 0.003 | *rme-6* | Vacuolar assembly/sorting protein VPS9 |
| F58H7.7 | 882.7 | 0.0896 | 0.70 | 0.013 |  | F-box domain |
| B0496.3b | 1.088 | 0.0854 | 0.69 | 0.022 |  | Unnamed protein |
| C16H3.3 | 2.028 | 0.0911 | 0.69 | 0.046 |  |  |
| Y54E10A.18 | 2.469 | 0.0556 | 0.69 | 0.038 |  | Predicted E3 ubiquitin ligase |
| F54C1.1 | 51.692 | 0.11 | 0.69 | 0.042 |  | UDP-glucuronosyl and UDP-glucosyl transferase |
| C14C10.7 | 904.2 | 0.0534 | 0.69 | 0.019 |  | Uncharacterized protein with conserved cysteine |
| C32C4.3 | 3.293 | 0.0598 | 0.69 | 0.029 |  | Voltage-gated K+ channel KCNB/KCNC |
| C34E11.1 | 4.348 | 0.0703 | 0.69 | 0.049 | *rsd-3* | Predicted equilibrative nucleoside transporter protein |
| C03B1.13 | 1.541 | 0.0779 | 0.69 | 0.048 |  | Predicted transporter (major facilitator superfamily) |
| C49H3.12 | 2.388 | 0.0986 | 0.68 | 0.046 |  |  |
| ZK1067.2 | 1.624 | 0.0408 | 0.68 | 0.008 |  | Helicases |
| C07D10.5 | 772.4 | 0.0845 | 0.68 | 0.027 |  |  |
| Y76B12C.9 | 1.343 | 0.0907 | 0.68 | 0.029 |  |  |
| D2021.8 | 2.750 | 0.0754 | 0.68 | 0.027 |  | Ankyrin repeat and DHHC-type Zn-finger domain containing proteins |
| C37H5.12 | 3.257 | 0.0805 | 0.68 | 0.037 |  | No Significant Match |
| Y71H10A.1b | 10.721 | 0.0923 | 0.67 | 0.041 |  |  |
| F59F5.3 | 8.929 | 0.0788 | 0.67 | 0.029 |  | Fibroblast/platelet-derived growth factor receptor and related receptor tyrosine kinases |
| C18B12.4 | 2.016 | 0.0793 | 0.67 | 0.028 |  | Predicted E3 ubiquitin ligase |
| T25C12.3 | 1.411 | 0.0816 | 0.67 | 0.037 |  | Unnamed protein |
| F56D1.1 | 1.503 | 0.138 | 0.67 | 0.040 |  | Zn-finger |
| F41F3.6 | 2.285 | 0.0974 | 0.67 | 0.040 | *srx-74* |  |
| K11D12.6 | 41.636 | 0.0403 | 0.67 | 0.031 |  | Serine proteinase inhibitor (KU family) |
| F10G7.6 | 2.446 | 0.0906 | 0.66 | 0.045 |  | Uncharacterized protein |
| T06E8.2 | 1.970 | 0.0576 | 0.66 | 0.006 |  |  |
| Y17G7B.2a | 1.823 | 0.123 | 0.66 | 0.049 |  | Histone H3 (Lys4) methyltransferase complex, subunit CPS60/ASH2/BRE2 |
| F08F3.9 | 894 | 0.108 | 0.66 | 0.047 |  | Small nuclear RNA activating complex (SNAPc), subunit SNAP43 |
| C26D10.6 | 1.408 | 0.131 | 0.66 | 0.033 |  | Unnamed protein |
| C56E6.6 | 836.2 | 0.0784 | 0.66 | 0.040 |  | Leucine rich repeat |
| T01C8.7 | 1.322 | 0.0826 | 0.66 | 0.039 | *mec-4* | Non voltage-gated ion channels (DEG/ENaC family) |
| F49H6.8 | 81.802 | 0.102 | 0.66 | 0.035 |  | Unnamed protein |
| C01C4.1 | 1.081 | 0.141 | 0.66 | 0.045 | *nlp-1* |  |
| F15E6.3 | 1.770 | 0.0944 | 0.66 | 0.045 |  | Unnamed protein |
| T07D3.7 | 1.708 | 0.0767 | 0.65 | 0.012 | *alg-2* | Translation initiation factor 2C (eIF-2C) and related proteins |
| C08H9.1 | 801.6 | 0.0666 | 0.65 | 0.005 |  | Serine carboxypeptidases (lysosomal cathepsin A) |
| Y55D5A.5 | 1.022 | 0.106 | 0.65 | 0.028 | *daf-2* | Insulin/growth factor receptor (contains protein kinase domain) |
| F13A7.6 | 4.693 | 0.0896 | 0.65 | 0.046 |  |  |
| C15H11.5 | 1.028 | 0.0544 | 0.65 | 0.030 | *tag-338* |  |
| F57A8.5 | 53.178 | 0.133 | 0.65 | 0.047 |  | Nuclear hormone receptor |
| F44D12.7 | 4.257 | 0.132 | 0.64 | 0.017 |  | Uncharacterized protein, contains major sperm protein (MSP) domain |
| Y40D12A.3 | 1.111 | 0.148 | 0.64 | 0.028 | *srh-40* | Predicted olfactory G-protein coupled receptor |
| F40F4.1 | 840.4 | 0.134 | 0.64 | 0.036 |  | Predicted transposase |
| F48G7.4 | 3.353 | 0.104 | 0.64 | 0.044 |  | Uncharacterized protein |
| C42D8.1 | 1.703 | 0.13 | 0.64 | 0.041 |  |  |
| F33D4.3 | 2.070 | 0.15 | 0.64 | 0.047 | *flp-13* | Unnamed protein |
| R17.2 | 2.044 | 0.132 | 0.63 | 0.044 |  | Glucose-repressible alcohol dehydrogenase transcriptional effector CCR4 and related proteins |
| T23G4.1 | 2.694 | 0.0527 | 0.63 | 0.021 | *tlp-1* |  |
| C05E11.4 | 2.510 | 0.0887 | 0.63 | 0.016 | *amt-1* | Ammonia permease |
| T05A12.2 | 735.7 | 0.111 | 0.63 | 0.025 | *tre-2* | Neutral trehalase |
| Y92C3A.1 | 1.059 | 0.128 | 0.63 | 0.049 |  | No Significant Match |

| ZC84.5 | 1.362 | 0.137 | 0.63 | 0.016 |  | No Significant Match |
| --- | --- | --- | --- | --- | --- | --- |
| F09F3.11 | 643.8 | 0.0829 | 0.62 | 0.012 | *srx-135* | 7-transmembrane receptor |
| C24G6.5 | 1.190 | 0.0985 | 0.62 | 0.015 | *dnj-6* | Molecular chaperone (DnaJ superfamily) |
| C29G2.3 | 1.099 | 0.0939 | 0.62 | 0.026 |  |  |
| T09E11.3 | 3.798 | 0.106 | 0.62 | 0.038 |  | Chondroitin 6-sulfotransferase and related sulfotransferases |
| C35D6.3 | 751.1 | 0.11 | 0.62 | 0.027 |  | Unnamed protein |
| F48B9.5 | 2.051 | 0.129 | 0.62 | 0.035 |  | Transcription factor PRD and related proteins, contain PAX and HOX domains |
| C31H2.3 | 2.373 | 0.0867 | 0.61 | 0.006 |  | No Significant Match |
| T05H4.5 | 817.6 | 0.169 | 0.61 | 0.044 |  | NADH-cytochrome b-5 reductase |
| T06E6.9 | 1.996 | 0.108 | 0.61 | 0.016 | *srh-265* | Predicted olfactory G-protein coupled receptor |
| T07G12.4 | 731.8 | 0.122 | 0.61 | 0.048 |  | Xanthine/uracil transporters |
| Y119D3B.21 | 1.889 | 0.0437 | 0.61 | 0.003 |  |  |
| F41E7.5 | 15.124 | 0.104 | 0.61 | 0.030 |  | collagen-like repeats Partially_confirmed TR:Q20277 CAA92128.1 |
| C50F4.13 | 17.990 | 0.107 | 0.61 | 0.031 | *his-35* | Histone 2A |
| C37A2.8a | 1.800 | 0.0773 | 0.61 | 0.018 |  | Unnamed protein |
| F08F1.7 | 1.105 | 0.11 | 0.61 | 0.022 | *tag-123* | Endosomal membrane proteins, EMP70 |
| C49C3.7 | 1.188 | 0.134 | 0.61 | 0.029 |  | Unnamed protein |
| T07A9.11 | 6.011 | 0.0985 | 0.61 | 0.037 | *rps-24* | 40S ribosomal protein S24 |
| B0523.1 | 976.9 | 0.142 | 0.60 | 0.024 | *kin-31* | Protein tyrosine kinase |
| C36A4.6 | 816 | 0.146 | 0.60 | 0.047 | *cyp-25A4* | Cytochrome P450 CYP3/CYP5/CYP6/CYP9 subfamilies |
| F55C5.6 | 1.393 | 0.106 | 0.60 | 0.026 |  | Flavin-containing amine oxidase |
| R09H10.2 | 1.656 | 0.106 | 0.60 | 0.035 |  | Predicted secreted small molecules methylase |
| Y52B11A.5 | 1.039 | 0.0666 | 0.60 | 0.023 |  | NADH:ubiquinone oxidoreductase, NDUFB8/ASHI subunit |
| Y45G12C.5 | 2.547 | 0.239 | 0.60 | 0.035 | *srt-2* | 7-transmembrane receptor |
| C30F8.4b | 698.6 | 0.0514 | 0.60 | 0.022 | *kin-32* |  |
| F20G4.1 | 590.4 | 0.115 | 0.60 | 0.036 |  | Uncharacterized conserved protein (Neuroblastoma-amplified protein) |
| C23H5.1 | 1.947 | 0.092 | 0.59 | 0.010 |  | Unnamed protein |
| R06A10.2 | 821.4 | 0.13 | 0.59 | 0.043 | *gsa-1* | G protein subunit Galphas, small G protein superfamily |
| C08E8.2 | 5.131 | 0.0782 | 0.59 | 0.033 |  | Predicted alkaloid synthase/Surface mucin Hemomucin |
| W09D6.5 | 1.414 | 0.122 | 0.59 | 0.042 |  |  |
| T02G6.4 | 1.191 | 0.0545 | 0.59 | 0.003 |  |  |
| Y73F8A.21 | 1.082 | 0.127 | 0.59 | 0.026 | *nhr-5* | Hormone receptors |
| D2085.4 | 1.791 | 0.0677 | 0.59 | 0.009 |  | E3 ubiquitin protein ligase |
| C44B12.7 | 866.2 | 0.141 | 0.59 | 0.033 |  |  |
| W02G9.3 | 1.136 | 0.101 | 0.59 | 0.049 |  | THUMP domain-containing proteins |
| F52A8.3 | 676.3 | 0.134 | 0.59 | 0.030 |  |  |
| T28F4.1 | 814 | 0.139 | 0.59 | 0.039 |  |  |
| F11A10.4 | 1.495 | 0.152 | 0.59 | 0.039 |  | Uncharacterized conserved protein |
| Y102A5C.22 | 691.6 | 0.0918 | 0.59 | 0.008 |  | DNA/RNA helicase MER3/SLH1, DEAD-box superfamily |
| C10F3.1 | 3.417 | 0.12 | 0.59 | 0.044 |  | Unnamed protein |
| F15D3.7 | 1.932 | 0.151 | 0.59 | 0.044 |  | Mitochondrial import inner membrane translocase, subunit TIM23 |
| F21H7.2 | 703.4 | 0.0683 | 0.59 | 0.013 |  |  |
| F10D7.2 | 647.6 | 0.145 | 0.58 | 0.044 |  | Permease of the major facilitator superfamily |
| F40G12.10 | 675.9 | 0.112 | 0.58 | 0.021 |  | Protein tyrosine phosphatase |
| F48C1.8 | 1.483 | 0.116 | 0.58 | 0.046 |  | Unnamed protein |
| F53B7.5 | 557.3 | 0.0764 | 0.58 | 0.041 |  |  |
| C55A6.3 | 2.196 | 0.151 | 0.58 | 0.029 |  | Predicted short chain-type dehydrogenase |
| F07C4.2 | 2.664 | 0.0932 | 0.58 | 0.031 | *clec-45* | C-type lectin |
| C46C2.1a | 21.626 | 0.152 | 0.58 | 0.029 | *wnk-1* | Serine/threonine protein kinase |
| F57F4.4 | 2.969 | 0.111 | 0.58 | 0.031 |  | Unnamed protein |
| ZC84.6 | 3.298 | 0.06 | 0.58 | 0.037 |  | Serine proteinase inhibitor (KU family) |
| C14A6.4 | 2.487 | 0.141 | 0.58 | 0.003 |  |  |
| F47G3.1 | 713.5 | 0.126 | 0.58 | 0.026 |  |  |
| F38A1.8 | 623.8 | 0.0773 | 0.57 | 0.009 |  | Signal recognition particle receptor, alpha subunit |
| K11D2.1 | 39.131 | 0.0433 | 0.57 | 0.000 |  | RCC1 domain |
| Y43B11AR.3 | 933.7 | 0.12 | 0.57 | 0.046 |  | Predicted pseudouridine synthase |
| F17H10.3 | 1.386 | 0.0843 | 0.57 | 0.005 |  | Sorting nexin protein SNX27 |
| W02B3.5 | 16.330 | 0.118 | 0.57 | 0.036 |  | Unnamed protein |

| F36A4.6 | 1.748 | 0.115 | 0.57 | 0.036 |  | No Significant Match |
| --- | --- | --- | --- | --- | --- | --- |
| T20B12.4 | 2.665 | 0.138 | 0.57 | 0.039 |  | Unnamed protein |
| B0222.2 | 891.6 | 0.139 | 0.57 | 0.019 |  | No Significant Match |
| R09H10.1 | 1.254 | 0.118 | 0.57 | 0.011 |  | Predicted secreted small molecules methylase |
| C30B5.5 | 718.5 | 0.091 | 0.57 | 0.040 |  | 7-transmembrane receptor |
| K03B8.8 | 1.108 | 0.0722 | 0.57 | 0.004 |  |  |
| C28C12.4 | 2.402 | 0.0684 | 0.57 | 0.010 |  | Predicted alpha-helical protein |
| F12F6.7 | 1.358 | 0.0849 | 0.57 | 0.011 |  | DNA polymerase delta, regulatory subunit 55 |
| F15H10.2 | 24.501 | 0.162 | 0.57 | 0.031 |  | collagen Partially_confirmed SW:P20631 CAA98258.1 |
| C48B4.6 | 1.327 | 0.117 | 0.57 | 0.016 |  | Unnamed protein |
| F28F5.4 | 952.6 | 0.105 | 0.57 | 0.046 |  | No Significant Match |
| D1044.1 | 650.6 | 0.071 | 0.57 | 0.045 |  | Predicted small molecule kinase |
| C32H11.1 | 829.8 | 0.144 | 0.57 | 0.032 |  | Uncharacterized protein |
| F13C5.3 | 598.9 | 0.122 | 0.56 | 0.018 |  |  |
| T08E11.8 | 1.166 | 0.1 | 0.56 | 0.018 |  | Uncharacterized protein |
| F29F11.6 | 1.197 | 0.122 | 0.56 | 0.023 | *gsp-1* | Serine/threonine specific protein phosphatase PP1, catalytic subunit |
| C34E10.9 | 652.6 | 0.129 | 0.56 | 0.038 |  |  |
| C42C1.7 | 886.4 | 0.115 | 0.56 | 0.044 |  | Integral membrane O-acyltransferase |
| F39E9.7 | 948.6 | 0.0629 | 0.56 | 0.015 |  | Staufen and related double-stranded-RNA-binding proteins |
| Y82E9BR.1 | 2.089 | 0.0754 | 0.56 | 0.004 |  | Unnamed protein |
| D2007.3 | 879.5 | 0.113 | 0.56 | 0.007 |  |  |
| D1044.5 | 725.6 | 0.0976 | 0.56 | 0.040 |  |  |
| C49F5.6 | 1.074 | 0.0938 | 0.55 | 0.033 |  | Uncharacterized protein |
| R13F6.2 | 5.103 | 0.124 | 0.55 | 0.042 |  | C-type lectin |
| H06O01.1 | 1.286 | 0.146 | 0.55 | 0.030 | *pdi-3* | Protein disulfide isomerase (prolyl 4-hydroxylase beta subunit) |
| Y32F6A.4 | 760.8 | 0.104 | 0.55 | 0.034 |  | Amino acid transporters |
| C35E7.5a | 1.188 | 0.0966 | 0.55 | 0.017 |  | Uncharacterized protein |
| C44B12.5 | 4.251 | 0.144 | 0.55 | 0.045 |  |  |
| F25H5.7 | 980 | 0.124 | 0.55 | 0.007 |  | Protein tyrosine phosphatase |
| Y47D3A.22 | 1.187 | 0.0362 | 0.55 | 0.002 |  | Ankyrin repeat |
| C50H2.10 | 3.491 | 0.103 | 0.55 | 0.032 |  |  |
| B0222.8 | 5.327 | 0.0595 | 0.54 | 0.015 | *col-10* | Collagens (type IV and type XIII), and related proteins |
| C35A11.2 | 913.4 | 0.0407 | 0.54 | 0.000 |  |  |
| M142.2 | 1.711 | 0.108 | 0.54 | 0.018 | *cut-6* | Cuticulin precursor |
| W08F4.7 | 1.437 | 0.17 | 0.54 | 0.049 |  |  |
| C54F6.9 | 982.6 | 0.141 | 0.54 | 0.027 |  | Nuclear hormone receptor |
| M03F4.2b | 5.739 | 0.103 | 0.54 | 0.020 | *act-4* | Actin and related proteins |
| R04F11.5 | 807.1 | 0.127 | 0.54 | 0.030 |  | Uncharacterized conserved protein |
| F15E11.3 | 9.198 | 0.0996 | 0.54 | 0.026 |  | Predicted receptor |
| C01B10.10 | 657.8 | 0.0356 | 0.54 | 0.001 |  | Carboxylesterase and related proteins |
| C30A5.6 | 574.5 | 0.0848 | 0.54 | 0.010 |  |  |
| CD4.2 | 3.645 | 0.0702 | 0.54 | 0.005 | *crn-2* | TatD-related DNase |
| F49H12.5 | 587.9 | 0.0958 | 0.54 | 0.012 |  | Uncharacterized conserved protein |
| F52H2.7 | 1.299 | 0.13 | 0.54 | 0.016 |  | Predicted Ca2+-dependent phospholipid-binding protein |
| Y51H4A.6 | 985.5 | 0.0641 | 0.53 | 0.008 |  |  |
| C05E7.3 | 738.5 | 0.169 | 0.53 | 0.045 |  |  |
| C05E11.7 | 1.468 | 0.165 | 0.53 | 0.039 |  | Unnamed protein |
| K12D12.1 | 990.8 | 0.101 | 0.53 | 0.007 |  | DNA topoisomerase type II |
| F23B12.2 | 831.2 | 0.0826 | 0.53 | 0.011 |  |  |
| Y66H1A.6 | 904.2 | 0.18 | 0.53 | 0.038 | *hum-8* |  |
| C05D11.11b | 1.496 | 0.0885 | 0.53 | 0.048 |  |  |
| C34G6.1 | 956.7 | 0.0569 | 0.53 | 0.006 |  | Uncharacterized conserved protein |
| T20B12.8 | 868.7 | 0.119 | 0.53 | 0.035 | *hmg-4* | Nucleosome-binding factor SPN, POB3 subunit |
| C53C7.1 | 701.8 | 0.131 | 0.53 | 0.036 |  |  |
| M01E5.3 | 1.369 | 0.124 | 0.53 | 0.029 |  |  |
| C24B5.4 | 1.302 | 0.108 | 0.53 | 0.010 |  | Uncharacterized conserved protein |
| B0001.8 | 822.2 | 0.114 | 0.53 | 0.016 |  | Unnamed protein |
| C44C10.8 | 822.8 | 0.127 | 0.53 | 0.039 | *hnd-1* |  |

| F20A1.7a | 1.301 | 0.167 | 0.53 | 0.035 | *twk-11* | Tandem pore domain K+ channel |
| --- | --- | --- | --- | --- | --- | --- |
| C11H1.4 | 638.7 | 0.139 | 0.53 | 0.048 | *prx-1* | AAA+-type ATPase |
| F35H10.5 | 757.5 | 0.154 | 0.53 | 0.042 |  |  |
| C50B6.6 | 625.1 | 0.115 | 0.53 | 0.050 | *srh-15* | Predicted olfactory G-protein coupled receptor |
| F13A7.10 | 893.2 | 0.131 | 0.53 | 0.018 | *gst-44* | Glutathione S-transferase |
| F57G9.4 | 946.5 | 0.132 | 0.53 | 0.046 | *sre-29* | Sre G protein-coupled chemoreceptor |
| F17H10.2 | 1.173 | 0.0568 | 0.53 | 0.000 |  | Unnamed protein |
| C46E10.2 | 648.3 | 0.153 | 0.53 | 0.035 |  | Chemoreceptor/7TM receptor |
| F25E2.4 | 746.6 | 0.105 | 0.53 | 0.020 | *ifd-2* | Nuclear envelope protein lamin, intermediate filament superfamily |
| Y119D3B.20 | 628.1 | 0.105 | 0.53 | 0.024 |  | Uncharacterized protein |
| F45B8.3 | 1.767 | 0.0449 | 0.52 | 0.000 |  |  |
| C56E10.1 | 900.1 | 0.156 | 0.52 | 0.048 |  | Nuclear hormone receptor |
| F08G5.1 | 2.055 | 0.115 | 0.52 | 0.015 |  |  |
| Y37F4.1 | 956.2 | 0.061 | 0.52 | 0.002 |  |  |
| C43D7.6 | 499.5 | 0.0687 | 0.52 | 0.006 |  | Predicted transposase |
| ZC404.12 | 697.5 | 0.138 | 0.52 | 0.027 | *srh-30* | Predicted olfactory G-protein coupled receptor |
| C02E7.7 | 6.983 | 0.101 | 0.52 | 0.021 |  | Unnamed protein |
| F31B9.1 | 956 | 0.183 | 0.52 | 0.049 |  | Unnamed protein |
| F08F3.2 | 841 | 0.146 | 0.51 | 0.034 | *acl-6* |  |
| F20A1.6 | 604.2 | 0.127 | 0.51 | 0.045 |  |  |
| F15B10.1 | 931.4 | 0.122 | 0.51 | 0.023 |  | UDP-N-acetylglucosamine transporter |
| F28F5.3c | 535.1 | 0.041 | 0.51 | 0.007 | *tag-204* |  |
| ZK892.6 | 1.733 | 0.155 | 0.51 | 0.044 |  | Unnamed protein |
| Y54G2A.15 | 475.1 | 0.127 | 0.51 | 0.011 |  | Unnamed protein |
| C42D4.13 | 1.310 | 0.143 | 0.51 | 0.021 |  |  |
| C27H2.1 | 614.3 | 0.114 | 0.51 | 0.036 |  | Predicted TR:O45267 CAB05121.1 |
| F10E9.10 | 2.165 | 0.112 | 0.51 | 0.036 |  |  |
| ZK355.1 | 930.6 | 0.142 | 0.50 | 0.045 |  | Predicted receptor |
| C02F12.4 | 764.1 | 0.109 | 0.50 | 0.012 | *tag-52* | Predicted Rho/Rac guanine nucleotide exchange factor/faciogenital dysplasia protein 3 |
| F07G6.1 | 789.6 | 0.168 | 0.50 | 0.023 | *dgn-3* | Dystroglycan |
| F15E11.2 | 9.301 | 0.107 | 0.50 | 0.032 |  | Predicted receptor |
| T26A8.3 | 938.9 | 0.236 | 0.50 | 0.041 |  | Unnamed protein |
| F01G4.5 | 1.050 | 0.135 | 0.50 | 0.029 |  | N-acetylglucosaminyltransferase complex, subunit PIG-Q/GPI1, required for phosphatidylinositol biosynthesis |
| W09D10.3 | 31.923 | 0.179 | 0.50 | 0.033 |  | Mitochondrial/chloroplast ribosomal protein L12 |
| Y47H9C.8 | 726.9 | 0.119 | 0.50 | 0.029 |  | Small secreted protein with conserved cysteines |
| C11H1.2 | 1.921 | 0.153 | 0.50 | 0.029 |  | Predicted G-protein coupled receptor |
| K04D7.1 | 8.825 | 0.0907 | 0.50 | 0.004 |  | G protein beta subunit-like protein |
| Y71F9B.2 | 472.1 | 0.117 | 0.50 | 0.014 |  | Uncharacterized conserved protein |
| T07C5.4 | 1.703 | 0.122 | 0.50 | 0.019 |  | Nuclear hormone receptor |
| ZK593.3 | 767.3 | 0.146 | 0.50 | 0.038 |  |  |
| C36C5.4 | 1.377 | 0.11 | 0.50 | 0.026 |  | Predicted secreted cysteine rich protein found only in C.elegans |
| T27C5.3 | 2.650 | 0.0643 | 0.50 | 0.001 |  |  |
| T09A5.1 | 766.5 | 0.151 | 0.50 | 0.042 | *cex-2* |  |
| C27C7.2 | 3.888 | 0.074 | 0.50 | 0.002 |  | Unnamed protein |
| F10F2.6 | 851.3 | 0.142 | 0.50 | 0.030 |  | Lectin C-type domain/CUB domain |
| T22H2.1 | 2.027 | 0.184 | 0.50 | 0.049 | *sri-12* | Predicted olfactory G-protein coupled receptor |
| Y55B1AR.2 | 546.3 | 0.0839 | 0.49 | 0.003 |  |  |
| T03D3.12 | 758.7 | 0.133 | 0.49 | 0.017 | *srj-49* | 7-transmembrane olfactory receptor |
| F33H2.6 | 1.748 | 0.119 | 0.49 | 0.015 |  | Uncharacterized conserved protein |
| F10F2.9 | 1.069 | 0.141 | 0.49 | 0.012 | *pqn-29* |  |
| K03D3.3 | 941.5 | 0.0899 | 0.49 | 0.045 |  |  |
| C01B10.5b | 1.035 | 0.176 | 0.49 | 0.038 | *hil-7* |  |
| ZK813.3 | 1.131 | 0.175 | 0.49 | 0.040 |  |  |
| M05B5.6 | 2.264 | 0.116 | 0.49 | 0.003 |  |  |
| T05A6.1 | 937.7 | 0.0447 | 0.49 | 0.000 | *cki-1* | Cyclin-dependent kinase inhibitor |
| F01D4.2 | 877.5 | 0.12 | 0.49 | 0.016 | *ugt-44* | UDP-glucuronosyl and UDP-glucosyl transferase |
| Y43D4A.6 | 1.452 | 0.221 | 0.49 | 0.029 |  | Checkpoint kinase and related serine/threonine protein kinases |
| C47E8.1 | 704.9 | 0.169 | 0.49 | 0.047 |  |  |

| B0047.4 | 618.4 | 0.123 | 0.49 | 0.042 |  | Uncharacterized protein, contains BTB/POZ domain |
| --- | --- | --- | --- | --- | --- | --- |
| T24A6.16 | 730.6 | 0.162 | 0.49 | 0.039 |  | Chondroitin 6-sulfotransferase and related sulfotransferases |
| F17E5.1a | 10.308 | 0.0409 | 0.49 | 0.008 | *lin-2* | Calcium/calmodulin-dependent serine protein kinase/membrane-associated guanylate kinase |
| Y57G11C.1 | 805.6 | 0.168 | 0.49 | 0.036 | *haf-8* | Peptide exporter, ABC superfamily |
| W09H1.6a | 1.964 | 0.0854 | 0.48 | 0.032 | *lec-1* | Galectin, galactose-binding lectin |
| ZK792.1b | 812.8 | 0.115 | 0.48 | 0.042 |  | Histidine acid phosphatase |
| Y40B1B.5 | 675.1 | 0.123 | 0.48 | 0.022 |  | Translation initiation factor eIF3, p35 subunit |
| Y116A8A.3 | 460.8 | 0.174 | 0.48 | 0.047 |  | C-type lectin |
| C03A7.12 | 872 | 0.0964 | 0.48 | 0.004 |  | UDP-glucuronosyl and UDP-glucosyl transferase |
| T08B6.6 | 626.7 | 0.161 | 0.48 | 0.036 | *str-166* | 7-transmembrane olfactory receptor |
| B0041.7a | 1.043 | 0.118 | 0.48 | 0.037 | *xnp-1* | Transcription regulator XNP/ATRX, DEAD-box superfamily |
| C50A2.2 | 879.6 | 0.169 | 0.48 | 0.031 |  | Unnamed protein |
| C14E2.5 | 567.5 | 0.17 | 0.48 | 0.048 |  |  |
| C05E4.4 | 606.3 | 0.0948 | 0.48 | 0.039 | *sri-4* | Predicted olfactory G-protein coupled receptor |
| F07C4.1 | 3.847 | 0.0812 | 0.48 | 0.009 | *str-47* | 7-transmembrane olfactory receptor |
| ZC155.3 | 1.073 | 0.0708 | 0.48 | 0.009 |  | MORC family ATPases |
| C02F12.1 | 815 | 0.176 | 0.48 | 0.042 | *tsp-17* | Tetraspanin family integral membrane protein |
| C53B4.7a | 715 | 0.128 | 0.48 | 0.013 |  | GDP-mannose 4,6 dehydratase |
| F12E12.4 | 703.6 | 0.13 | 0.48 | 0.023 |  | Uncharacterized protein, contains BTB/POZ domain |
| T08A9.4 | 1.105 | 0.105 | 0.48 | 0.014 |  | No Significant Match |
| C14A6.8 | 765.4 | 0.124 | 0.48 | 0.035 |  | Unnamed protein |
| T10C6.6b | 885.3 | 0.129 | 0.48 | 0.045 |  | Permease of the major facilitator superfamily |
| C33F10.12 | 964.1 | 0.0478 | 0.48 | 0.010 |  | Mitochondrial phosphate carrier protein |
| C27D6.6 | 472.5 | 0.0766 | 0.48 | 0.044 | *srb-5* | Sra family integral membrane protein |
| Y5F2A.3 | 793.3 | 0.184 | 0.48 | 0.036 |  | Protein tyrosine phosphatase |
| F32A6.5 | 554.2 | 0.0576 | 0.48 | 0.008 | *sto-2* | Prohibitins and stomatins of the PID superfamily |
| C41G6.9 | 859.1 | 0.195 | 0.48 | 0.035 | *srh-165* | Predicted olfactory G-protein coupled receptor |
| C02E7.6 | 3.642 | 0.0894 | 0.47 | 0.007 |  | Confirmed TR:O16428 AAC24165.1 |
| B0213.14 | 513 | 0.146 | 0.47 | 0.011 | *cyp-34A8* | Cytochrome P450 CYP2 subfamily |
| F32A5.7 | 395.8 | 0.116 | 0.47 | 0.028 | *lsm-4* | Small nuclear ribonucleoprotein (snRNP) |
| F53F4.2 | 710 | 0.169 | 0.47 | 0.044 |  |  |
| F34D10.3 | 967.1 | 0.131 | 0.47 | 0.028 |  |  |
| C43D7.7 | 780.5 | 0.169 | 0.47 | 0.018 |  |  |
| C04F5.3 | 537.7 | 0.0983 | 0.47 | 0.007 | *unc-46* |  |
| F32B5.7 | 704.6 | 0.165 | 0.47 | 0.035 |  | Unnamed protein |
| M60.4 | 1.779 | 0.0952 | 0.47 | 0.004 |  |  |
| F30F8.7 | 953.9 | 0.144 | 0.47 | 0.010 |  |  |
| K09F6.7 | 1.279 | 0.0891 | 0.47 | 0.021 |  | Predicted E3 ubiquitin ligase |
| Y73B3A.17 | 783.9 | 0.091 | 0.47 | 0.009 |  | AAA ATPase containing von Willebrand factor type A (vWA) domain |
| C39H7.5 | 1.922 | 0.154 | 0.47 | 0.020 | *srsx-14* |  |
| H17B01.1a | 1.604 | 0.134 | 0.47 | 0.009 |  | Permease of the major facilitator superfamily |
| F31E3.2c | 834 | 0.219 | 0.47 | 0.028 |  |  |
| F38H4.8 | 583.2 | 0.123 | 0.46 | 0.010 | *ech-2* | Enoyl-CoA isomerase |
| F40D4.10 | 520.7 | 0.136 | 0.46 | 0.021 |  | No Significant Match |
| F12A10.4 | 596 | 0.0906 | 0.46 | 0.016 |  | M13 family peptidase |
| C10F3.5 | 4.279 | 0.127 | 0.46 | 0.022 | *pcm-1* | Protein-L-isoaspartate(D-aspartate) O-methyltransferase |
| F14B6.6 | 584.5 | 0.105 | 0.46 | 0.006 |  | Galactosyltransferases |
| C11H1.5 | 636.6 | 0.11 | 0.46 | 0.005 |  |  |
| C34D1.5 | 779.3 | 0.126 | 0.46 | 0.014 |  |  |
| F07C3.2 | 461.1 | 0.102 | 0.46 | 0.005 |  |  |
| F09A5.2 | 668.2 | 0.101 | 0.46 | 0.005 |  | Fibroblast/platelet-derived growth factor receptor and related receptor tyrosine kinases |
| K07E8.7 | 15.500 | 0.0707 | 0.46 | 0.045 |  | RNA pseudouridylate synthases |
| Y73B6BL.7 | 629.7 | 0.0781 | 0.46 | 0.001 | *csp-2* | Caspase, apoptotic cysteine protease |
| M28.6 | 896.7 | 0.105 | 0.46 | 0.001 |  | Predicted esterase |
| D2085.7 | 1.229 | 0.147 | 0.46 | 0.020 |  | Uncharacterized conserved protein |
| C01F1.5 | 626.4 | 0.145 | 0.46 | 0.019 |  |  |
| C03F11.2 | 787.3 | 0.0992 | 0.46 | 0.007 |  | Uncharacterized conserved protein |
| Y39H10B.1 | 677.7 | 0.123 | 0.46 | 0.041 | *str-113* | 7-transmembrane olfactory receptor |

| ZK1098.2a | 671.4 | 0.0763 | 0.45 | 0.003 |  | Unnamed protein |
| --- | --- | --- | --- | --- | --- | --- |
| T20G5.10 | 616.7 | 0.167 | 0.45 | 0.034 |  | General control of amino-acid synthesis 5-like 1 |
| C44B7.11 | 857.5 | 0.0594 | 0.45 | 0.015 |  | Aminopeptidases of the M20 family |
| F58F6.4 | 634.7 | 0.0908 | 0.45 | 0.009 | *rfc-2* | Replication factor C, subunit RFC2 |
| T12D8.9 | 898.3 | 0.183 | 0.45 | 0.039 |  |  |
| F37B4.10 | 748 | 0.0902 | 0.45 | 0.008 |  |  |
| E02H9.7 | 2.066 | 0.0545 | 0.45 | 0.047 |  |  |
| T09F5.9 | 1.321 | 0.135 | 0.45 | 0.012 | *clec-47* | C-type lectin |
| T12D8.7 | 845.5 | 0.152 | 0.45 | 0.020 | *taf-9* | Transcription initiation factor TFIID, subunit TAF9 (also component of histone acetyltransferase SAGA) |
| B0222.3 | 722.4 | 0.116 | 0.45 | 0.021 |  | Na+/Pi symporter |
| Y53F4B.5 | 1.267 | 0.0674 | 0.45 | 0.040 |  |  |
| F48C1.1a | 776 | 0.0931 | 0.45 | 0.012 |  | alpha-mannosidase II Partially_confirmed TR:O01574 AAB52345.1 |
| C51E3.3 | 635.4 | 0.0784 | 0.44 | 0.002 | *srsx-28* | 7-transmembrane receptor |
| C17B7.6 | 564 | 0.177 | 0.44 | 0.041 |  |  |
| F55C5.8 | 1.885 | 0.127 | 0.44 | 0.020 |  | Signal recognition particle, subunit Srp68 |
| T24H10.4 | 811.4 | 0.193 | 0.44 | 0.041 |  | Uncharacterized conserved protein |
| C07G3.3 | 544.5 | 0.045 | 0.44 | 0.001 | *str-227* | 7-transmembrane olfactory receptor |
| Y50D4B.5 | 799.3 | 0.0591 | 0.44 | 0.016 |  | C-type lectin |
| T04F8.1 | 698.4 | 0.222 | 0.44 | 0.036 |  | Sideroflexin |
| F09C6.7 | 846.5 | 0.104 | 0.44 | 0.026 | *srd-68* | Chemoreceptor/7TM receptor |
| C05D11.4 | 764 | 0.162 | 0.44 | 0.021 | *let-756* | Fibroblast growth factor |
| C25E10.9a | 1.102 | 0.147 | 0.44 | 0.013 | *isl-2* | Uncharacterized protein |
| F55G11.5 | 1.752 | 0.155 | 0.44 | 0.014 | *dod-22* | Uncharacterized protein |
| F59B2.8 | 449.3 | 0.183 | 0.43 | 0.029 |  | Uncharacterized protein |
| Y48G9A.9 | 951.9 | 0.17 | 0.43 | 0.048 |  | Protein tyrosine phosphatase |
| C49G7.5 | 585.7 | 0.13 | 0.43 | 0.015 |  | Unnamed protein |
| F21F8.11 | 5.333 | 0.0864 | 0.43 | 0.001 |  | Permease of the major facilitator superfamily |
| Y57G11B.3 | 763.6 | 0.13 | 0.43 | 0.013 |  | Predicted globin |
| R10H10.3 | 886.4 | 0.169 | 0.43 | 0.042 |  | C-type lectin |
| F08F1.5 | 652.3 | 0.0481 | 0.43 | 0.001 | *ced-8* | Uncharacterized conserved protein |
| C12D12.3 | 2.183 | 0.207 | 0.42 | 0.047 |  |  |
| C45H4.2 | 2.318 | 0.139 | 0.42 | 0.027 | *cyp-33C1* | Cytochrome P450 CYP2 subfamily |
| F55A4.5 | 415.7 | 0.0988 | 0.42 | 0.022 |  | Staufen and related double-stranded-RNA-binding proteins |
| F17E5.2 | 10.137 | 0.149 | 0.42 | 0.014 |  | Predicted mitochondrial carrier protein |
| F02A9.3 | 7.066 | 0.142 | 0.42 | 0.019 | *far-2* | Unnamed protein |
| C50F4.9 | 795.3 | 0.119 | 0.42 | 0.035 |  |  |
| E04A4.7 | 2.377 | 0.157 | 0.42 | 0.018 |  | Cytochrome c |
| C35C5.6 | 521.6 | 0.121 | 0.42 | 0.031 |  | Targeting complex (TRAPP) subunit |
| M88.3 | 724.8 | 0.133 | 0.42 | 0.025 |  | Uncharacterized protein |
| C45H4.11 | 1.050 | 0.195 | 0.41 | 0.046 | *srbc-20* | Unnamed protein |
| C01G10.3 | 524.7 | 0.118 | 0.41 | 0.017 | *srx-78* | G protein coupled protein |
| F29A7.1 | 920.4 | 0.0736 | 0.41 | 0.001 |  | Predicted transposase |
| F20H11.5 | 520.3 | 0.166 | 0.41 | 0.014 |  | D-aspartate oxidase |
| F55C12.4 | 693.8 | 0.15 | 0.41 | 0.032 |  |  |
| B0285.10 | 1.096 | 0.112 | 0.41 | 0.007 | *ckb-3* | Choline kinase |
| F57F5.1 | 1.143 | 0.141 | 0.41 | 0.012 |  | Cysteine proteinase Cathepsin L |
| C49F5.4 | 499.1 | 0.151 | 0.41 | 0.018 |  | Nuclear hormone receptor |
| R05D3.8 | 631.8 | 0.164 | 0.41 | 0.022 |  |  |
| F44C8.6 | 8.200 | 0.113 | 0.40 | 0.004 | *nhr-56* | Hormone receptors |
| F15E11.4 | 6.866 | 0.12 | 0.40 | 0.004 |  | Predicted receptor |
| D1009.5 | 695 | 0.225 | 0.40 | 0.049 | *dylt-2* | Dynein light chain |
| F12A10.6 | 6.395 | 0.0933 | 0.40 | 0.004 |  | Unnamed protein |
| Y66H1A.2 | 890 | 0.0749 | 0.40 | 0.039 |  | Dolichol-phosphate mannosyltransferase |
| T10C6.10 | 589.2 | 0.0795 | 0.39 | 0.000 |  | Uncharacterized protein |
| Y105E8A.16 | 1.529 | 0.16 | 0.39 | 0.017 | *rps-20* | 40S ribosomal protein S20 |
| ZK930.2 | 790.2 | 0.191 | 0.39 | 0.030 |  | Uncharacterized conserved protein (tumor-associated antigen HCA127 in humans) |
| Y69H2.13 | 2.475 | 0.082 | 0.39 | 0.017 |  | Non voltage-gated ion channels (DEG/ENaC family) |
| C25E10.11 | 556 | 0.144 | 0.39 | 0.006 |  |  |

| C14A6.5 | 1.042 | 0.138 | 0.39 | 0.018 |  | Unnamed protein |
| --- | --- | --- | --- | --- | --- | --- |
| ZC196.5 | 1.007 | 0.0682 | 0.39 | 0.016 |  | Unnamed protein |
| T10B10.9 | 20.283 | 0.221 | 0.39 | 0.039 |  |  |
| C24A3.4 | 832.8 | 0.116 | 0.39 | 0.018 |  | Predicted L-carnitine dehydratase/alpha-methylacyl-CoA racemase |
| B0035.2 | 639.7 | 0.191 | 0.39 | 0.032 | *dnj-2* | Molecular chaperone (DnaJ superfamily) |
| C49H3.9 | 2.088 | 0.151 | 0.39 | 0.013 |  | Uncharacterized conserved protein |
| C06C6.5a | 1.062 | 0.226 | 0.38 | 0.049 | *nhr-50* | Hormone receptors |
| M176.7 | 257.9 | 0.17 | 0.38 | 0.043 | *kin-16* | Fibroblast/platelet-derived growth factor receptor and related receptor tyrosine kinases |
| ZC581.9 | 647.5 | 0.0971 | 0.38 | 0.048 |  | Protein kinase |
| T11F9.11 | 522.8 | 0.09 | 0.38 | 0.010 | *dhs-19* | Hydroxysteroid 17-beta dehydrogenase 11 |
| K02D3.2 | 752.3 | 0.115 | 0.38 | 0.031 |  | MLN, STAR and related lipid-binding proteins |
| C33E10.10 | 887.5 | 0.173 | 0.38 | 0.011 |  | Predicted dehydrogenase |
| E03D2.1 | 890.8 | 0.18 | 0.38 | 0.041 | *nlp-13* | Unnamed protein |
| F09G2.5 | 1.026 | 0.11 | 0.38 | 0.008 |  | Ligand-gated ion channel |
| K06B9.4 | 612 | 0.193 | 0.37 | 0.031 |  |  |
| T11F9.6 | 867 | 0.0934 | 0.37 | 0.010 | *nas-22* | Meprin A metalloprotease |
| C14B4.2 | 985.6 | 0.181 | 0.37 | 0.026 |  |  |
| R08C7.10c | 789.8 | 0.108 | 0.37 | 0.010 |  | Sister chromatid cohesion protein |
| F25B5.7c | 702.6 | 0.138 | 0.37 | 0.007 |  | Confirmed TR:Q95QJ0 AAL02496.1 |
| F57C7.3a | 782.4 | 0.0636 | 0.37 | 0.025 | *sdn-1* | Heparan sulfate proteoglycan Syndecan |
| M28.9 | 794.5 | 0.226 | 0.36 | 0.046 |  | Unnamed protein |
| C07D8.2 | 475.3 | 0.115 | 0.36 | 0.041 |  | Transposon-encoded proteins with TYA, reverse transcriptase, integrase domains in various combinations |
| F57B10.12 | 820 | 0.135 | 0.36 | 0.008 | *mei-2* | Unnamed protein |
| F08D12.2 | 1.218 | 0.0992 | 0.36 | 0.004 |  | Unnamed protein |
| R07E5.14 | 762.9 | 0.115 | 0.36 | 0.005 | *rnp-4* | RNA-binding protein RBM8/Tsunagi (RRM superfamily) |
| C47B2.8 | 1.011 | 0.131 | 0.36 | 0.018 | *prx-11* | Peroxisomal biogenesis protein (peroxin) |
| C05B5.3 | 537 | 0.182 | 0.35 | 0.039 | *pqn-8* |  |
| Y59A8B.2 | 549.2 | 0.117 | 0.35 | 0.034 |  | Ubiquitin C-terminal hydrolase |
| C33G3.1b | 750.8 | 0.143 | 0.34 | 0.020 | *dyc-1* | Nitric oxide synthase-binding protein, contains PTB domain |
| F29D11.2 | 367 | 0.131 | 0.34 | 0.008 |  |  |
| C04E12.10 | 681.2 | 0.0909 | 0.34 | 0.017 |  | Unnamed protein |
| C25A1.7b | 649.7 | 0.159 | 0.34 | 0.033 |  |  |
| K11B4.2 | 752.7 | 0.173 | 0.34 | 0.022 |  | Uncharacterized conserved protein |
| C15A7.2 | 594.2 | 0.227 | 0.34 | 0.031 |  | Predicted membrane protein |
| F12B6.2c | 676.2 | 0.155 | 0.34 | 0.008 |  |  |
| C03G6.9 | 587.4 | 0.177 | 0.33 | 0.019 | *srh-244* | Predicted olfactory G-protein coupled receptor |
| F30A10.7 | 661.1 | 0.232 | 0.33 | 0.044 |  | Unnamed protein |
| F26A3.8 | 631.1 | 0.249 | 0.33 | 0.047 | *rrf-1* | RNA-directed RNA polymerase QDE-1 required for posttranscriptional gene silencing and RNA interference |
| C32B5.10 | 712.3 | 0.115 | 0.33 | 0.039 |  | F-box domain |
| C44E4.1a | 676.6 | 0.215 | 0.33 | 0.037 |  |  |
| Y54E2A.6 | 682.7 | 0.162 | 0.32 | 0.011 |  | C-type lectin |
| Y37H2A.7 | 757 | 0.137 | 0.32 | 0.004 |  | Uncharacterized protein |
| T14B4.6 | 339.9 | 0.173 | 0.32 | 0.010 | *dpy-2* | Collagens (type IV and type XIII), and related proteins |
| F57A8.7 | 532.1 | 0.142 | 0.32 | 0.007 |  | Predicted transporter/transmembrane protein |
| ZK1010.5 | 754.7 | 0.107 | 0.30 | 0.002 |  | Unnamed protein |
| ZK1251.9 | 969.9 | 0.182 | 0.30 | 0.037 |  | HIV-1 Vpr-binding protein |
| T13A10.2 | 617.6 | 0.245 | 0.30 | 0.026 |  | Predicted E3 ubiquitin ligase |
| C46A5.6 | 712.8 | 0.107 | 0.30 | 0.016 |  |  |
| B0414.2 | 693.5 | 0.118 | 0.30 | 0.004 | *rnt-1* | Runt and related transcription factors |
| T21D12.3 | 371.3 | 0.0979 | 0.29 | 0.048 |  | Polyglutamine tract-binding protein PQBP-1 |
| B0379.1 | 750 | 0.148 | 0.29 | 0.007 |  |  |
| T03G11.4 | 451.5 | 0.201 | 0.29 | 0.036 |  | 1, 2-alpha-mannosidase |
| Y46G5A.8 | 818.2 | 0.143 | 0.28 | 0.009 |  | Unnamed protein |
| C03G6.5 | 1.509 | 0.237 | 0.28 | 0.038 |  | Predicted secreted cysteine rich protein found only in C.elegans |
| C47G2.1 | 779.3 | 0.12 | 0.28 | 0.022 | *cut-1* | Cuticulin precursor |
| F45D11.12 | 580.4 | 0.225 | 0.27 | 0.034 |  | Predicted transposase |
| C38C3.6 | 995.1 | 0.175 | 0.27 | 0.018 |  |  |
| F31F7.3 | 897.7 | 0.255 | 0.26 | 0.043 |  |  |

| C55B7.12 | 423.4 | 0.105 | 0.25 | 0.008 |  | No Significant Match |
| --- | --- | --- | --- | --- | --- | --- |
| VW02B12L.3 | 553.9 | 0.0866 | 0.25 | 0.017 | *ebp-2* | Microtubule-binding protein involved in cell cycle control |
| F46G11.1 | 1.881 | 0.149 | 0.24 | 0.010 |  | Predicted mitochondrial DNA helicase twinkle |
| R08E5.4 | 556.4 | 0.201 | 0.22 | 0.030 |  | Unnamed protein |
| R08E5.1 | 523.6 | 0.356 | 0.22 | 0.026 |  | SAM-dependent methyltransferases |
| F02E11.4 | 461 | 0.184 | 0.18 | 0.047 |  | Unnamed protein |
| C14C11.5 | 576.8 | 0.241 | 0.17 | 0.022 | *srx-117* | 7-transmembrane receptor |
| W07G1.4 | 768.4 | 0.28 | 0.13 | 0.029 |  | No Significant Match |
