# Supplementary material for: Gene expression profiling to characterize sediment toxicity – a pilot study using Caenorhabditis elegans whole genome microarrays
Source: BMC Genomics. 2009 Apr 14;10:160. doi: 10.1186/1471-2164-10-160 (PMC2674462; doi:10.1186/1471-2164-10-160)
Supplement: Additional file 3 — List of all differentially regulated GO categories. Biological GO processes overrepresented in sediment exposed nematodes using the Danube sediment sample as reference. [file 1471-2164-10-160-S3.doc]

### Additional file 3 – List of all differentially regulated GO categories

Biological GO processes overrepresented in sediment exposed nematodes using the Danube sediment sample as reference.

| **Category** | **Listed genes** | **Number of genes in the samples** | | **Statistical significance (p-value according to Holm-Bonferroni)** | |
| --- | --- | --- | --- | --- | --- |
| **BIOLOGICAL PROCESS** |  | **Rhine** | **Elbe** | **Rhine** | **Elbe** |
| GO:0008150; biological process | 8670 | 253 | 306 | 0.0E+00 | 0.0E+00 |
| GO:0009987; cellular process | 4979 | 127 | 163 | 1.9E-18 | 7.6E-28 |
| GO:0008152; metabolic process | 4233 | 114 | 141 | 1.9E-17 | 3.4E-23 |
| GO:0032501; multicellular organismal process | 3613 | 94 | 137 | 3.2E-12 | 5.1E-28 |
| GO:0032502; developmental process | 3483 | 94 | 136 | 3.1E-13 | 4.5E-29 |
| GO:0044238; primary metabolic process | 3385 | 89 | 111 | 1.4E-11 | 6.2E-16 |
| GO:0007275; multicellular organismal development | 3350 | 90 | 133 | 2.5E-12 | 6.0E-29 |
| GO:0044237; metabolic process | 3322 | 88 | 111 | 1.4E-11 | 1.5E-16 |
| GO:0043170; macromolecule metabolic process | 2767 | 82 | 90 | 3.0E-13 | 8.9E-12 |
| GO:0065007; biological regulation | 2738 | 69 | 90 | 2.7E-07 | 4.7E-12 |
| GO:0009790; embryonic development | 2611 | 71 | 104 | 4.0E-09 | 4.9E-21 |
| GO:0050789; regulation of biological process | 2587 | 66 | 87 | 4.5E-07 | 4.3E-12 |
| GO:0009792; embryonic development ending in birth | 2547 | 69 | 101 | 1.1E-08 | 4.1E-20 |
| GO:0040007; growth | 2040 | 50 | 72 | 3.2E-04 | 1.9E-10 |
| GO:0043283; biopolymer metabolic process | 1971 | 60 | 62 | 3.1E-09 | 1.4E-06 |
| GO:0000003; reproduction | 1842 | 47 | 65 | 2.4E-04 | 3.8E-09 |
| GO:0009791; post-embryonic development | 1674 | 38 | 61 | 5.1E-02 | 5.3E-09 |
| GO:0048518; positive regulation of biological process | 1647 | 42 | 59 | 1.0E-03 | 2.5E-08 |
| GO:0044260; cellular macromolecule metabolic process | 1592 | 49 | 56 | 3.1E-07 | 1.7E-07 |
| GO:0019538; protein metabolic process | 1585 | 47 | 53 | 2.5E-06 | 3.4E-06 |
| GO:0051179; localization | 1578 | 45 | 61 | 1.9E-05 | 4.3E-10 |
| GO:0040008; regulation of growth | 1574 | 42 | 57 | 3.6E-04 | 3.8E-08 |
| GO:0002164; larval development | 1555 | 33 | 56 | 5.2E-01 | 7.2E-08 |
| GO:0002119; nematode larval development | 1553 | 33 | 56 | 5.1E-01 | 6.9E-08 |
| GO:0044267; cellular protein metabolic process | 1531 | 47 | 52 | 8.3E-07 | 2.9E-06 |
| GO:0045927; positive regulation of growth | 1527 | 41 | 57 | 4.3E-04 | 1.2E-08 |
| GO:0050896; response to stimulus | 1478 | 37 | 56 | 8.0E-03 | 1.0E-08 |
| GO:0051234; establishment of localization | 1473 | 43 | 58 | 2.1E-05 | 8.5E-10 |
| GO:0006810; transport | 1385 | 43 | 53 | 3.5E-06 | 2.8E-08 |
| GO:0006139; nucleobase, nucleoside, nucleotide and | 1331 | 32 | 42 | 7.2E-02 | 7.7E-04 |
| GO:0040009; regulation of growth rate | 1305 | 31 | 52 | 1.2E-01 | 1.0E-08 |
| GO:0040010; positive regulation of growth rate | 1304 | 31 | 52 | 1.1E-01 | 9.8E-09 |
| GO:0007610; behavior | 1204 | 31 | 44 | 2.7E-02 | 6.4E-06 |
| GO:0010467; gene expression | 1134 | 31 | 41 | 8.0E-03 | 3.0E-05 |
| GO:0048856; anatomical structure development | 1113 | 23 | 47 | 1.0E+00 | 1.6E-08 |
| GO:0050794; regulation of cellular process | 1041 | 28 | 35 | 3.1E-02 | 2.0E-03 |
| GO:0007626; locomotory behavior | 987 | 25 | 35 | 2.0E-01 | 5.0E-04 |
| GO:0022414; reproductive process | 834 | 23 | 35 | 1.1E-01 | 8.7E-06 |
| GO:0016043; cellular component organization and bi | 825 | 18 | 32 | 1.0E+00 | 2.3E-04 |
| GO:0043412; biopolymer modification | 823 | 29 | 28 | 1.1E-04 | 1.5E-02 |
| GO:0019222; regulation of metabolic process | 763 | 21 | 26 | 2.1E-01 | 2.8E-02 |
| GO:0006464; protein modification process | 761 | 25 | 25 | 3.0E-03 | 7.0E-02 |
| GO:0031323; regulation of cellular metabolic process | 756 | 21 | 26 | 1.9E-01 | 2.4E-02 |
| GO:0019219; regulation of nucleobase, nucleoside, | 726 | 20 | 25 | 2.9E-01 | 3.4E-02 |
| GO:0019953; sexual reproduction | 723 | 16 | 27 | 1.0E+00 | 4.0E-03 |
| GO:0009653; anatomical structure morphogenesis | 709 | 15 | 31 | 1.0E+00 | 2.5E-05 |
| GO:0048731; system development | 706 | 18 | 32 | 1.0E+00 | 6.4E-06 |
| GO:0007276; gamete generation | 678 | 16 | 25 | 1.0E+00 | 1.1E-02 |
| GO:0043687; post-translational protein modification | 669 | 21 | 24 | 3.5E-02 | 2.5E-02 |
| GO:0032774; RNA biosynthetic process | 668 | 21 | 19 | 3.4E-02 | 1.0E+00 |
| GO:0006351; transcription, DNA-dependent | 665 | 21 | 19 | 3.2E-02 | 1.0E+00 |
| GO:0048513; organ development | 661 | 17 | 31 | 1.0E+00 | 5.0E-06 |

| GO:0009058; biosynthetic process | 647 | 17 | 24 | 1.0E+00 | 1.5E-02 |
| --- | --- | --- | --- | --- | --- |
| GO:0006811; ion transport | 640 | 18 | 29 | 4.5E-01 | 3.3E-05 |
| GO:0003006; reproductive developmental process | 568 | 18 | 26 | 1.1E-01 | 1.4E-04 |
| GO:0007548; sex differentiation | 545 | 16 | 26 | 5.7E-01 | 6.3E-05 |
| GO:0048806; genitalia development | 494 | 14 | 24 | 1.0E+00 | 1.4E-04 |
| GO:0040035; hermaphrodite genitalia development | 488 | 14 | 24 | 1.0E+00 | 1.2E-04 |
| GO:0035264; multicellular organism growth | 476 | 17 | 16 | 4.0E-02 | 9.6E-01 |
| GO:0040014; regulation of multicellular organism growth | 476 | 17 | 16 | 4.0E-02 | 9.5E-01 |
| GO:0010171; body morphogenesis | 361 | 7 | 16 | 1.0E+00 | 4.8E-02 |
| GO:0051641; cellular localization | 318 | 5 | 15 | 1.0E+00 | 4.0E-02 |
| GO:0051649; establishment of cellular localization | 312 | 5 | 15 | 1.0E+00 | 3.3E-02 |
| GO:0032504; multicellular organism reproduction | 291 | 9 | 15 | 1.0E+00 | 1.5E-02 |
| GO:0048609; reproductive process in a multicellular org. | 291 | 9 | 15 | 1.0E+00 | 1.5E-02 |
| GO:0018991; oviposition | 282 | 9 | 14 | 1.0E+00 | 4.1E-02 |
| GO:0033057; reproductive behavior in a multicellular org. | 282 | 9 | 14 | 1.0E+00 | 4.1E-02 |
| GO:0048519; negative regulation of biological process | 220 | 7 | 13 | 1.0E+00 | 1.3E-02 |
| GO:0007568; aging | 195 | 4 | 13 | 1.0E+00 | 3.0E-03 |
| GO:0008340; determination of adult life span | 193 | 4 | 13 | 1.0E+00 | 3.0E-03 |
| GO:0010259; multicellular organismal aging | 193 | 4 | 13 | 1.0E+00 | 3.0E-03 |
| GO:0050793; regulation of developmental process | 189 | 5 | 12 | 1.0E+00 | 1.3E-02 |
| GO:0007017; microtubule-based process | 149 | 1 | 10 | 1.0E+00 | 4.0E-02 |
| GO:0044262; cellular carbohydrate metabolic process | 136 | 4 | 10 | 1.0E+00 | 1.8E-02 |
| GO:0048523; negative regulation of cellular process | 103 | 4 | 10 | 1.0E+00 | 2.0E-03 |
| GO:0051093; negative regulation of developmental process | 67 | 2 | 8 | 1.0E+00 | 3.0E-03 |
|  | | | | | |
| **MOLECULAR FUNCTION** |  | **Rhine** | **Elbe** | **Rhine** | **Elbe** |
| GO:0003674; molecular function | 9083 | 294 | 361 | 0.0E+00 | 0.0E+00 |
| GO:0005488; binding | 5781 | 182 | 234 | 2.3E-34 | 1.1E-49 |
| GO:0003824; catalytic activity | 3600 | 103 | 126 | 6.0E-12 | 1.0E-14 |
| GO:0005515; protein binding | 2331 | 60 | 94 | 3.7E-04 | 1.0E-13 |
| GO:0043167; ion binding | 1600 | 58 | 52 | 2.3E-09 | 1.0E-03 |
| GO:0043169; cation binding | 1541 | 57 | 51 | 1.6E-09 | 9.8E-04 |
| GO:0046872; metal ion binding | 1531 | 53 | 48 | 1.2E-07 | 8.0E-03 |
| GO:0003676; nucleic acid binding | 1519 | 44 | 52 | 7.0E-04 | 2.9E-04 |
| GO:0016787; hydrolase activity | 1489 | 40 | 51 | 1.2E-02 | 3.6E-04 |
| GO:0000166; nucleotide binding | 1300 | 30 | 47 | 1.0E+00 | 2.3E-04 |
| GO:0046914; transition metal ion binding | 1258 | 43 | 37 | 1.0E-05 | 2.5E-01 |
| GO:0004871; signal transducer activity | 1157 | 36 | 32 | 1.0E-03 | 1.0E+00 |
| GO:0060089; molecular transducer activity | 1157 | 36 | 32 | 1.0E-03 | 1.0E+00 |
| GO:0017076; purine nucleotide binding | 1154 | 27 | 40 | 1.0E+00 | 5.0E-03 |
| GO:0016740; transferase activity | 1131 | 38 | 38 | 1.1E-04 | 1.5E-02 |
| GO:0008270; zinc ion binding | 1083 | 37 | 30 | 1.1E-04 | 1.0E+00 |
| GO:0032553; ribonucleotide binding | 1081 | 27 | 38 | 7.1E-01 | 6.0E-03 |
| GO:0032555; purine ribonucleotide binding | 1081 | 27 | 38 | 7.1E-01 | 6.0E-03 |
| GO:0004872; receptor activity | 1076 | 35 | 32 | 7.2E-04 | 4.8E-01 |
| GO:0030554; adenyl nucleotide binding | 993 | 24 | 36 | 1.0E+00 | 5.0E-03 |
| GO:0032559; adenyl ribonucleotide binding | 931 | 24 | 34 | 8.3E-01 | 8.0E-03 |
| GO:0005524; ATP binding | 930 | 24 | 33 | 8.2E-01 | 1.7E-02 |
| GO:0005215; transporter activity | 845 | 27 | 28 | 1.6E-02 | 2.0E-01 |
| GO:0016491; oxidoreductase activity | 588 | 19 | 25 | 1.9E-01 | 9.0E-03 |
| GO:0022891; substrate-specific transmembrane transport | 526 | 16 | 22 | 9.2E-01 | 3.4E-02 |
| GO:0005198; structural molecule activity | 500 | 20 | 30 | 8.0E-03 | 7.7E-07 |
| GO:0030246; carbohydrate binding | 326 | 12 | 17 | 6.9E-01 | 1.6E-02 |
| GO:0003707; steroid hormone receptor activity | 275 | 13 | 6 | 4.4E-02 | 1.0E+00 |
|  | | | | | |
| **CELLULAR COMPONENT** |  | **Rhine** | **Elbe** | **Rhine** | **Elbe** |
| GO:0005575; cellular component | 5094 | 146 | 164 | 0.0E+00 | 0.0E+00 |
| GO:0005623; cell | 4878 | 139 | 158 | 3.5E-79 | 1.3E-92 |

| GO:0044464; cell part | 4860 | 139 | 158 | 2.1E-79 | 7.4E-93 |
| --- | --- | --- | --- | --- | --- |
| GO:0005622; intracellular | 2858 | 76 | 94 | 3.3E-27 | 1.6E-38 |
| GO:0044424; intracellular part | 2329 | 62 | 81 | 7.5E-21 | 2.8E-33 |
| GO:0016020; membrane | 2253 | 67 | 71 | 7.7E-26 | 1.8E-25 |
| GO:0043226; organelle | 1788 | 53 | 62 | 3.2E-19 | 1.0E-23 |
| GO:0043229; intracellular organelle | 1780 | 53 | 62 | 2.6E-19 | 8.0E-24 |
| GO:0044425; membrane part | 1490 | 36 | 43 | 1.9E-09 | 1.9E-12 |
| GO:0043227; membrane-bound organelle | 1446 | 44 | 46 | 1.0E-15 | 4.3E-15 |
| GO:0043231; intracellular membrane-bound organelle | 1440 | 44 | 46 | 8.6E-16 | 3.7E-15 |
| GO:0031224; intrinsic to membrane | 1307 | 33 | 40 | 5.1E-09 | 3.0E-12 |
| GO:0016021; integral to membrane | 1299 | 33 | 39 | 4.4E-09 | 1.2E-11 |
| GO:0005634; nucleus | 1153 | 35 | 36 | 5.7E-12 | 4.3E-11 |
| GO:0005737; cytoplasm | 1092 | 26 | 39 | 2.9E-06 | 4.5E-14 |
| GO:0032991; macromolecular complex | 705 | 18 | 26 | 2.1E-04 | 3.8E-09 |
| GO:0044444; cytoplasmic part | 625 | 16 | 23 | 7.9E-04 | 6.2E-08 |
| GO:0044422; organelle part | 572 | 19 | 22 | 1.8E-06 | 6.7E-08 |
| GO:0044446; intracellular organelle part | 565 | 19 | 21 | 1.5E-06 | 3.2E-07 |
| GO:0043234; protein complex | 531 | 12 | 15 | 3.8E-02 | 2.0E-03 |
| GO:0043228; non-membrane-bound organelle | 530 | 17 | 23 | 1.8E-05 | 2.4E-09 |
| GO:0043232; intracellular non-membrane-bound organelle | 530 | 17 | 23 | 1.8E-05 | 2.4E-09 |
| GO:0005694; chromosome | 193 | 10 | 7 | 1.2E-04 | 9.7E-02 |
| GO:0030529; ribonucleoprotein complex | 185 | 8 | 11 | 6.0E-03 | 2.7E-05 |
| GO:0044428; nuclear part | 155 | 3 | 8 | 1.0E+00 | 4.0E-03 |
| GO:0044427; chromosomal part | 150 | 8 | 4 | 1.0E-03 | 1.0E+00 |
| GO:0005840; ribosome | 141 | 6 | 9 | 5.5E-02 | 2.0E-04 |
| GO:0000785; chromatin | 122 | 6 | 3 | 2.6E-02 | 1.0E+00 |
| GO:0031974; membrane-enclosed lumen | 100 | 2 | 7 | 1.0E+00 | 2.0E-03 |
| GO:0043233; organelle lumen | 99 | 2 | 7 | 1.0E+00 | 2.0E-03 |
| GO:0031975; envelope | 84 | 5 | 3 | 3.7E-02 | 1.0E+00 |
| GO:0031981; nuclear lumen | 78 | 2 | 6 | 1.0E+00 | 4.0E-03 |
| GO:0005654; nucleoplasm | 60 | 2 | 5 | 1.0E+00 | 1.3E-02 |
| GO:0005740; mitochondrial envelope | 48 | 4 | 1 | 4.1E-02 | 1.0E+00 |
| GO:0031966; mitochondrial membrane | 40 | 4 | 0 | 2.0E-02 | 1.0E+00 |
| GO:0019866; organelle inner membrane | 35 | 4 | 0 | 1.2E-02 | 1.0E+00 |
| GO:0005743; mitochondrial inner membrane | 32 | 4 | 0 | 8.0E-03 | 1.0E+00 |
| GO:0005624; membrane fraction | 18 | 0 | 3 | 1.0E+00 | 3.9E-02 |

| **p < 1.0E-03** | **p < 1.0E-02** | **p < 5.0E-02** |
| --- | --- | --- |
