# Supplementary material for: Gene expression profiling to characterize sediment toxicity – a pilot study using Caenorhabditis elegans whole genome microarrays
Source: BMC Genomics. 2009 Apr 14;10:160. doi: 10.1186/1471-2164-10-160 (PMC2674462; doi:10.1186/1471-2164-10-160)
Supplement: Additional file 4 — Molecular functions – partial GO tree. Partial GO tree presenting relevant molecular functions which were found to be overrepresented in C. elegans exposed to Elbe and/or Rhine sediments. [file 1471-2164-10-160-S4.doc]

### Additional file 4 – Molecular functions - partial GO tree

Partial GO tree presenting relevant molecular functions which were found to be overrepresented in *C. elegans* exposed to Elbe and/or Rhine sediments.


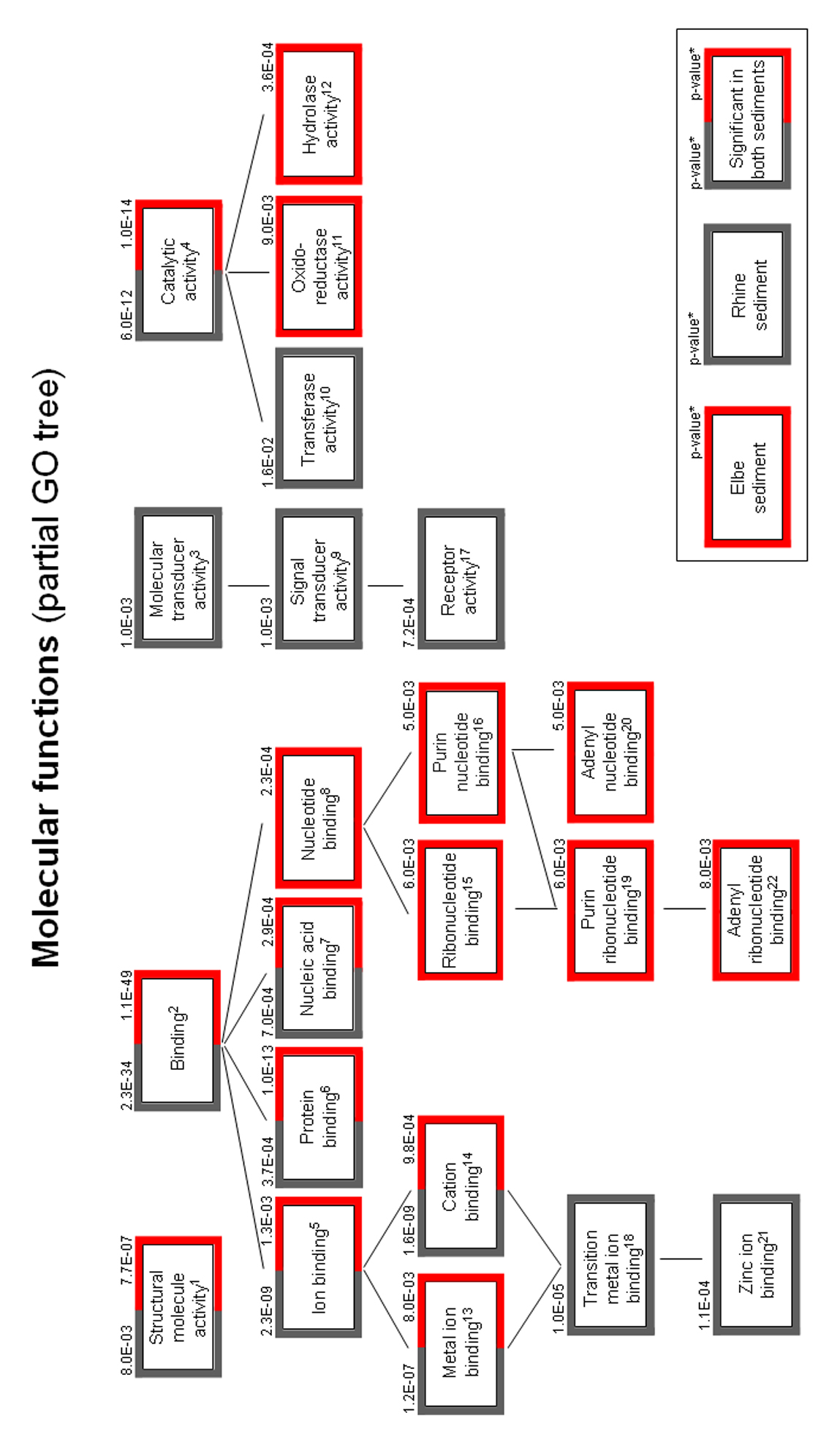


GO annotations: ^1^GO:0005198, ^2^GO:0005488, ^3^GO:0060089, ^4^GO:0003824, ^5^GO:0043167, ^6^GO:0005515, ^7^GO:0003676, ^8^GO:0000166, ^9^GO:0004871, ^10^GO:0016740, ^11^GO:0016491, ^12^GO:0016787, ^13^GO:0046872, ^14^GO:0043169, ^15^GO:0032553, ^16^GO:0017076, ^17^GO:0004872, ^18^GO:0046914, ^19^GO:0032555, ^20^GO:0030554, ^21^GO:0008270, ^22^GO:0032559
