# Supplementary material for: Gene expression profiling to characterize sediment toxicity – a pilot study using Caenorhabditis elegans whole genome microarrays
Source: BMC Genomics. 2009 Apr 14;10:160. doi: 10.1186/1471-2164-10-160 (PMC2674462; doi:10.1186/1471-2164-10-160)
Supplement: Additional file 5 — Cellular components – partial GO tree. Partial GO tree presenting relevant cellular components which were found to be overrepresented in C. elegans exposed to Elbe and/or Rhine sediments. [file 1471-2164-10-160-S5.doc]

### Additional file 5 – Cellular components - partial GO tree

Partial GO tree presenting relevant cellular components which were found to be overrepresented in *C. elegans* exposed to Elbe and/or Rhine sediments.


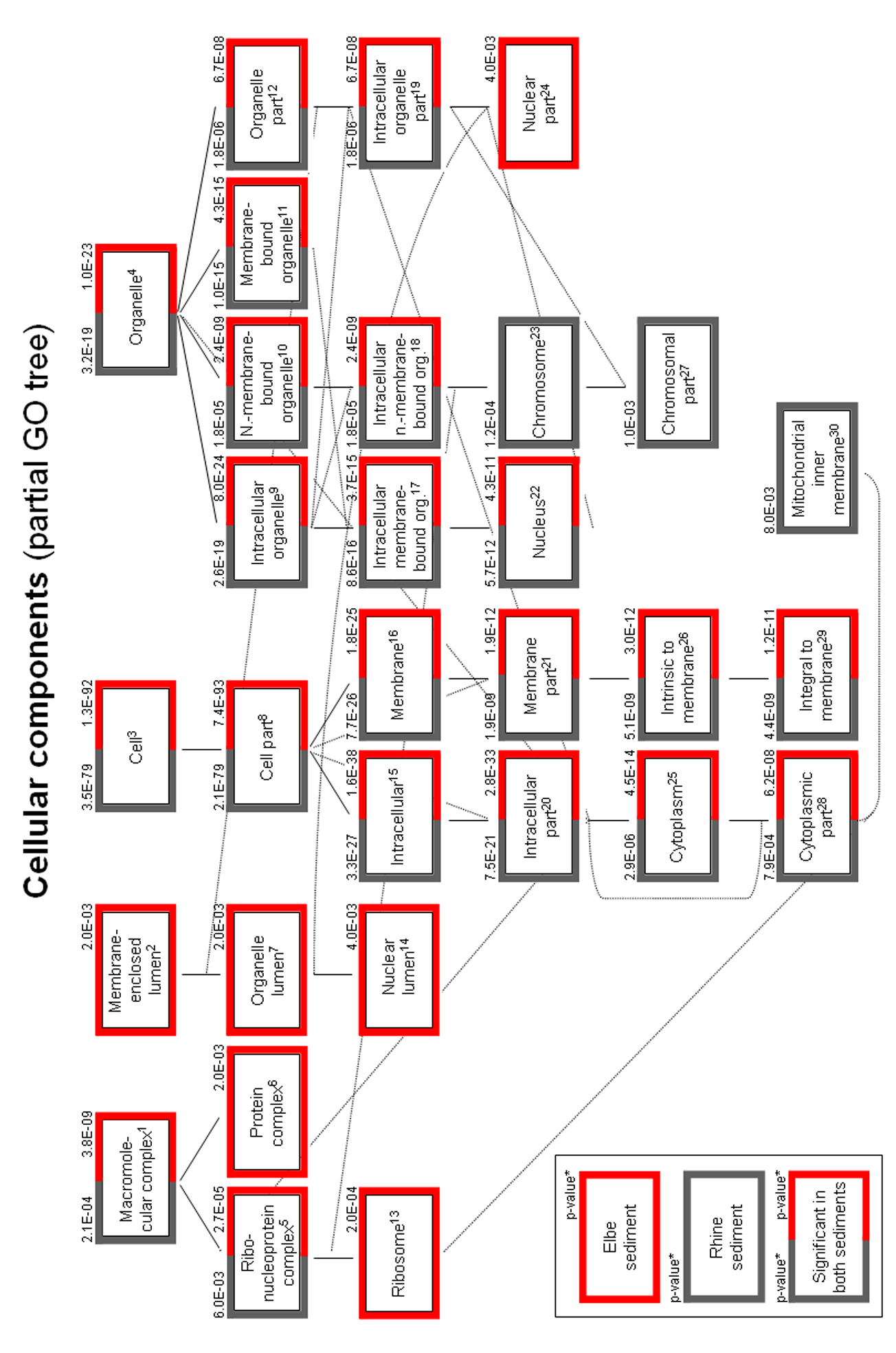


^1^GO:0032991, ^2^GO:0031974, ^3^GO:0005623, ^4^GO:0043226, ^5^GO:0030529, ^6^GO:0043234, ^7^GO:0043233, ^8^GO:0044464, ^9^GO:0043229, ^10^GO:0043228, ^11^GO:0043227, ^12^GO:0044422, ^13^GO:0005840, ^14^GO:0031981, ^15^GO:0005622, ^16^GO:0016020, ^17^GO:0043231, ^18^GO:0043232, ^19^GO:0044446, ^20^GO:0044424, ^21^GO:0044425, ^22^GO:0005634, ^23^GO:0005694, ^24^GO:0044428, ^25^GO:0005737, ^26^GO:0031224, ^27^GO:0044427, ^28^GO:0044444, ^29^GO:0016021
