# Supplementary material for: Gene expression profiling to characterize sediment toxicity – a pilot study using Caenorhabditis elegans whole genome microarrays
Source: BMC Genomics. 2009 Apr 14;10:160. doi: 10.1186/1471-2164-10-160 (PMC2674462; doi:10.1186/1471-2164-10-160)
Supplement: Additional file 6 — Overlapping differentially regulated genes of Elbe and Rhine. C. elegans transcripts that significantly changed in response to both Elbe and Rhine sediment exposure [ANOVA, p < 0.05 without multiple sample correction, fold-change to reference sediment Danube > 1.4 (up-regulated) or < 0.7 (down-regulated), for color code see below]. [file 1471-2164-10-160-S6.doc]

### Additional file 6 – Overlapping differentially regulated genes of Elbe and Rhine

*C. elegans* transcripts that significantly changed in response to both Elbe and Rhine sediment exposure [ANOVA, p<0.05 without multiple sample correction, fold-change to reference sediment Danube > 1.4 (up-regulated) or < 0.7 (down-regulated), for color code see below].

| **ID** | **Elbe sediment** | **Rhine sediment** | **CGC name** | **Description** |
| --- | --- | --- | --- | --- |
| UP-REGULATED GENES | | | | |
| F44F1.5 |  |  |  | Uncharacterized coiled-coil containing protein |
| F54D1.2 |  |  | *col-127* | Collagens (type IV and type XIII), and related proteins |
| R06A4.8 |  |  |  | Alpha amylase |
| C09F12.2 |  |  |  |  |
| C50F4.1 |  |  |  |  |
| F55G1.6 |  |  |  | Unnamed protein |
| F17H10.1 |  |  |  | Uncharacterized conserved protein |
| C54G7.3b |  |  | *lgx-1* |  |
| K09A9.3 |  |  | *ent-2* | Nucleoside transporter |
| T05H4.4 |  |  |  | NADH-cytochrome b-5 reductase |
| C16A11.4 |  |  |  | Uncharacterized protein |
| K04F1.10 |  |  |  | Predicted receptor |
| R11G11.7 |  |  | *pqn-60* | Predicted alpha-helical protein |
| T01C3.8 |  |  |  |  |
| Y116F11B.3 |  |  | *pcp-4* | Hydrolytic enzymes of the alpha/beta hydrolase fold |
| F43E2.1 |  |  |  | S-M checkpoint control protein CID1 and related nucleotidyltransferases |
| C53B7.3 |  |  |  |  |
| F48A11.5 |  |  |  |  |
| T12D8.8 |  |  |  | Hsp70-interacting protein Hip/Transient component of progesterone receptor complexes |
| B0414.3 |  |  | *hil-5* | Histone H1 |
| C53A5.3 |  |  | *hda-1* | Histone deacetylase complex, catalytic component RPD3 |
| K08C9.2 |  |  |  | Unnamed protein |
| F36H5.1 |  |  |  | Uncharacterized protein, contains BTB/POZ domain |
| F09C12.7 |  |  | *msp-74* |  |
| F41G3.5 |  |  |  | Casein kinase (serine/threonine/tyrosine protein kinase) |
| R07B5.7 |  |  |  |  |
| F13H8.10c |  |  | *bpl-1* |  |
| Y68A4A.9 |  |  |  | No Significant Match |
| F14D7.4 |  |  |  |  |
| W09H1.1 |  |  |  |  |
| K01A2.5 |  |  |  | Predicted hydrolase |
| K07A1.11 |  |  | *rba-1* | Nucleosome remodeling factor, subunit CAF1/NURF55/MSI1 |
| F29G6.3b |  |  |  | Unnamed protein |
| C25A8.4 |  |  |  | Chitinase |
| Y17G7B.8 |  |  |  | Uncharacterized protein |
| W02D7.9 |  |  |  |  |
| M03F4.7 |  |  |  | Reticulocalbin, calumenin, DNA supercoiling factor |
| K03E5.2 |  |  |  |  |
| T26E4.7 |  |  |  | Glycosyltranferase |
| T28H11.6 |  |  | *ssp-11* | Uncharacterized protein, contains major sperm protein (MSP) domain |
| Y69A2AR.21 |  |  |  | Uncharacterized conserved protein |
| T28A11.2 |  |  |  | Predicted secreted cysteine rich protein found only in C.elegans |
| C18A11.1 |  |  |  | Unnamed protein |
| F28E10.2 |  |  |  |  |
| ZK1225.4 |  |  |  | Splicing factor RNPS1, SR protein superfamily |
| F44F4.7 |  |  | *sra-12* | Sra family integral membrane protein |
| Y50E8A.9 |  |  |  | Phospholipid scramblase |
| Y51H1A.3b |  |  |  | NADH:ubiquinone oxidoreductase, NDUFB8/ASHI subunit |
| ZK858.7 |  |  |  | tRNA(1-methyladenosine) methyltransferase, subunit GCD10 |
| T22E5.1 |  |  |  |  |
| K07B1.4 |  |  |  |  |
| Y53H1C.1 |  |  | *aat-9* |  |

| Y66D12A.12 |  |  |  | Zn-finger |
| --- | --- | --- | --- | --- |
| DOWN-REGULATED GENES | | | | |
| F17H10.2 |  |  |  | Unnamed protein |
| C05E11.4 |  |  | *amt-1* | Ammonia permease |
| C26D10.6 |  |  |  | Unnamed protein |
| R17.2 |  |  |  | Glucose-repressible alcohol dehydrogenase transcriptional effector CCR4 |
| W08F4.7 |  |  |  |  |
| T05H4.5 |  |  |  | NADH-cytochrome b-5 reductase |
| C53C7.1 |  |  |  |  |
| F59F5.3 |  |  |  | Fibroblast/platelet-derived growth factor receptor |
| F45B8.3 |  |  |  |  |
| Y92C3A.1 |  |  |  | No Significant Match |
| F25H5.7 |  |  |  | Protein tyrosine phosphatase |
| T26A8.3 |  |  |  | Unnamed protein |
| C36A4.6 |  |  | *cyp-25A4* | Cytochrome P450 CYP3/CYP5/CYP6/CYP9 subfamilies |
| F01G4.5 |  |  |  | N-acetylglucosaminyltransferase complex, subunit PIG-Q/GPI1 |
| T14B4.6 |  |  | *dpy-2* | Collagens (type IV and type XIII), and related proteins |
| F17E5.1a |  |  | *lin-2* | Calcium/calmodulin-dependent serine protein kinase/membrane-associated guanylate kinase |
| C03A7.12 |  |  |  | UDP-glucuronosyl and UDP-glucosyl transferase |
| F10F2.9 |  |  | *pqn-29* |  |
| T04F8.1 |  |  |  | Sideroflexin |
| K06B9.4 |  |  |  |  |
| F20H11.5 |  |  |  | D-aspartate oxidase |
| F01D4.2 |  |  | *ugt-44* | UDP-glucuronosyl and UDP-glucosyl transferase |
| M28.6 |  |  |  | Predicted esterase |
| ZC84.5 |  |  |  | No Significant Match |
| T20B12.8 |  |  | *hmg-4* | Nucleosome-binding factor SPN, POB3 subunit |
| Y40D12A.3 |  |  | *srh-40* | Predicted olfactory G-protein coupled receptor |
| Y37F4.1 |  |  |  |  |
| B0523.1 |  |  | *kin-31* | Protein tyrosine kinase |
| F35H10.5 |  |  |  |  |
| C33E10.10 |  |  |  | Predicted dehydrogenase |
| F55G11.5 |  |  | *dod-22* | Uncharacterized protein |
| C03F11.2 |  |  |  | Uncharacterized conserved protein |
| Y43D4A.6 |  |  |  | Checkpoint kinase and related serine/threonine protein kinases |
| F12E12.4 |  |  |  | Uncharacterized protein, contains BTB/POZ domain |
| Y54E2A.6 |  |  |  | C-type lectin |
| C50A2.2 |  |  |  | Unnamed protein |
| T09F5.9 |  |  | *clec-47* | C-type lectin |
| C15A7.2 |  |  |  | Predicted membrane protein |
| T10C6.10 |  |  |  | Uncharacterized protein |
| ZK593.3 |  |  |  |  |
| C33F10.12 |  |  |  | Mitochondrial phosphate carrier protein |
| ZK1010.5 |  |  |  | Unnamed protein |
| B0213.14 |  |  | *cyp-34A8* | Cytochrome P450 CYP2 subfamily |
| C49H3.9 |  |  |  | Uncharacterized conserved protein |
| F34D10.3 |  |  |  |  |
| Y37H2A.7 |  |  |  | Uncharacterized protein |
| F48C1.1a |  |  |  | alpha-mannosidase II Partially_confirmed TR:O01574 AAB52345.1 |
| C25E10.9a |  |  | *isl-2* | Uncharacterized protein |
| C50F4.9 |  |  |  |  |
| R08C7.10c |  |  |  | Sister chromatid cohesion protein |
| F30F8.7 |  |  |  |  |
| B0414.2 |  |  | *rnt-1* | Runt and related transcription factors |
| R08E5.1 |  |  |  | SAM-dependent methyltransferases |
| C03G6.5 |  |  |  | Predicted secreted cysteine rich protein found only in *C. elegans* |
| C14C11.5 |  |  | *srx-117* | 7-transmembrane receptor |

| **<0.2**  **0.2** | **<0.3** | **<0.5** | **<0.7** | **<...>** | **>1.4** | **>2.0** | **>3.0** | **>4.0** |
| --- | --- | --- | --- | --- | --- | --- | --- | --- |
