# Supplementary material for: Gene expression profiling to characterize sediment toxicity – a pilot study using Caenorhabditis elegans whole genome microarrays
Source: BMC Genomics. 2009 Apr 14;10:160. doi: 10.1186/1471-2164-10-160 (PMC2674462; doi:10.1186/1471-2164-10-160)
Supplement: Additional file 7 — Description of 58 differentially expressed genes involved in several reproduction associated, aging regulating and/or developmental processes. Identification, direction of change and description of all 58 genes which belong to one or more of the selected GO categories. [file 1471-2164-10-160-S7.doc]

### Additional file 7 – Description of 58 differentially expressed genes involved in several aging regulating and/or developmental processes

Identification, direction of change and description of all 58 genes which belong to one or more of the selected GO categories.

|  | **ID** | **Elbe sediment** | **Rhine sediment** | **CGC name** | | **Description** | | | | **Reproduction associated^1^** | | **Organ development^2^** | **Hermaphrodite ge-nitalia development^3^** | | **Negative regulation of cellular process^4^** | **Negative regulation of develop. Process^5^** | | **Determination of adult life span^6^** | |
| --- | --- | --- | --- | --- | --- | --- | --- | --- | --- | --- | --- | --- | --- | --- | --- | --- | --- | --- | --- |
|  | T19E7.3 |  |  | *bec-1* | | Beclin-like protein | | | | • | | • | • | |  | • | | • | |
|  | K09A9.3 |  |  | *ent-2* | | Nucleoside transporter | | | | • | | • | • | |  |  | |  | |
|  | C53A5.3 |  |  | *hda-1* | | Histone deacetylase complex | | | | • | | • | • | | • | • | |  | |
|  | F46A9.4 |  |  | *skr-2* | | SCF ubiquitin ligase, Skp1 component | | | | • | | • | • | | • |  | |  | |
|  | C44E4.4 |  |  |  | | RNA-binding protein La | | | | • | | • |  | |  | • | |  | |
|  | C56C10.8 |  |  | *icd-1* | | RNA polymerase II general transcription factor | | | | • | | • | • | | • | • | |  | |
|  | T26A5.9 |  |  | *dlc-1* | | Dynein light chain type 1 | | | |  | | • | • | |  |  | |  | |
|  | F43D9.4 |  |  | *sip-1* | | Alpha crystallins | | | |  | |  |  | |  |  | | • | |
|  | K07A1.11 |  |  | *rba-1* | | Nucleosome remodeling factor | | | | • | | • | • | |  |  | |  | |
|  | C06G3.10 |  |  | *cgoc-2* | | Low density lipoprotein receptor | | | |  | | • | • | |  |  | |  | |
|  | B0336.2 |  |  | *arf-1* | | GTP-binding ADP-ribosylation factor Arf1 | | | | • | | • |  | |  |  | |  | |
|  | B0336.6 |  |  | *abi-1* | | Abl interactor, contains SH3 domain | | | | • | | • | • | |  |  | | • | |
|  | C50B8.2 |  |  | *bir-2* | | Apoptosis inhibitor IAP1 | | | |  | |  |  | | • | • | | • | |
|  | F55F8.5 |  |  | *tag-345* | | Microtubule binding protein YTM1 | | | | • | | • | • | |  |  | |  | |
|  | F26E4.6 |  |  |  | | Cytochrome c oxidase, subunit VIIc/COX8 | | | | • | |  |  | |  |  | | • | |
|  | F58E10.5 |  |  | *end-3* | | GATA-4/5/6 transcription factors | | | |  | | • |  | |  |  | |  | |
|  | C24B9.9 |  |  | *dod-3* | | Unnamed protein | | | |  | |  |  | |  |  | | • | |
|  | K04G7.11 |  |  |  | | mRNA splicing factor SYF2 | | | | • | | • | • | |  |  | |  | |
|  | R03E9.1 |  |  | *mdl-1* | | Upstream transcription factor 2/L-myc-2 protein | | | |  | |  |  | | • |  | | • | |
|  | R06C1.1 |  |  | *hda-3* | | Histone deacetylase complex | | | | • | |  |  | | • |  | |  | |
|  | T09A5.10 |  |  | *lin-5* | | Unnamed protein | | | |  | | • |  | |  |  | |  | |
|  | T26G10.1 |  |  |  | | ATP-dependent RNA helicase | | | | • | | • | • | |  |  | |  | |
|  | ZK938.5 |  |  | *old-2* | | Fibroblast/platelet-derived growth factor receptor | | | |  | |  |  | |  |  | | • | |
|  | T07C4.4 |  |  | *spp-1* | | Unnamed protein | | | |  | |  |  | |  |  | | • | |
|  | F18C5.2 |  |  | *wrn-1* | | ATP-dependent DNA helicase | | | | • | |  |  | |  |  | | • | |
|  | C50E10.4 |  |  | *sop-2* | | Unnamed protein | | | | • | |  |  | | • |  | |  | |
|  | F56A12.1 |  |  | *unc-39* | | Transcription factor SIX | | | | • | | • |  | |  |  | |  | |
|  | C43H8.1 |  |  |  | | Uncharacterized conserved protein | | | | • | | • | • | |  |  | |  | |
|  | R02D3.5 |  |  |  | | Protein farnesyltransferase | | | | • | | • | • | |  |  | |  | |
|  | F21C3.5 |  |  | *pfd-6* | | Prefoldin subunit 6, KE2 family | | | | • | | • | • | |  |  | |  | |
|  | C09H10.7 |  |  |  | | Unnamed protein | | | | • | | • |  | |  |  | |  | |
|  | C15H7.4 |  |  |  | | Unnamed protein | | | | • | | • | • | |  |  | |  | |
|  | W03H9.4 |  |  | *cacn-1* | | Cactin | | | | • | | • | • | |  |  | |  | |
|  | Y105E8A.17 |  |  | *ekl-4* | | DNA methyltransferase 1-associated protein-1 | | | | • | |  |  | | • |  | |  | |
|  | Y111B2A.15 |  |  | *tpst-1* | | Protein-tyrosine sulfotransferase | | | | • | | • | • | |  |  | |  | |
|  | ZK809.4 |  |  | *ent-1* | | Nucleoside transporter | | | | • | | • | • | |  |  | |  | |
|  | ZK1128.3 |  |  |  | | Unnamed protein | | | | • | | • | • | |  |  | |  | |
|  | C07H6.7 |  |  | *lin-39* | | Transcription factor zerknullt | | | | • | |  |  | |  | • | |  | |
|  | C44C10.8 |  |  | *hnd-1* | | Uncharacterized conserved protein | | | | • | | • |  | |  |  | |  | |
|  | F12F6.7 |  |  |  | | DNA polymerase delta, regulatory subunit 55 | | | | • | | • | • | |  |  | |  | |
|  | F15D3.7 |  |  |  | | Mitochondria import inner membrane translocase | | | | • | | • | • | |  |  | |  | |
|  | R06A10.2 |  |  | *gsa-1* | | G protein subunit | | | | • | | • | • | |  |  | |  | |
|  | Y55D5A.5 |  |  | *daf-2* | | Insulin/growth factor receptor | | | | • | |  |  | |  | • | | • | |
|  | C01C7.1 |  |  | *ark-1* | | ACK and related non-receptor tyrosine kinases | | | |  | | • |  | | • | • | |  | |
|  | F48C1.4 |  |  |  | | Unnamed protein | | | | • | | • | • | |  |  | |  | |
|  | T07C4.8 |  |  | *ced-9* | | Anti-apoptotic Bcl-2 family proteins | | | |  | |  |  | | • | • | |  | |
|  | Y51A2D.15 |  |  |  | | Uncharacterized coiled-coil protein | | | | • | | • | • | |  |  | |  | |
|  | K12G11.4 |  |  | *sodh-2* | | Alcohol dehydrogenase, class V | | | |  | |  |  | |  |  | | • | |
|  | B0495.4 |  |  | *nhx-2* | | Sodium/hydrogen exchanger protein | | | | • | |  |  | |  |  | | • | |
|  | ZK632.13 |  |  | *lin-52* | | Uncharacterized conserved protein | | | | • | | • |  | | • | • | |  | |
|  | T20B12.8 |  |  | *hmg-4* | | Nucleosome-binding factor SPN | | | | • | | • | • | |  |  | |  | |
|  | F35H10.5 |  |  |  | | Uncharacterized protein | | | |  | |  |  | |  |  | | • | |
|  | F55C5.8 |  |  |  | | Signal recognition particle, subunit Srp68 | | | | • | | • | • | |  |  | |  | |
|  | ZK1251.9 |  |  |  | | HIV-1 Vpr-binding protein | | | | • | | • | • | |  |  | |  | |
|  | R07E5.14 |  |  | *rnp-4* | | RNA-binding protein RBM8/Tsunagi | | | | • | | • | • | |  |  | |  | |
|  | T05A6.1 |  |  | *cki-1* | | Cyclin-dependent kinase inhibitor | | | | • | | • |  | | • |  | |  | |
|  | F55G11.5 |  |  | *dod-22* | | Uncharacterized protein | | | |  | |  |  | |  |  | | • | |
|  | C50A2.2 |  |  |  | | Unnamed protein | | | | • | | • | • | |  |  | |  | |
| **<0.2**  **0.2** | | **<0.3** | | | **<0.5** | | **<0.7** | **<...>** | **>1.4** | | **>2.0** | | | **>3.0** | | | **>4.0** | |  |

^1^GO terms associated with reproduction (50 in total), ^2^GO:0048513, ^3^GO:0040035, ^4^GO:0048523, ^5^GO:0051093, ^6^GO:0008340
