# Supplementary material for: Gene expression profiling to characterize sediment toxicity – a pilot study using Caenorhabditis elegans whole genome microarrays
Source: BMC Genomics. 2009 Apr 14;10:160. doi: 10.1186/1471-2164-10-160 (PMC2674462; doi:10.1186/1471-2164-10-160)
Supplement: Additional file 8 — Overlapping differentially regulated genes of selected toxico- genomic studies in C. elegans. C. elegans' transcripts significantly changed in response to sediment exposures (this study) and PCB52 [11], Cd [12] and two humic substances [10]. [file 1471-2164-10-160-S8.doc]

### Additional file 8 – Overlapping differentially regulated genes of selected toxico- genomic studies in *C. elegans*

*C. elegans’* transcripts significantly changed in response to sediment exposures (this study) and PCB52 [11], Cd [12] and two humic substances [10].

| **ID** | **Elbe sediment** | **Rhine sediment** | **PCB52 [11]** | **Cd [12]** | **HS1500 [10]** | **HS Fuchs-kuhle [10]** | **CGC name** | **Description** |
| --- | --- | --- | --- | --- | --- | --- | --- | --- |
| **UP-REGULATED GENES** | | | | | | | | |
| F28F8.2 | **×** |  | **×** | **×** |  |  | [*acs-2*](http://www.wormbase.org/db/gene/gene?name=acs-2;class=Gene) | Long chain fatty acid acyl-CoA ligase |
| W03D2.7 | **×** |  | **×** | **×** |  |  |  | unknown |
| F17H10.1 | **×** | **×** | **×** |  |  |  |  | uncharacterized conserved protein |
| T05H4.4 | **×** | **×** | **×** |  |  |  |  | NADH-cytochrome b-5 reductase |
| T26E4.7 | **×** | **×** | **×** |  |  |  |  | unknown |
| T28A11.2 | **×** | **×** | **×** |  |  |  |  | predicted secreted cysteine rich protein |
| W09H1.1 | **×** | **×** | **×** |  |  |  |  | unknown |
| Y116F11B.3 | **×** | **×** | **×** |  |  |  | [*pcp-4*](http://www.wormbase.org/db/gene/gene?name=pcp-4;class=Gene) | hydrolytic enzymes of the alpha/beta hydrolase fold |
| C52E12.1 | **×** |  | **×** |  |  |  |  | Zinc finger type |
| F18A1.5 | **×** |  | **×** |  |  |  | [*rpa-1*](http://www.wormbase.org/db/gene/gene?name=rpa-1;class=Gene) | Single-stranded DNA-binding replication protein A |
| F18C5.2 | **×** |  | **×** |  |  |  | [*wrn-1*](http://www.wormbase.org/db/gene/gene?name=wrn-1;class=Gene) | ATP-dependent DNA helicase |
| F19B2.5 | **×** |  | **×** |  |  |  |  | Helicase-like transcription factor |
| F42H10.9 | **×** |  | **×** |  |  |  |  | unknown |
| F47G9.6 | **×** |  | **×** |  |  |  |  | unknown |
| F48A9.2 | **×** |  | **×** |  |  |  |  | unknown |
| F53A9.8 | **×** |  | **×** |  |  |  |  | unknown |
| F55H12.3 | **×** |  | **×** |  |  |  |  | unknown |
| H10D12.2 | **×** |  | **×** |  |  |  |  | unknown |
| H11E01.1 | **×** |  | **×** |  |  |  | [*fbxb-58*](http://www.wormbase.org/db/gene/gene?name=fbxb-58;class=Gene) | predicted transposase |
| H12I19.1 | **×** |  | **×** |  |  |  | [*srz-42*](http://www.wormbase.org/db/gene/gene?name=srz-42;class=Gene) | 7-transmembrane receptor |
| K02D10.1 | **×** |  | **×** |  |  |  |  | NIPSNAP1 protein |
| K06B4.8 | **×** |  | **×** |  |  |  | [*nhr-198*](http://www.wormbase.org/db/gene/gene?name=nhr-198;class=Gene) | nuclear hormone receptor |
| K06H6.4 | **×** |  | **×** |  |  |  |  | extracellular protein with conserved cysteines |
| K10H10.2 | **×** |  | **×** |  |  |  |  | cystathionine beta-synthase and related enzymes |
| K12D9.5 | **×** |  | **×** |  |  |  | [*srw-120*](http://www.wormbase.org/db/gene/gene?name=srw-120;class=Gene) | 7-transmembrane olfactory receptor |
| M01G12.13 | **×** |  | **×** |  |  |  | [*sri-13*](http://www.wormbase.org/db/gene/gene?name=sri-13;class=Gene) | predicted olfactory G-protein coupled receptor |
| R04B5.8 | **×** |  | **×** |  |  |  | [*srd-49*](http://www.wormbase.org/db/gene/gene?name=srd-49;class=Gene) | chemoreceptor/7TM receptor |
| T05C7.1 | **×** |  | **×** |  |  |  |  | unknown |
| T06F4.1 | **×** |  | **×** |  |  |  |  | unknown |
| T15B7.8 | **×** |  | **×** |  |  |  |  | uncharacterized conserved protein |
| T21H3.5 | **×** |  | **×** |  |  |  |  | unknown |
| T23D8.9 | **×** |  | **×** |  |  |  | [*sys-1*](http://www.wormbase.org/db/gene/gene?name=sys-1;class=Gene) | novel protein that contains three divergent armadillo repeats |
| Y46C8AL.4 | **×** |  | **×** |  |  |  | [*clec-71*](http://www.wormbase.org/db/gene/gene?name=clec-71;class=Gene) | C-type lectin |
| Y46H3C.1 | **×** |  | **×** |  |  |  | [*srw-100*](http://www.wormbase.org/db/gene/gene?name=srw-100;class=Gene) | 7-transmembrane olfactory receptor |
| Y51H7C.10 | **×** |  | **×** |  |  |  |  | unknown |
| ZK858.5 | **×** |  | **×** |  |  |  |  | unknown |
| F44F1.1 |  | **×** | **×** |  |  |  |  | unknown |
| F56A12.1 |  | **×** | **×** |  |  |  | *unc-39* | transcription factor SIX, HOX domain protein |
| K06B4.5 |  | **×** | **×** |  |  |  | *nhr-196* | nuclear hormone receptor |
| K07C6.5 |  | **×** | **×** |  |  |  | *cyp-35A5* | cytochrome-P450 |
| K12H6.4 |  | **×** | **×** |  |  |  |  | unknown |
| M01G12.11 |  | **×** | **×** |  |  |  |  | unknown |
| M02F4.3 |  | **×** | **×** |  |  |  |  | predicted membrane protein, contains two CBS domains |
| R04D3.3 |  | **×** | **×** |  |  |  |  | unknown |
| T12C9.7 |  | **×** | **×** |  |  |  |  | unknown |
| T27A10.6 |  | **×** | **×** |  |  |  |  | unknown |
| T27E4.6 |  | **×** | **×** |  |  |  |  | predicted acyltransferase |
| W05B2.4 |  | **×** | **×** |  |  |  |  | Dyneins, heavy chain |
| W06F12.1 |  | **×** | **×** |  |  |  | *lit-1* | Nemo-like MAPK-related serine/threonine protein kinase |

| Y119D3A.4 |  | **×** | **×** |  |  |  |  |  |
| --- | --- | --- | --- | --- | --- | --- | --- | --- |
| Y46G5A.26 |  | **×** | **×** |  |  |  | *lgc-35* | GABA receptor |
| Y53F4B.17 |  | **×** | **×** |  |  |  |  | unknown |
| Y54F10BM.1 |  | **×** | **×** |  |  |  | *fbxa-1* | serine/threonine protein phosphatase |
| Y54G2A.21 |  | **×** | **×** |  |  |  |  | unknown |
| Y71D11A.1 |  | **×** | **×** |  |  |  | *cdh-12* | cadherin |
| Y71H2AR.2 |  | **×** | **×** |  |  |  |  | cysteine proteinase Cathepsin L |
| ZC196.9 |  | **×** | **×** |  |  |  |  | membrane protein |
| ZK546.14 |  | **×** | **×** |  |  |  |  | uncharacterized conserved protein |
| T08G5.1 | **×** | **×** |  | **×** |  |  |  | unknown |
| C18A11.1 | **×** |  |  | **×** |  |  |  | unknown |
| C32F10.4 | **×** |  |  | **×** |  |  |  | unknown |
| C45G7.3 | **×** |  |  | **×** |  |  | [*ilys-3*](http://www.wormbase.org/db/gene/gene?name=ilys-3;class=Gene) | predicted as invertebrate lysozyme |
| F22B7.9 | **×** |  |  | **×** |  |  |  | predicted methyltransferase |
| K01A2.2 | **×** |  |  | **×** |  |  | [*far-7*](http://www.wormbase.org/db/gene/gene?name=far-7;class=Gene) | fatty-acid and retinol-binding protein |
| W01A11.1 | **×** |  |  | **×** |  |  |  | predicted hydrolases or acyltransferases |
| F53C3.12 |  | **×** |  | **×** |  |  | [*bcmo-2*](http://www.wormbase.org/db/gene/gene?name=bcmo-2;class=Gene) | beta-carotene 15,15'-dioxygenase |
| K03E5.2 | **×** | **×** |  |  | **×** |  |  | unknown |
| F11G11.2 | **×** |  |  |  | **×** |  | [*gst-7*](http://www.wormbase.org/db/gene/gene?name=gst-7;class=Gene) | glutathione S-transferase |
| F28B3.10 | **×** |  |  |  | **×** |  |  | unknown |
| F43D9.4 | **×** |  |  |  | **×** |  | [*sip-1*](http://www.wormbase.org/db/gene/gene?name=sip-1;class=Gene) | alpha crystalline, small heat shock protein |
| F54F2.2 | **×** |  |  |  | **×** |  | [*zfp-1*](http://www.wormbase.org/db/gene/gene?name=zfp-1;class=Gene) | PHD zinc finger protein AF10 |
| F59A1.11 | **×** |  |  |  | **×** |  |  | unknown |
| K08A2.2 | **×** |  |  |  | **×** |  |  | unknown |
| T01C4.1 | **×** |  |  |  | **×** |  |  | chitinase |
| C52B11.3 |  | **×** |  |  | **×** |  | [*dop-4*](http://www.wormbase.org/db/gene/gene?name=dop-4;class=Gene) | G-protein coupled receptor |
| H05C05.2 |  | **×** |  |  | **×** |  |  | unknown |
| H14E04.4 |  | **×** |  |  | **×** |  |  | unknown |
| K10B3.6 |  | **×** |  |  | **×** |  |  | predicted starch-binding protein |
| T04C12.2 |  | **×** |  |  | **×** |  | [*srh-75*](http://www.wormbase.org/db/gene/gene?name=srh-75;class=Gene) | predicted olfactory G-protein coupled receptor |
| Y39A1C.4 |  | **×** |  |  | **×** |  | [*hex-3*](http://www.wormbase.org/db/gene/gene?name=hex-3;class=Gene) | beta-N-acetylhexosaminidase |
| C10A4.2 | **×** |  |  |  |  | **×** |  | unknown |
| F37C12.4 | **×** |  |  |  |  | **×** | [*rpl-36*](http://www.wormbase.org/db/gene/gene?name=rpl-36;class=Gene) | 60S ribosomal protein L36 |
| F56C3.6 | **×** |  |  |  |  | **×** | [*dgn-2*](http://www.wormbase.org/db/gene/gene?name=dgn-2;class=Gene) | Dystroglycan |
| H22D07.1 | **×** |  |  |  |  | **×** | [*gnrr-5*](http://www.wormbase.org/db/gene/gene?name=gnrr-5;class=Gene) | 7 transmembrane receptor |
| K02B12.8 | **×** |  |  |  |  | **×** | [*zhp-3*](http://www.wormbase.org/db/gene/gene?name=zhp-3;class=Gene) | RING finger protein involved in synaptonemal complex formation |
| T26G10.1 | **×** |  |  |  |  | **×** |  | ATP-dependent RNA helicase |
| C04G2.1 |  | **×** |  |  |  | **×** | [*ttr-39*](http://www.wormbase.org/db/gene/gene?name=ttr-39;class=Gene) | uncharacterized protein with conserved cysteine |
| C13C4.3 |  | **×** |  |  |  | **×** | [*nhr-136*](http://www.wormbase.org/db/gene/gene?name=nhr-136;class=Gene) | nuclear hormone receptor |
| C54D1.2 |  | **×** |  |  |  | **×** | [*clec-86*](http://www.wormbase.org/db/gene/gene?name=clec-86;class=Gene) | C-type lectin |
| F48D6.4 |  | **×** |  |  |  | **×** |  | unknown |
| R02D3.5 |  | **×** |  |  |  | **×** |  | protein farnesyltransferase |
| Y39G10AR.17 |  | **×** |  |  |  | **×** |  | unknown |

| **ID** | **Elbe sediment** | **Rhine sediment** | **PCB52 [11]** | **Cd [12]** | **HS1500 [10]** | **HS Fuchs-kuhle [10]** | **CGC name** | **Description** |
| --- | --- | --- | --- | --- | --- | --- | --- | --- |
| **DOWN-REGULATED GENES** | | | | | | | | |
| T09F5.9 | **×** | **×** | **×** | **×** |  |  | *clec-47* | C-type lectin |
| C03G6.5 | **×** | **×** | **×** |  |  |  |  | unknown |
| B0302.1 | **×** |  | **×** |  |  |  | *kin-25* | CK and related non-receptor tyrosine kinases |
| C16C2.3 | **×** |  | **×** |  |  |  | *ocrl-1* | inositol-1,4,5-triphosphate 5-phosphatase |
| F57B9.3 | **×** |  | **×** |  |  |  | *phi-2* | translation initiation factor 4F |
| M02A10.1 | **×** |  | **×** |  |  |  |  | unknown |
| Y92H12BL.4 | **×** |  | **×** |  |  |  |  | unknown |
| ZK632.13 | **×** |  | **×** |  |  |  | *lin-52* | uncharacterized conserved protein |
| C24A3.4 |  | **×** | **×** |  |  |  |  | unknown |
| C27D6.6 |  | **×** | **×** |  |  |  | *srb-5* | Sra family integral membrane protein |
| C44E4.1 |  | **×** | **×** |  |  |  |  | Zn-binding protein Push |
| F07C3.2 |  | **×** | **×** |  |  |  |  | unknown |
| F15H10.2 |  | **×** | **×** |  |  |  | *col-13* | Collagen (type IV and type XIII) |
| F21H7.2 |  | **×** | **×** |  |  |  |  | unknown |
| F33H2.6 |  | **×** | **×** |  |  |  |  | uncharacterized conserved protein |
| F44C8.6 |  | **×** | **×** |  |  |  | *nhr-56* | nuclear hormone receptor |
| F48C1.8 |  | **×** | **×** |  |  |  |  | unknown |
| K12D12.1 |  | **×** | **×** |  |  |  | *top-2* | DNA topoisomerase type II |
| M01E5.3 |  | **×** | **×** |  |  |  |  | unknown |
| Y105E8A.16 |  | **×** | **×** |  |  |  | *rps-20* | 40S ribosomal protein S20 |
| F17E5.1 | **×** | **×** |  |  | **×** | **×** | *lin-2* | Ca^2+^/calmodulin-dependent protein kinase |
| C03H5.5 | **×** |  |  |  | **×** |  |  | RNA polymerase I-associated factor - PAF67 |
| F01G4.1 | **×** |  |  |  | **×** |  | *psa-4* | chromatin remodeling complex SWI/SNF |
| F11A1.3 | **×** |  |  |  | **×** |  | *daf-12* | steroid hormone receptor |
| F40F12.5 | **×** |  |  |  | **×** |  | *cyld-1* | familial cylindromatosis protein |
| M02B1.2 | **×** |  |  |  | **×** |  |  | unknown |
| R09A8.1 | **×** |  |  |  | **×** |  |  | unknown |
| Y39A3B.1 | **×** |  |  |  | **×** |  |  | aminopeptidases of the M20 family |
| Y44A6D.5 | **×** |  |  |  | **×** |  |  | branched chain aminotransferase BCAT1 |
| C02F12.1 |  | **×** |  |  | **×** |  | *tsp-17* | tetraspanin family integral membrane protein |
| C05E7.3 |  | **×** |  |  | **×** |  |  | unknown |
| C08H9.1 |  | **×** |  |  | **×** |  |  | Serine carboxypeptidases (lysosomal cathepsin A) |
| C38C3.6 |  | **×** |  |  | **×** |  |  | unknown |
| C46A5.6 |  | **×** |  |  | **×** |  |  | unknown |
| D2085.7 |  | **×** |  |  | **×** |  |  | uncharacterized conserved protein |
| F17E5.2 |  | **×** |  |  | **×** |  |  | predicted mitochondrial carrier protein |
| F20A1.7 |  | **×** |  |  | **×** |  | *twk-11* | two-P domain potassium channel protein |
| F55C12.4 |  | **×** |  |  | **×** |  |  | unknown |
| T22H2.1 |  | **×** |  |  | **×** |  | *sri-12* | 7 transmembrane receptor |
| Y57G11B.3 |  | **×** |  |  | **×** |  |  | unknown |
| B0213.14 | **×** | **×** |  |  |  | **×** | *cyp-34A8* | cytochrome P450 |
| C06B8.2 | **×** |  |  |  |  | **×** |  | unknown |
| F07A5.4 | **×** |  |  |  |  | **×** |  | unknown |
| C06C6.5 |  | **×** |  |  |  | **×** | *nhr-50* | nuclear hormone receptor |
| C01G10.3 |  | **×** |  |  |  | **×** | *srx-78* | 7 transmembrane receptor |
