# Supplementary material for: Gene expression profiling to characterize sediment toxicity – a pilot study using Caenorhabditis elegans whole genome microarrays
Source: BMC Genomics. 2009 Apr 14;10:160. doi: 10.1186/1471-2164-10-160 (PMC2674462; doi:10.1186/1471-2164-10-160)
Supplement: Additional file 9 — Principal component analysis (PCA) including a liquid medium laboratory control. PCA of significantly changing genes in C. elegans exposed to three river sample sediments: Danube (red), Elbe (yellow) and Rhine (blue) or S-basal, an artificial laboratory control (black). Note that PCA analysis identifies that the laboratory control exposure clusters distinctly separate from the three sediment exposures when laboratory control (A) or the Danube sediment (B) is used as the baseline dataset. [file 1471-2164-10-160-S9.doc]

### Additional file 9 – Principal component analysis (PCA) including a liquid medium laboratory control

PCA of significantly changing genes in *C.elegans* exposed to three river sample sediments: Danube (red), Elbe (yellow) and Rhine (blue) or S-basal, an artificial laboratory control (black). Note that PCA analysis identifies that the laboratory control exposure clusters distinctly separate from the three sediment exposures when laboratory control (**A**) or the Danube sediment (**B**) is used as the baseline dataset.
